# Comparison of long-read methods for sequencing and assembly of a plant genome

**DOI:** 10.1093/gigascience/giaa146

**Published:** 2020-12-21

**Authors:** Valentine Murigneux, Subash Kumar Rai, Agnelo Furtado, Timothy J C Bruxner, Wei Tian, Ivon Harliwong, Hanmin Wei, Bicheng Yang, Qianyu Ye, Ellis Anderson, Qing Mao, Radoje Drmanac, Ou Wang, Brock A Peters, Mengyang Xu, Pei Wu, Bruce Topp, Lachlan J M Coin, Robert J Henry

**Affiliations:** Genome Innovation Hub, The University of Queensland, 306 Carmody Road, Brisbane, QLD 4072, Australia; Institute for Molecular Bioscience, The University of Queensland, 306 Carmody Road, Brisbane, QLD 4072, Australia; Genome Innovation Hub, The University of Queensland, 306 Carmody Road, Brisbane, QLD 4072, Australia; Institute for Molecular Bioscience, The University of Queensland, 306 Carmody Road, Brisbane, QLD 4072, Australia; Queensland Alliance for Agriculture and Food Innovation, The University of Queensland, Brisbane, QLD 4072, Australia; Institute for Molecular Bioscience, The University of Queensland, 306 Carmody Road, Brisbane, QLD 4072, Australia; BGI-Shenzhen, No.21 Hongan 3rd Street, Yantian District, Shenzhen 518083, China; BGI-Australia, 300 Herston Road, Herston, QLD 4006, Australia; BGI-Shenzhen, No.21 Hongan 3rd Street, Yantian District, Shenzhen 518083, China; BGI-Australia, 300 Herston Road, Herston, QLD 4006, Australia; BGI-Shenzhen, No.21 Hongan 3rd Street, Yantian District, Shenzhen 518083, China; MGI, BGI-Shenzhen, Building 11, Beishan Industrial Zone, Yantian District, Shenzhen 518083, China; BGI-Shenzhen, No.21 Hongan 3rd Street, Yantian District, Shenzhen 518083, China; BGI-Australia, 300 Herston Road, Herston, QLD 4006, Australia; BGI-Shenzhen, No.21 Hongan 3rd Street, Yantian District, Shenzhen 518083, China; BGI-Australia, 300 Herston Road, Herston, QLD 4006, Australia; MGI, BGI-Shenzhen, Building 11, Beishan Industrial Zone, Yantian District, Shenzhen 518083, China; Advanced Genomics Technology Lab, Complete Genomics Inc., 2904 Orchard Parkway, San Jose, CA 95134, USA; MGI, BGI-Shenzhen, Building 11, Beishan Industrial Zone, Yantian District, Shenzhen 518083, China; Advanced Genomics Technology Lab, Complete Genomics Inc., 2904 Orchard Parkway, San Jose, CA 95134, USA; BGI-Shenzhen, No.21 Hongan 3rd Street, Yantian District, Shenzhen 518083, China; MGI, BGI-Shenzhen, Building 11, Beishan Industrial Zone, Yantian District, Shenzhen 518083, China; Advanced Genomics Technology Lab, Complete Genomics Inc., 2904 Orchard Parkway, San Jose, CA 95134, USA; BGI-Shenzhen, No.21 Hongan 3rd Street, Yantian District, Shenzhen 518083, China; BGI-Shenzhen, No.21 Hongan 3rd Street, Yantian District, Shenzhen 518083, China; MGI, BGI-Shenzhen, Building 11, Beishan Industrial Zone, Yantian District, Shenzhen 518083, China; Advanced Genomics Technology Lab, Complete Genomics Inc., 2904 Orchard Parkway, San Jose, CA 95134, USA; BGI-Shenzhen, No.21 Hongan 3rd Street, Yantian District, Shenzhen 518083, China; BGI-Qingdao, Building 2, No. 2 Hengyunshan Road, Qingdao 266555, China; BGI-Shenzhen, No.21 Hongan 3rd Street, Yantian District, Shenzhen 518083, China; BGI-Tianjin, Airport Business Park, Building E3, Airport Economics Area, Tianjin 300308, China; Queensland Alliance for Agriculture and Food Innovation, The University of Queensland, Brisbane, QLD 4072, Australia; Genome Innovation Hub, The University of Queensland, 306 Carmody Road, Brisbane, QLD 4072, Australia; Institute for Molecular Bioscience, The University of Queensland, 306 Carmody Road, Brisbane, QLD 4072, Australia; Department of Microbiology and Immunology, University of Melbourne at The Peter Doherty Institute for Infection and Immunity, 792 Elizabeth Street, Melbourne, VIC 3004, Australia; Queensland Alliance for Agriculture and Food Innovation, The University of Queensland, Brisbane, QLD 4072, Australia

**Keywords:** assembly, long reads, PacBio, Pacific Biosciences, Sequel, Oxford Nanopore Technologies, PromethION, BGI, single-tube long fragment read, stLFR, ONT

## Abstract

**Background:**

Sequencing technologies have advanced to the point where it is possible to generate high-accuracy, haplotype-resolved, chromosome-scale assemblies. Several long-read sequencing technologies are available, and a growing number of algorithms have been developed to assemble the reads generated by those technologies. When starting a new genome project, it is therefore challenging to select the most cost-effective sequencing technology, as well as the most appropriate software for assembly and polishing. It is thus important to benchmark different approaches applied to the same sample.

**Results:**

Here, we report a comparison of 3 long-read sequencing technologies applied to the *de novo* assembly of a plant genome, *Macadamia jansenii*. We have generated sequencing data using Pacific Biosciences (Sequel I), Oxford Nanopore Technologies (PromethION), and BGI (single-tube Long Fragment Read) technologies for the same sample. Several assemblers were benchmarked in the assembly of Pacific Biosciences and Nanopore reads. Results obtained from combining long-read technologies or short-read and long-read technologies are also presented. The assemblies were compared for contiguity, base accuracy, and completeness, as well as sequencing costs and DNA material requirements.

**Conclusions:**

The 3 long-read technologies produced highly contiguous and complete genome assemblies of *M. jansenii*. At the time of sequencing, the cost associated with each method was significantly different, but continuous improvements in technologies have resulted in greater accuracy, increased throughput, and reduced costs. We propose updating this comparison regularly with reports on significant iterations of the sequencing technologies.

## Introduction

Advances in DNA sequencing enable the rapid analysis of genomes, driving biological discovery. Sequencing of complex genomes, which are very large and have a high content of repetitive sequences or many copies of similar sequences, remains challenging. Many plant genomes are complex, and the quality of published sequences remains relatively poor. However, improvements in long-read sequencing are making it easier to generate high-quality sequences for complex genomes.

We now report a comparison of 3 long-read sequencing methods applied to the *de novo* sequencing of a plant, *Macadamia jansenii*. This is a rare species that is a close relative of the macadamia nut recently domesticated in Hawaii and Australia. In the wild, it grows as a multi-stemmed, evergreen tree reaching 6–9 m height with leaves having entire margins and generally in whorls of 3. The nuts are small (11–16 mm diameter) and have a smooth, hard, brown shell that encloses a cream, globulose kernel that is bitter and inedible [1]. The species was discovered as a single population of ∼60 plants in the wild in eastern Australia [2]. This is a flowering plant (angiosperm) in the Proteaceae family that is basal to the large eudicot branch of the flowering plant phylogeny [3]. The genomes of this group are poorly characterized, with most well-sequenced plant genomes being either core eudicots or monocots that are plants of economic importance [4]. Knowledge of the genome of this species will support efforts to conserve endangered species in the wild and capture novel traits such as small plant stature for use in plant breeding. Sequencing of wild crop relatives is urgent because many populations are critical to diversification of crop genetics to ensure food security in response to climate change [5] but are also threatened with extinction due to changes in land use or climate [6].

The macadamia genus contains 4 species: *Macadamia integrifolia, Macadamia tetraphylla, Macadamia ternifolia*, and *Macadamia jansenii*. Macadamia cultivars are diploid (2n = 28), with *k*-mer–based genome size estimates ranging from 758 Mb for *M. tetraphylla* [7] to 896 Mb for *M. integrifolia* [8]. The first draft genome assembly of the widely grown *M. integrifolia* cultivar HAES 741 was constructed from short-read Illumina sequence data and was highly fragmented (518 Mb, 193,493 scaffolds, N50 = 4,745 bp) [9]. An improved HAES 741 assembly was generated using a combination of long-read Pacific Biosciences (PacBio) and paired-end Illumina sequence data (745 Mb, 4,094 scaffolds, N50 = 413 kb) [8]. The genome assembly of *M. tetraphylla* was also recently produced using a combination of long-read Oxford Nanopore Technologies (ONT) and short-read Illumina sequence data (751 Mb, 4,335 contigs, N50 = 1.18 Mb) [7].

Long-read sequencing provides data that facilitate easier assembly of the genome than is possible with short reads [10–12]. The length and sequence quality delivered by the available sequencing platforms has continued to improve. The reads produced can be used to assemble contigs or as a scaffold for the assembly of contigs generated with these techniques or from short reads [13]. Currently, PacBio and ONT are the most commonly used technologies to generate long reads. Single-molecule real-time (SMRT) sequencing, developed by PacBio, can generate reads in the tens of kilobases using the continuous long-read sequencing mode, thus enabling high-quality *de novo* genome assembly. ONT enables direct and real-time sequencing of long DNA or RNA fragments by analysing the electrical current disruption caused by the molecules as they move through a protein nanopore. More recently, BGI has introduced the single-tube Long Fragment Read (stLFR) [14] technology as an alternative to the generation of real long reads. stLFR is based on DNA co-barcoding [15,16], i.e., adding the same barcode sequence to subfragments from the original long DNA molecule. In the stLFR process, the surfaces of microbeads are used to create millions of miniaturized barcoding reactions in a single tube. Importantly, stLFR enables near single-molecule co-barcoding by using a large excess of microbeads and a combinatorial process to make ∼3.6 billion unique barcode sequences. For this reason it is expected to enable high-quality and near-complete *de novo* assemblies. Here we compare Sequel I (PacBio), PromethION (ONT), and stLFR (BGI) data for the same DNA sample and evaluate the quality of the assemblies that can be generated directly from these datasets.

## Methods

### Plant material

Young leaves (40 g) of *M. jansenii* were sourced from a tree with accession No. 1005 and located at the Maroochy Research Facility, Department of Agriculture and Fisheries, Nambour 4560, Queensland, Australia. The specimen of *M. jansenii* used in these experiments was a clonally propagated *ex situ* tree planted in the arboretum at Maroochy Research Facility. None of the leaves used in these experiments were collected from wild *in situ* trees. Young leaves were harvested, placed in on ice in bags, and within 3 h snap-frozen under liquid nitrogen and stored at −20°C until further processed for tissue pulverization using either a mortar and pestle or the Mixer Mill as outlined below.

### Genomic DNA extraction

Leaf tissue (10 g) was first coarsely ground under liquid nitrogen using a mortar and pestle. The mortar and pestle with the coarsely ground tissue with residual liquid nitrogen was then placed on dry ice. This step ensured that the temperature of the coarsely ground tissue was maintained close to −80°C while allowing the liquid nitrogen to evaporate off completely, an essential requirement for the pulverization step. The coarsely ground leaf tissue was pulverized into fine powder in 50-mL steel jars using the Mixer Mill MM400 (Retsch, Germany). The pulverized leaf tissue was stored at −20°C until further required for DNA extraction. Genomic DNA (gDNA) was isolated from pulverized leaf tissue according to [17], with some modifications. Using a liquid nitrogen–cooled spatula, frozen pulverized leaf tissue (3 g) was added to 50-mL tubes (Corning or Falcon) containing warm (40°C) nuclear lysis buffer (8 mL) and 5% sarkosyl solution (5 mL). Tubes were incubated at 40°C for 45 min with periodic (every 5 min) gentle mixing by inverting the tubes. RNA was digested by adding RNase solution (10 mg/mL), the contents gently mixed by inverting the tubes followed by incubation at room temperature for 10 min. Two chloroform extractions were undertaken as follows. Chloroform (10 mL) was added to the tubes and gently mixed by inverting the tubes 50 times. The tubes were centrifuged at 3,500*g* for 5 min in a swing-out bucket rotor. The supernatant was transferred into fresh 50-mL tubes and the chloroform extraction repeated twice. The supernatant was transferred to fresh 50-mL tubes and the DNA precipitated using isopropanol. For every 1 mL of the supernatant, 0.6 mL of isopropanol was added, and the content gently mixed by inverting the tubes 20–25 times. The tubes were incubated at room temperature for 15 min and then centrifuged at 3,500*g* for 5 min in a swing-out bucket rotor. The supernatant was discarded and the DNA pellet was washed of any co-precipitated salts by adding 10 mL of 70% ethanol and incubating the tubes at room temperature for 30 min. The tubes were centrifuged at 3,500*g* for 5 min in a swing-out bucket rotor, the supernatant discarded, and the DNA pellet semi-dried to remove any residual 70% ethanol by incubating the tubes for 10 min upside down over filter paper. The DNA was dissolved by adding 100 μL of TE buffer and then adding incremental 50 μL of TE buffer where required. The DNA solution was transferred to 2-mL nuclease-free tubes and then centrifuged at 14,000*g* for 45 min in a tabletop centrifuge. The supernatant was carefully transferred to fresh 2-mL tubes and the quality checked on a spectrophotometer, and the DNA was resolved on a 0.7% agarose gel. The DNA was then stored at −20°C until used for sequencing.

### PacBio gDNA library preparation and sequencing

DNA sequencing libraries were prepared using the Template Prep Kit 1.0-SPv3 (PacBio, 100-991-900) according to the protocol for >30 kb SMRTbell Libraries (PacBio, Part No. PN 101-024-600 Version 05). Genomic DNA (15 μg) was not fragmented and was instead just purified with AMPure PB beads. The purified gDNA (10 μg) was treated with Exonuclease VII, followed by a DNA damage repair reaction, an end-repair reaction, and purification with AMPure PB beads. Adapters were ligated to the purified, blunt-ended DNA fragments in an overnight incubation. The adapter-ligated sample was digested with Exonuclease III and Exonuclease VII to remove failed ligation products, followed by purification with AMPure PB beads. The purified sample was size selected using the Blue Pippin with a dye-free, 0.75% agarose cassette and U1 marker (Sage Science, BUF7510, Mulgrave, Victoria, Australia) and the 0.75% DF Marker U1 high-pass 30–40 kb vs3 run protocol, with a BPstart cut-off of 35,000 bp. After size selection, the samples were purified with AMPure PB beads, followed by another DNA damage repair reaction, and a final purification with AMPure PB beads. The final purified, size-selected library was quantified on the Qubit fluorometer using the Qubit dsDNA HS assay kit (Invitrogen, Q32854, Thermo Fisher Scientific, Scoresby, Victoria, Australia) to assess the concentration, and a 0.4% Megabase agarose gel (BioRad, 1613108, Gladesville, New South Wales, Australia) to assess the fragment size. Sequencing was performed using the PacBio Sequel I (PacBio Sequel System, RRID:SCR_017989) (software/chemistry v6.0.0). The library was prepared for sequencing according to the SMRT Link sample set-up calculator, following the standard protocol for Diffusion loading with AMPure PB bead purification, using Sequencing Primer v3, Sequel Binding Kit v3.0, and the Sequel DNA Internal Control v3. The polymerase-bound library was sequenced on 8 SMRT Cells with a 10 h movie time using the Sequel Sequencing Kit 3.0 (PacBio, 101-597-900, Mulgrave, Victoria, Australia) and a Sequel SMRT Cell 1M v3 (PacBio, 101-531-000, Mulgrave, Victoria, Australia). Library preparation and sequencing was performed at the Institute for Molecular Bioscience Sequencing Facility (University of Queensland).

### ONT library preparation and sequencing

The quality of the DNA sample was assessed in NanoDrop, Qubit, and the Agilent 4200 TapeStation system. The DNA sample was sequenced on the ONT MinION (MinION, RRID:SCR_017985) and PromethION (PromethION, RRID:SCR_017987). The MinION library was prepared from 1,500 ng input DNA using the ligation sequencing kit (SQK-LSK109, ONT, Oxford, UK) according to the manufacturer’s protocol except the end-repair and end-prep reaction and ligation period were increased to 30 min. Third-party reagents NEBNext end repair/dA-tailing Module (E7546), NEBNext formalin-fixed paraffin-embedded DNA Repair Mix (M6630), and NEB Quick Ligation Module (E6056) were used during library preparation. The adapter-ligated DNA sample was quantified using Qubit^TM^ dsDNA HS Assay Kit (Thermo Fisher Scientific, Scoresby, Victoria, Australia). The MinION flow cell R9.4.1 (FLO-MIN106, ONT, Oxford, UK) was primed according to the manufacturer’s guidelines before loading a library mix (75 μL) containing 438 ng of adapter-ligated DNA, 25.5 μL LB (SQK-LSK109, ONT, Oxford, UK), and 37.5 μL SQB (SQK-LSK109, ONT, Oxford, UK). The MinION sequencing was performed using MinKNOW (v1.15.4), and a standard 48-h run script. Before preparing the PromethION library, short DNA fragments (<10 kb) were first depleted from DNA sample (9 μg) as described in the manufacturer’s instructions for the Short Read Eliminator (SRE) kit (SKU SS-100-101-01, Circulomics Inc, Baltimore, MD, United States). The PromethION library was prepared from 1,200 ng SRE-treated DNA using the ligation sequencing kit (SQK-LSK109, ONT, Oxford, UK). All steps in the library preparation were the same as the MinION library preparation except that the adapter-ligated DNA was eluted in 25 μL of Elution Buffer. The PromethION flow cell (FLO-PRO002, ONT, Oxford, UK) was primed according to the manufacturer’s guidelines before loading a library mix (150 μL) containing 390 ng of adapter-ligated DNA (24 μL), 75 μL of SQB, and 51 μL of LB (SQK-LSK109, ONT, Oxford, UK). Sequencing was performed using MinKNOW (v3.1.23) and a standard 64-h run script. The sequencing run was stopped at 21 h and nuclease flush was performed to recover clogged pores. The Nuclease flushing mix was prepared by mixing 380 μL of Nuclease flush buffer (300 mM KCl, 2 mM CaCl_2_, 10 mM MgCl_2_, 15 mM HEPES pH 8) and 20 μL of DNase I (M0303S, NEB, Notting Hill, Victoria, Australia). The Nuclease flushing mix was loaded into the flow cell and incubated for 30 min. The flow cell was then primed as mentioned above and loaded with the fresh library mix (150 μL) containing 390 ng of adapter-ligated DNA and the standard 64-h run script was rerun using MinKNOW. Refuelling of the sequencing run was performed at each 24 h by adding 150 μL of diluted SQB (1:1, SQB:nuclease-free water) to keep the stable translocation speed of sequencing. ONT fast5 reads were base-called using Guppy v3.0.3 with the config file dna_r9.4.1_450bps_hac_prom.cfg (PromethION) or dna_r9.4.1_450bps_hac.cfg (MinION) and parameters --qscore_filtering -q 0 --recursive --device “cuda:0 cuda:1 cuda:2 cuda:3".

### BGI stLFR library preparation and sequencing

The stLFR sequencing libraries were prepared using the MGIEasy stLFR Library Prep Kit (MGI, Shenzhen, China) following the manufacturer’s protocol. Briefly, genomic DNA samples were serially diluted and then quantified using the Qubit^TM^ dsDNA BR Assay Kit (Invitrogen, Carlsbad, CA) and the Qubit^TM^ dsDNA HS Assay Kit (Invitrogen, Carlsbad, CA) for a more accurate quantification result. Approximately 1.5 ng of original genomic DNA molecules were used for library preparation. In the first step, transposons composed of a capture sequence and a transposase recognition sequence were inserted at a regular interval along the gDNA molecules. Next, these transposon-inserted DNA molecules were hybridized with barcode-labelled 3-μm diameter magnetic beads containing oligonucleotide sequences with a PCR primer annealing site, an stLFR barcode, and a sequence complementary to the capture sequence on the transposon. After hybridization, the barcode was transferred to the transposon-inserted DNA subfragments through a ligation step. The excess oligonucleotides and transposons were then digested with exonuclease and the transposase enzyme was denatured with sodium dodecyl sulfate. Next, the second adapter was introduced by a previously described 3′-branch ligation using T4 ligase [18]. Finally, PCR amplification was performed using primers annealing to the 5′ bead and 3′-branch adapter sequences. The PCR reaction was purified using Agencourt® AMPure XP beads (Beckman Coulter, Brea, CA) and quantified using the Qubit^TM^ dsDNA HS Assay Kit (Invitrogen, Carlsbad, CA). The PCR product fragment sizes were assessed using an Agilent High Sensitivity DNA Kit (Agilent, 5067-4626) on a Agilent 2100 Bioanalyzer. The average fragment size of the prepared stLFR library was 1,003 bp. A quantity of 20 ng of PCR product from the stLFR library was used to prepare DNA Nanoballs (DNBs) using the DNBSEQ-G400RS High Throughput stLFR Sequencing Set (MGI, Shenzhen, China) following the manufacturer’s protocol. The prepared DNB library was loaded onto 2 lanes of a DNBSEQ-G400RS flow cell (MGI, Shenzhen, China) and then sequenced on a DNBSEQ-G400RS (MGI, Shenzhen, China) using the DNBSEQ-G400RS stLFR sequencing set (MGI, Shenzhen, China). Library preparation and sequencing were performed at the BGI Australia Sequencing Facility (Clive Berghofer Cancer Research Centre, Herston, QLD) and BGI-Shenzhen (Shenzhen, China).

### Illumina sequencing

The Illumina library was prepared using the Nextera Flex DNA kit. The library was sequenced on an SP flow cell (14%) of the Illumina Nova Seq 6000 sequencing platform (Ramaciotti Centre, University of New South Wales, Australia) using the paired-end protocol to produce 112 million 150-bp reads in pairs, an estimated 43× genome coverage. The median insert size was 713 bp.

### Sequence read preparation

ONT read length and quality were calculated with NanoPlot v1.22 [19]. Long reads from PacBio and ONT were prepared using 2 or 3 alternative strategies, respectively:

- All: no filtering of reads
- Filtered: ONT long reads were adapter-trimmed using Porechop v0.2.4 (Porechop, RRID:SCR_016967) [20]. ONT and PacBio reads were filtered using Filtlong v0.2.0 [21] by removing 10% of the worst reads and reads shorter than 1 kb.
- Pass (ONT only): only the passed reads were used (average base-call quality score >7).

The PacBio subreads were randomly subsampled down to a 32× genome coverage using Rasusa v0.1.0 [22]. Raw Illumina and BGI short reads were adapter-trimmed using Trimmomatic v0.36 (Trimmomatic, RRID:SCR_011848) [23] (LEADING:3 TRAILING:3 SLIDINGWINDOW:4:15 ILLUMINACLIP:2:30:10 MINLEN:36). PolyG tail trimming was performed on the Illumina reads using fastp v0.20.0 (fastp, RRID:SCR_016962) [24].

### Genome size estimation

The *k*-mer counting using the trimmed Illumina and BGI reads was performed using Jellyfish v2.210 (Jellyfish, RRID:SCR_005491) [25], generating *k*-mer frequency distributions of 21-, 23-, and 25-mers. The histograms of the *k*-mer occurrences were processed by GenomeScope (GenomeScope, RRID:SCR_017014) [26], which estimated a genome haploid size of 653 and 616 Mb with ∼71% and 74% of unique content and a heterozygosity level of 0.65% and 0.77% from Illumina and BGI reads, respectively.

### Assembly of genomes

*De novo* assembly of ONT and PacBio reads was performed using Redbean v2.5 (WTDBG, RRID:SCR_017225) [27], Flye v2.5 (Flye, RRID:SCR_017016) [28], Canu v1.8 (ONT) or v1.9 (PacBio) (Canu, RRID:SCR_015880) [29], and Raven v1.1.6 [30] with default parameters. For Redbean, Flye, and Canu, the estimated genome size was set to 780 Mb [31]. For ONT data, 4 rounds of error correction were performed using Racon v1.4.9 (Racon, RRID:SCR_017642) [32] with recommended parameters (-m 8 -x -6 -g -8 -w 500) based on minimap2 v2.17-r943-dirty [33] overlaps, followed by 1 round of Medaka v0.8.1 [34] using the r941_prom_high model to create the consensus sequence. The resulting sequence was polished with Pilon v1.23 (Pilon, RRID:SCR_014731) [35] using the Illumina reads mapped with BWA-MEM v0.7.13 (BWA, RRID:SCR_010910) [36] and with the settings to fix bases (--fix bases). Polishing of the Medaka consensus sequence with Illumina reads was also performed by NextPolish v1.1.0 [37] with default settings (BWA for the mapping step). Hybrid assembly was generated with MaSuRCA v3.3.3 (MaSuRCA, RRID:SCR_010691) [38] using the Illumina and the ONT or PacBio reads and using Flye v2.5 to perform the final assembly of corrected megareads (parameter FLYE_ASSEMBLY=1). Diploid *de novo* genome assembly of PacBio reads was performed with FALCON v1.3.0 (FALCON, RRID:SCR_016089) [39] using a genome size of 780 Mb, a length cut-off of 40,740 bp, and a seed read coverage cut-off of 30. A total of 19 Gb of preassembled reads was generated (24× coverage). After assembly and haplotype separation by FALCON-Unzip v1.2.0 [39], polishing was performed as part of the FALCON-Unzip workflow. PacBio reads were mapped to the primary FALCON-Unzip assembly using minimap2 v2.17-r954-dirty [33]. A read coverage histogram was generated from this alignment using Purge Haplotigs v.1.1.0 [40] to obtain the read depth cut-off values (-l 17 -m 52 -h 190) required to identify redundant contigs. Illumina reads were assembled using SPAdes v3.13.1 (SPAdes, RRID:SCR_000131) [41].

Two lanes of stLFR reads for the same sample were demultiplexed using a subfunction of SuperPlus v1.0 [42] and combined for the downstream analysis. Adapter sequences were removed from read data using Cutadapt v2.4 (cutadapt, RRID:SCR_011841) [43] with the recommended parameters (--no-indels -O 10 --discard-trimmed -j 42). Read sequences were then converted to 10X Genomics format by BGI’s inhouse software, which contains 3 steps: (i) Change the format of reads’ head from MGI to Illumina. (ii) Change the quality number of “N” base from 33 (ASIC II code = !) to 35 (ASIC II code = #) to meet the 10X Genomics’ quality system. (iii) Merge 2 or more barcodes into 1 barcode randomly due to the limitation of barcode types for 10X Genomics. To meet the memory requirement of the assembler, the barcodes with <10 reads were removed from the dataset. *De novo* assembly was performed by Supernova v2.1.1 (Supernova assembler, RRID:SCR_016756) [44] using the suggested parameters (--maxreads=2100000000 --accept-extreme-coverage --nopreflight). TGS-GapCloser v1.0.0 (TGS-GapCloser, RRID:SCR_017633) [45,46] was used to fill the gaps between contigs within same scaffolds, and this process was performed under the use of error-corrected ONT or PacBio data by Canu. The number of gaps within scaffolds was computed using the formula: number of contigs − number of scaffolds.

The technical specifications of the computing clusters used in this study are provided in Supplementary Table S1. An estimation of computational costs based on Amazon EC2 on-demand pricing is provided in Supplementary Table S2.

### Assembly evaluation

Assembly statistics were computed using QUAST v5.0.2 (QUAST, RRID:SCR_001228) [47] with a minimum contig length of 10 kb and the parameters --fragmented --large. The publicly available reference genome of *M. integrifolia* v2 (Genbank accession: GCA_900631585.1) [8] was used as the reference genome for QUAST. To estimate the base accuracy, QUAST was used to compute the number of mismatches and indels as compared to the Illumina short-read assembly generated by SPAdes. The Illumina short-read assembly was generated using more accurate short reads as compared to long reads; therefore it contained fewer base errors. Consequently the number of mismatches and indels identified in the long-read assemblies as compared to the short-read assembly will reflect their base error rates. We noted that this would only enable comparison to X% of the genome because the Illumina-only assembly is relatively incomplete. Furthermore, the Illumina assembly would be expected to have errors and those errors would result in calling errors in other assemblies even when they are actually correct. To evaluate the completeness of the genome, the assemblies were subjected to BUSCO v3.0.2 (BUSCO, RRID:SCR_015008) [48] with the eudicotyledons_odb10 database (2,121 genes). The K-mer Analysis Toolkit v2.4.2 (KAT, RRID:SCR_016741) [49] comp and kat_distanalysis commands were used to estimate *k*-mer assembly completeness by reference to the Illumina or stLFR short reads.

## Results

### Illumina genome assembly

Illumina sequencing generated 112.5 million 150-bp paired-end reads, which correspond to ∼41× coverage of the genome. After adapter and polyG tail trimming, short reads were assembled using the SPAdes software. The resulting assembly consisted of 1,631,183 contigs totaling 864 Mb in length and contained 15,583 contigs >10 kb with a total length of 338 Mb (Supplementary Table S3). The assembly was highly fragmented, with a contig N50 of 23.9 kb. Genome completeness assessment using BUSCO revealed that the assembly contained 65% of complete BUSCOs (including 58% of single-copy genes), 18% of fragmented BUSCOs, and 17% of missing BUSCOs.

### ONT genome assembly

For the ONT sequencing, we combined the results of 1 PromethION and 1 MinION flow cell, generating a total of 24.9 Gb of data with a read length N50 of 27.8 kb (Table 1). The PromethION flow cell and the MinION flow cell generated 23.2 and 1.7 Gb of data, respectively, with a read length N50 of 28.5 and 16.6 kb and a median read quality of 6.3 and 8.9. The ONT reads were assembled using 4 different long-read assemblers (Redbean, Flye, Canu, Raven) and 3 different read subsets representing different genome coverage (21×, 28×, and 32×). The statistics for each assembly are presented in Supplementary Table S4 and Fig. S1. Canu and Flye generated the largest and most contiguous assemblies while Redbean produced the smallest and least contiguous assembly (∼750 Mb, contig N50 ∼700 kb) followed by Raven (∼770 Mb, contig N50 ∼1 Mb). Flye consistently produced assemblies of ∼812 Mb with a contig N50 of ∼1.5 Mb whereas Canu and Redbean assembly contiguity increased as the read coverage increased. In particular, the Canu contig N50 significantly increased from 706 kb (21×) to 1.43 Mb (32×). For 28× and 32× genome coverage, Raven assemblies were similar in size (Raven is the only tool that does not require an estimated genome size as a mandatory input parameter.) Raven was the only tool run on a GPU-accelerated server and it was the fastest assembler, followed by Redbean and Flye. Canu was ∼5 and 10 times slower than Flye and Redbean, respectively.

**Table 1:** Sequencing data

Parameter	ONT	PacBio	BGI	Illumina
No. of raw reads	3,129,385	3,170,206	738,145,698	112,508,072
No. of trimmed reads			611,835,983	109,046,265
No. of reads used in assembly	3,129,385	3,170,206	372,797,279	109,046,265
No. of bases	24,915,207,810	65,228,232,554	74,559,455,800	31,961,393,885
Read length N50 (bp)	27,842	35,866	2 × 100	2 × 150
Mean read length (bp)	7,962	20,575	2 × 100	2 × 150
Genome coverage (x)	32	84	96	41
Cost (USD)*	3,270	12,560	1,120	721
Sequencing date	March/April 2019	June 2019	May/June 2019	April 2019
DNA amount (ng)	1,200–1,500	15,000	10	500

*Australian dollar costs were converted to US dollars at an exchange rate of 0.685 USD/AUD. The ONT cost includes library preparation (400 USD) and sequencing on 1 PromethION flow cell (2,050 USD) and 1 MinION flow cell (820 USD). The PacBio cost includes library preparation (1,187 USD) and sequencing on 8 SMRT cells (11,373 USD). The stLFR cost is estimated on the basis of the number of raw reads subsequently used in assembly (∼90 Gb) and includes library preparation (400 USD) and sequencing (8 USD per Gb). Genome coverage estimates were computed on the basis of the number of reads used in assembly and an estimated genome size of 780 Mb.

We subsequently polished the Redbean, Flye, Canu, and Raven draft assemblies using the ONT long reads followed by the Illumina short reads. Long-read polishing was performed using the Racon and Medaka tools. Two software tools to fix base errors using short reads were compared: the widely used tool Pilon and the recently developed algorithm NextPolish. Those polishing steps greatly improved the genome completeness as indicated by the percentage of complete BUSCOs, which increased from 53% (Redbean), 70% (Canu), or 79% (Flye, Raven) to 85% (Redbean) or 89% (Flye, Raven, Canu) after long-read polishing and 92% (Redbean) or 95% (Flye, Raven, Canu) after long-read and short-read polishing (Supplementary Table S5 and Fig. S2). As an estimation of the base accuracy, we computed the number of mismatches and indels as compared to the Illumina short-read assembly generated by SPAdes (Supplementary Fig. S3 and Table S6). The Canu assembly was less accurate than the other assemblies (NextPolish: 582 vs 485–503 mismatches per 100 kb, 68 vs 42–49 indels per 100 kb; Pilon: 670 vs 529–593 mismatches per 100 kb, 108 vs 76–85 indels per 100 kb) and contained a higher percentage of duplicated genes (16–17% vs 12–14%).

The base accuracy metrics suggest that NextPolish performed slightly better than Pilon. In particular, the number of indels was greatly reduced after polishing with NextPolish as compared to Pilon (Flye: 48 vs 83 indels per 100 kb, Canu: 68 vs 108, Raven: 49 vs 85, Redbean: 42 vs 76, Supplementary Table S6). Pilon and NextPolish resulted in similar genome completeness when applied to the Canu and Raven assemblies. The genome completeness was slightly better after 2 iterations of NextPolish than after 2 iterations of Pilon for the Flye (95.4% vs 95.2%) and Redbean assemblies (91.9% vs 91.6%). A second iteration of Pilon resulted in a slight decrease in the number of missing genes and a higher accuracy for all 4 assemblers whereas a second iteration of NextPolish did not improve the genome completeness and accuracy (mismatches) for the Canu and Raven assemblies. Therefore, depending on the assembler and the polisher used, the number of recommended polishing iterations might be different.

Assembly completeness was also estimated by comparing the *k*-mer spectrum of the polished assemblies to the *k*-mer spectrum of the Illumina short reads (Supplementary Table S7 and Fig. S4). The *k*-mer analysis suggested that Flye produced the most complete polished assembly (99.0%) followed by Canu (97.9%) and Raven (97.4%) and finally Redbean (92.3%). The trends were similar when the *k*-mer analysis was performed using the stLFR short reads.

**Figure 1:**
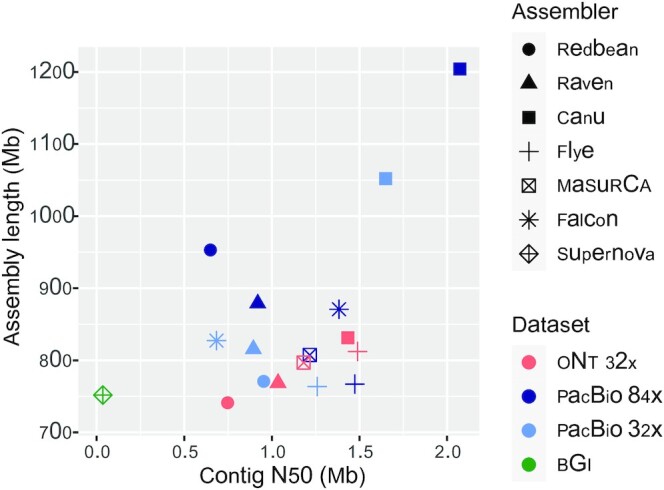
ONT, PacBio, and BGI genome assembly statistics. The total assembly length is plotted against the contig N50 for each assembler and sequencing dataset.

**Figure 2:**
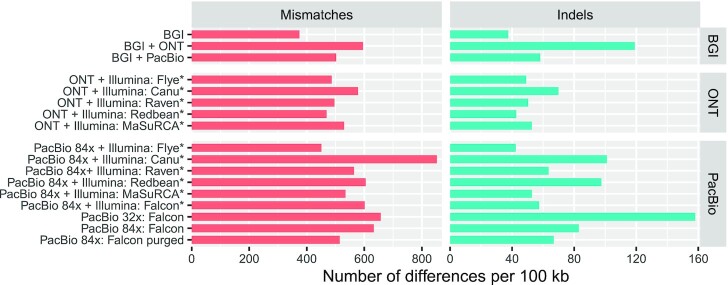
Number of mismatches and indels identified in the long-read assemblies as compared to the Illumina short-read assembly generated by SPAdes. The BGI + ONT and BGI + PacBio assemblies were polished with the BGI stLFR reads using 1 iteration of NextPolish. The ONT + Illumina assemblies (except MaSuRCA) were polished with the ONT long reads using Racon and Medaka followed by the Illumina short reads using 1 iteration of NextPolish. The PacBio + Illumina assemblies (except MaSuRCA) were polished with the Illumina short reads using 1 iteration of NextPolish. *Assembly polished using Illumina reads.

**Figure 3:**
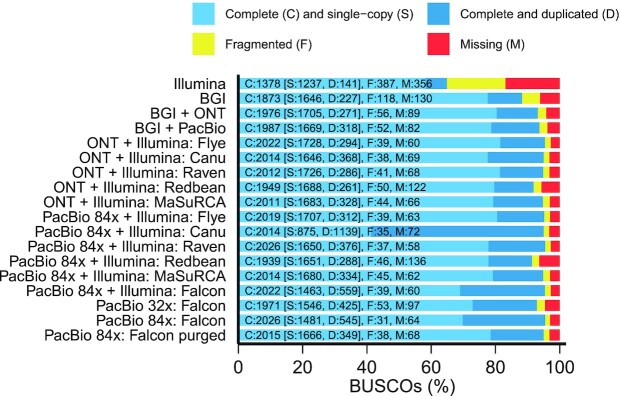
BUSCO analysis of assemblies using the eudicotyledons dataset (2,121 genes). The x-axis depicts the percentage of complete and single-copy, complete and duplicated, fragmented, and missing BUSCOs and the y-axis indicates the assembly assessed. The BGI + ONT and BGI + PacBio assemblies were polished with the BGI stLFR reads using 1 iteration of NextPolish. The ONT + Illumina assemblies (except MaSuRCA) were polished with the ONT long reads using Racon and Medaka followed by the Illumina short reads using 1 iteration of NextPolish. The PacBio + Illumina assemblies (except MaSuRCA) were polished with the Illumina short reads using 1 iteration of NextPolish.

As an alternative method to long-read–only assembly followed by polishing with short reads, a hybrid assembly was generated using MaSuRCA. The ONT + Illumina assembly showed a similar size (797 Mb), contiguity (contig N50 = 1.18 Mb), completeness (94.8% complete BUSCOs including 15.5% duplicated BUSCOs), and a slighlty lower accuracy (530 mismatches per 100 kb, 53 indels per 100 kb) as the Flye and Raven assemblies with subsequent polishing with Illumina reads (Figs 1–3 and Supplementary Fig. S2 and S3, Tables S4 and S5). Short-read polishing or long-read followed by short-read polishing did not significantly improve the genome completeness of the MaSuRCA assembly (Supplementary Table S5), which is expected as the super-reads constructed by this tool are based on the Illumina reads.

### PacBio genome assembly

With 8 single-molecular real-time cells in the PacBio Sequel platform, we generated 3,170,206 subreads with a read length N50 of 35.9 kb and representing a total of 65.2 Gb (Table 1). The data correspond to ∼84× coverage of the estimated 780 Mb genome size. The assembly of the PacBio data was conducted using the same tools used for the ONT data: the 4 long-read assemblers Redbean, Flye, Canu, and Raven and the hybrid assembler MaSuRCA (Supplementary Table S8). The PacBio assemblies showed a similar contiguity as the ONT assemblies (except Canu) and were larger (except Flye) (Fig. 1). Before polishing, their genome completeness was higher than the ONT assemblies, indicating a higher accuracy of PacBio reads (Supplementary Fig. S2). The Redbean assembly was the most fragmented (contig N50 = 649 kb) and the least complete (89% complete BUSCOs). The Flye assembly was highly contiguous (contig N50 = 1.47 Mb) and the smallest in size (767 Mb). The Raven assembly (879 Mb) consisted of the fewest contigs (n = 1,730) with a contig N50 of 919 kb. The Canu assembly was the largest (1.2 Gb) but it contained a high fraction of duplication as reported by QUAST (1.64) and confirmed by the percentage of duplicated BUSCOs (53%) and the *k*-mer spectra (Supplementary Fig. S4). Therefore, the Canu assembly likely contains uncollapsed haplotypes corresponding to artefactually duplicated regions, as reported recently [50]. Aligning the PacBio assemblies to the *M. integrifolia* assembly identified a higher number of misassemblies in the Canu asssembly (n = 38,800) as compared to the other assemblies (n = 21,000–27,000). The PacBio + Illumina hybrid assembly (807 Mb, contig N50 = 1.22 Mb) contained 94.9% of complete BUSCOs including 16% of duplicated genes (Fig. 3).

To generate a phased diploid assembly, PacBio assembly was next performed using the FALCON assembler, followed by haplotype resolution and polishing using FALCON-Unzip. The resulting primary assembly consisted of 1,333 contigs totaling 871 Mb in length, with half of the assembly in contigs of 1.38 Mb or longer (Fig. 1). FALCON-Unzip also generated 2,488 alternate haplotigs spanning 495 Mb (i.e., 57% of the genome was haplotype resolved), with a contig N50 of 333 kb. BUSCO analysis on primary contigs showed ∼26% of duplicated genes, suggesting the presence of homologous primary contigs (Fig. 3). The Purge Haplotigs pipeline identified 569 primary contigs representing 112 Mb as likely alternate haplotypes (Supplementary Table S9). These contigs were transferred to the haplotigs set. The curated primary haploid assembly consisted of 762 contigs totaling 758 Mb with a contig N50 of 1.59 Mb and contained fewer duplicated genes (16%) with minimal impact on genome completeness (95% complete BUSCOs).

We subsequently polished the PacBio assemblies using the Illumina short reads. As expected, a reduced number of mismatches and indels was identified in the assemblies as compared to the Illumina assembly (Supplementary Fig. S3 and Table S6). Polishing decreases the number of missing BUSCOs but increased the number of duplicated BUSCOs for the Redbean, Flye, and Raven assemblies (Supplementary Table S10). Long-read followed by short-read polishing resulted in an increased percentage of single-copy BUSCOs and a reduced percentage of duplicated BUSCOs for the Canu assembly and, to a lesser extent, the Falcon assembly. Interestingly, the long-read polishing step did not improve the completeness of the Redbean, Flye, and Raven assemblies and similar or slightly better results were obtained after short-read polishing alone. Therefore, the recommended polishing strategy for PacBio assemblies might depend on the assembler used.

Using a quality-filtered subset of the subreads (equivalent to ∼67× genome coverage) led to a similar (Flye and Raven) or slightly higher (Redbean) assembly contiguity without affecting the genome completeness (only Redbean, Raven, and Flye were tested owing to the high computational requirements of Canu and Falcon) (Supplementary Fig. S1 and Table S8). Finally, to compare PacBio and ONT technologies, we randomly subsampled the PacBio subreads down to a coverage equivalent to the ONT data (32×). The resulting Flye assembly showed a similar size of 764 Mb, a lower contiguity (contig N50 = 1.26 Mb), and a similar genome completeness (94.7% complete BUSCOs) as the 84× coverage assembly (Fig. 1, Supplementary S2 and Table S8). The other 4 assemblers resulted in a reduced genome size and a slightly lower genome completeness. The decrease in coverage did not affect the Raven assembly contiguity (contig N50 = 894 kb). The Falcon assembly was the most affected by the decrease in coverage, with a decrease in the contig N50 from 1.38 Mb to 684 kb. Conversely, the Redbean assembly contiguity increased from 649 to 953 kb. The percentage of duplicated BUSCOs decreased for all the assemblies but remained high for the Canu (33%) and Falcon (20%) assemblies.

### stLFR genome assembly

stLFR generated 738 million 100-bp paired-end reads. To meet the requirements of the assembler, the barcodes with < 10 reads were removed, which resulted in 373 million reads representing 74.6 Gb of data and corresponding to ∼96× coverage of the genome (Table 1). stLFR reads were assembled using Supernova2 into an assembly of 40,789 scaffolds totaling 880 Mb in length (Table S11). A total of 5,065 scaffolds were larger than 10 kb, with a total length of 752 Mb and N50 of 3.54 Mb for scaffold and 35.6 kb for contig (Table 2). When compared to the Illumina short-read assembly, the stLFR assembly contained the fewest mismatches and indels (Fig. 2). Conserved BUSCO gene analysis revealed that the stLFR assembly contained 88.3% of complete genes from the eudicotyledons dataset (Fig. 3).

**Table 2:** Gap filling for stLFR assembly using error-corrected ONT or PacBio reads

Parameter	Supernova	ONT		PacBio	
		After gap filling	Improvement (%)	After gap filling	Improvement (%)
No. of input long reads		1,056,095		674,796	
Useable reads for filling (%)		1.74		2.95	
No. of scaffolds	5,065	5,332	5.3	5,446	7.5
Scaffold N50 (bp)	3,540,919	3,523,921	−0.5	3,504,721	−1.0
Scaffold length (bp)	751,745,340	766,968,089	2.0	768,468,395	2.2
Largest scaffold size (bp)	30,143,475	31,148,326	3.3	31,237,530	3.6
No. of contigs	19,954	6,022	−70	5,717	−71
Contig N50 (bp)	35,605	1,046,570	2,839	1,598,608	4,390
Contig length (bp)	594,029,544	742,770,175	25	758,126,937	28
Largest contig size (bp)	517,998	9,683,794	1,769	23,824,472	4,499
No. of gaps within scaffolds	14,889	690	−95	271	−98
No. of Ns per 100 kb	16,934	3,042	−82	1,290	−92
No. of complete BUSCOs (%)					
All	1,873 (88.3)	1,963 (92.5)	4.8	1,983 (93.5)	5.8
Single-copy	1,646 (77.6)	1,710 (80.6)	3	1,679 (79.2)	1.6
Duplicated	227 (10.7)	253 (11.9)	1.2	304 (14.3)	3.6

QUAST analysis was performed using a minimum contig size of 10 kb and the parameters --fragmented --large --split-scaffolds.

Inclusion of ONT or PacBio data to fill the gaps within scaffolds led to a 29- or 45-fold increase in the contig N50 length from 35.6 kb to 1.05 or 1.60 Mb and a 22- or 55-fold decrease in the number of gaps within scaffolds larger than 10 kb from 14,889 to 690 or 271 (Table 2). The scaffold N50 slightly decreased by 0.02 or 0.04 Mb due to the adjustment of the estimated gaps. For both gap-filled assemblies, the total assembly length increased correspondingly to ∼895 and 770 Mb for scaffolds larger than 10 kb. The largest contig size increased from 518 kb to 9.7 Mb (ONT) and 23.8 Mb (PacBio). In addition, the genome completeness was improved in the gap-filled assemblies, with BUSCO detecting 4.8% (ONT) and 5.8% (PacBio) more complete genes. The number of complete duplicated BUSCOs was slightly lower in the ONT filled assembly (11.9%) than in the PacBio filled assembly (14.3%). Finally, the estimated*k*-mer assembly completeness increased in the gap-filled assemblies from 95.8% to 96.7% (ONT) and 97.4% (PacBio) (Supplementary Table S7). Further polishing of gap-filled assemblies using the stLFR reads resulted in a slight increase in the genome completeness to 93.2% (ONT) and 93.7% (PacBio) of complete BUSCO genes (Supplementary Table S11 and Fig. S2) and a decrease in the number of indels (Supplementary Table S6 and Fig. S3).

## Discussion

We report a comparison of 3 long-read sequencing datasets generated from the same plant DNA sample. *M. jansenii* was selected for this study because of its significance in conservation and breeding. All 4 species of *Macadamia* are listed as threatened under Australian legislation, but *M. jansenii* is particularly vulnerable because it has been recorded at only 1 location. *M. jansenii* has not been domesticated, and its small and bitter nuts are obstacles that restrict simple introgression in breeding. However, the characteristic small tree size, being 50% smaller than commercial cultivars, is of interest for use in high-density orchard design and it is being trialled as a rootstock for this purpose [51]. It is the most northern *Macadamia* species and may be a source of genes for adaptation to warmer climates [52]. Hybrids of *M. integrifolia* and *M. jansenii* have been produced.

The 3 long-read sequencing technologies significantly improved the assembly completeness as compared to the assembly produced using the Illumina reads only (65% of complete BUSCOs). The cost of generating 1 Gb of sequencing data (including the library preparation) was 193 USD for PacBio Sequel I, 97 USD for ONT PromethION, and 12 USD for BGI stLFR (raw reads subsequently used in assembly). Virtual long reads were generated using the stLFR protocol. This technology benefits from the accuracy and the low cost of a short-read sequencing platform while providing long-range information. stLFR was the cheapest approach, and it generated an assembly with the fewest single base and indel errors. Furthermore, the assembly generated by Supernova was phased. That said, the stLFR assembly was more fragmented than the other long-read technologies. We also demonstrated that stLFR could be used as a complementary technology to ONT. Indeed, the inclusion of Nanopore reads significantly increased the stlFR assembly contiguity, with N50 reaching 1 Mb, and improved the genome completeness. Interestingly, the gap-filling step only used 1.7% of the ONT reads, suggesting that a real-time selective sequencing approach could be used to select specific molecules that would be informative for filling the gaps [53].

When all the reads were incorporated, the assemblies generated using the PacBio and ONT data were comparable in terms of assembly contiguity (contig N50 of ∼1.5 Mb) and genome completeness (95% of complete BUSCOs). However, when we utilized the same amount of data for each platform (32× coverage), the contiguity of the PacBio assembly produced by Falcon was halved and became only half the size of the ones from the ONT Flye or Canu assemblies. The Flye and Raven assemblers proved to be more robust to the PacBio coverage drop as the assembly contig N50 only decreased from 1.47 to 1.26 Mb (Flye) and from 919 to 894 kb (Raven). Additionally, we found that polishing the ONT assembly with the Illumina short reads was required to reach a similar genome completeness to that of the PacBio assembly. For both ONT and PacBio data, the highest contiguity was obtained with a long-read polished assembly as compared to a hybrid assembly incorporating both the short and long reads.

Since the sequence data were generated, the PacBio SMRT platform has transitioned from the Sequel I to the Sequel II instrument, with an 8-fold increase in the data yield. The latest platform produces high-fidelity reads that are more accurate than the continuous long reads assembled in this study. Consequently the cost to generate a similar PacBio assembly on the Sequel II system will be dramatically reduced and the assembly quality is likely to improve while requiring fewer computational resources.

The DNA material requirements to prepare the sequencing library are another important parameter to consider when choosing a sequencing technology. For ONT sequencing, it is recommended to obtain ≥1–2 μg of high molecular weight DNA. The stLFR library construction requires ≥10 ng of high molecular weight DNA. PacBio SMRT sequencing has a high genomic DNA input requirement of 5–20 μg of high molecular weight DNA for standard library protocol depending on the genome size but the PacBio low DNA input protocol has reduced this requirement to as low as 100 ng per 1 Gb genome size [54]. Furthermore, PacBio recently released an amplification-based ultra-low DNA input protocol starting with 5 ng of high molecular weight DNA.

The computational requirements and associated cost should be considered and will largely depend on the genome size of the species of interest. There were important differences in the assembly run time and memory usage depending on the tool used. For instance, short-read polishing using NextPolish used less memory than Pilon while providing similar results. GPU-accelerated computing greatly reduced the computing time for some tools such as Racon, Medaka, or Raven. There are also challenges associated with the rapid evolution of technologies and software. For example we observed a significant improvement in the ONT assembly contiguity depending on the basecaller or assembler version used. The newest releases of assemblers such as Canu v2.1, Flye v2.8, or Raven v1.1.10 will likely generate improved assemblies.

The 3 long-read technologies produced highly contiguous and complete genome assemblies. Next, long-range scaffolding approaches such as chromosome conformation capture (Hi-C, Chicago) or physical maps technologies (optical map, restriction map) are required to order and orient the assembled contigs into chromosome-length scaffolds [55].

## Data Availability

BGI, PacBio, ONT, and Illumina sequencing data generated in this study have been deposited in the SRA under BioProject PRJNA609013 and BioSample SAMN14217788. Accession numbers are as follows: BGI (SRR11191908), PacBio (SRR11191909), ONT PromethION (SRR11191910), ONT MinION (SRR11191911), and Illumina (SRR11191912). Assemblies and other supporting data are available from the *GigaScience* GigaDB repository [56].

## Additional Files

**Table S1:** Technical specifications of computing clusters

**Table S2:** Estimation of computational costs based on Amazon EC2 on-demand pricing as of 19 September 2020

**Table S3:** Illumina genome assembly statistics using SPAdes assembler

**Table S4:** ONT genome assembly statistics using Redbean, Flye, Canu, Raven, and MaSuRCA assemblers

**Table S5:** BUSCO genome completeness assessment of ONT long-read assemblies (Redbean, Flye, Canu, Raven) and hybrid assembly (MaSuRCA)

**Table S6:** QUAST assembly statistics using the Illumina short-read assembly as the reference genome

**Table S7:**  *k*-mer completeness of ONT, PacBio, and stLFR assemblies

**Table S8:** PacBio genome assembly statistics using Redbean, Flye, Falcon, Canu, Raven, and MaSuRCA assemblers

**Table S9:** PacBio genome assembly statistics and genome completeness assessment before and after Purge Haplotigs

**Table S10:** BUSCO genome completeness assessment of PacBio long-read assemblies (Redbean, Flye, Falcon, Canu, Raven) and hybrid assembly (MaSuRCA)

**Table S11:** BGI stLFR genome assembly statistics using Supernova assembler and TGS-GapCloser gap-closing software

**Figure S1:** Genome assembly statistics. The total assembly length is plotted against the contig N50 for each assembler and sequencing coverage. (A) ONT assemblies, (B) PacBio assemblies.

**Figure S2:** BUSCO genome completeness assessment. (A) ONT assemblies before and after Illumina short-read polishing using 1 iteration of NextPolish (Flye, Canu, Raven, Redbean) and MaSuRCA hybrid assembly, (B) PacBio assemblies using 32× or 84× sequencing coverage, (C) BGI stLFR assemblies before and after gap filling using ONT or PacBio data and after polishing using stLFR reads and 1 iteration of NextPolish.

**Figure S3:** Number of mismatches and indels identified in the long-read assemblies as compared to the Illumina short-read assembly generated by SPAdes. (A) ONT assemblies before and after Illumina short-read polishing using 1 iteration of NextPolish (Flye, Canu, Raven, Redbean) and MaSuRCA hybrid assembly; (B) PacBio assemblies before and after Illumina short-read polishing using 1 iteration of NextPolish (Falcon, Flye, Canu, Raven, Redbean) and MaSuRCA hybrid assembly; (C) BGI stLFR assemblies before and after gap filling using ONT or PacBio data and after polishing with stLFR reads using 1 iteration of NextPolish.

**Figure S4:**  *k*-mer spectra plots from the *k*-mer Analysis Toolkit comparing the *k*-mers found in Illumina reads to the *k*-mers found in ONT, PacBio, stLFR, and Illumina assemblies.

## Abbreviations

AUD: Australian dollars; bp: base pairs; BGI: Beijing Genomics Institute; BUSCO: Benchmarking Universal Single-Copy Orthologs; BWA: Burrows-Wheeler Aligner; DNB: DNA Nanoballs; dsDNA: double-stranded DNA; Gb: gigabase pairs; gDNA: genomic DNA; GPU: graphics processing unit; kb: kilobase pairs; Mb: megabase pairs; ONT: Oxford Nanopore Technologies; PacBio: Pacific Biosciences; QUAST: QUality ASsessment Tool; SMRT: single-molecule real-time; SPAdes: St. Petersburg genome assembler; SRA: Sequence Read Archive; SRE: Short Read Eliminator; stLFR: single-tube long fragment reads; SQB: sequencing buffer; USD: United States Dollar.

## Competing Interests

Employees of BGI (W.T., I.H., Q.Y., B.Y., O.W., M.X, P.W.), MGI (H.W.), and Complete Genomics (E.A., Q.M., R.D., B.A.P.) have stock holdings in BGI. The authors declare that they have no other competing interests.

## Funding

This work was funded by the Genome Innovation Hub, Office of Research Infrastructure, The University of Queensland. This work was supported in part by the Shenzhen Peacock Plan (NO.KQTD20150330171505310). L.J.M.C. was supported by a Discovery Project with grant number DP170102626 awarded by the Australian Research Council.

## Authors' Contributions

A.F. prepared the sample. B.T. supervised plant collection. S.K.R. performed ONT library preparation and sequencing. T.J.C.B. performed PacBio library preparation and sequencing. V.M. performed Illumina, ONT and PacBio assemblies and assembly evaluation. Q.Y. and H.W. performed stLFR library preparation and sequencing. I.H. supervised and reviewed stLFR library preparation and sequencing. W.T. performed stLFR assembly, gap filling, and statistics for stLFR. E.A., Q.M., R.D., O.W., and B.A.P. designed stLFR experiments and performed stLFR analyses. M.X. and P.W. supported stLFR analyses. B.Y. reviewed the manuscript. V.M. wrote the manuscript with input from all authors. R.J.H. and L.J.M.C. designed and supervised the project.

## Supplementary Material

giaa146_GIGA-D-20-00077_Original_Submission

giaa146_GIGA-D-20-00077_Revision_1

giaa146_GIGA-D-20-00077_Revision_2

giaa146_GIGA-D-20-00077_Revision_3

giaa146_Response_to_Reviewer_Comments_Original_Submission

giaa146_Response_to_Reviewer_Comments_Revision_1

giaa146_Response_to_Reviewer_Comments_Revision_2

giaa146_Reviewer_1_Report_Original_Submission CÃ©cile Monat, Ph.D. -- 4/20/2020 Reviewed

giaa146_Reviewer_1_Report_Revision_1 CÃ©cile Monat, Ph.D. -- 7/23/2020 Reviewed

giaa146_Reviewer_2_Report_Original_Submission Mile Å ikiÄ‡ -- 5/2/2020 Reviewed

giaa146_Reviewer_2_Report_Revision_1 Mile Å ikiÄ‡ -- 7/26/2020 Reviewed

giaa146_Reviewer_2_Report_Revision_2 Mile Å ikiÄ‡ -- 10/1/2020 Reviewed

giaa146_Supplemental_Files
