# Supplementary material for: Comparison of long-read methods for sequencing and assembly of a plant genome
Source: Gigascience. 2020 Dec 21;9(12):giaa146. doi: 10.1093/gigascience/giaa146 (PMC7751402; doi:10.1093/gigascience/giaa146)
Supplement: giaa146_GIGA-D-20-00077_Revision_2 [file giaa146_giga-d-20-00077_revision_2.pdf]

GigaScience

Comparison of long read methods for sequencing and assembly of a plant genome

--Manuscript Draft--

**Manuscript Number:**	GIGA-D-20-00077R2
**Full Title:**	Comparison of long read methods for sequencing and assembly of a plant genome
**Article Type:**	Research
**Funding Information:**	Shenzhen Peacock Plan (KQTD20150330171505310) — Not applicable<br>Genome Innovation Hub, Office of Research Infrastructure, The University of Queensland — Not applicable
**Abstract:**	Background:<br><br>Sequencing technologies have advanced to the point where it is possible to generate high accuracy, haplotype resolved, chromosome scale assemblies. Several long read sequencing technologies are available on the market and a growing number of algorithms have been developed over the last years to assemble the reads generated by those technologies. When starting a new genome project, it is therefore challenging to select the most cost-effective sequencing technology as well as the most appropriate software for assembly and polishing. For this reason, it is important to benchmark different approaches applied to the same sample.<br><br>Results:<br><br>Here, we report a comparison of three long read sequencing technologies applied to the de novo assembly of a plant genome, Macadamia jansenii . We have generated sequencing data using Pacific Biosciences (Sequel I), Oxford Nanopore Technologies (PromethION) and BGI (single-tube Long Fragment Read) technologies for the same sample. Several assemblers were benchmarked in the assembly of PacBio and Nanopore reads. Results obtained from combining long read technologies or short read and long read technologies are also presented. The assemblies were compared for contiguity, accuracy and completeness as well as sequencing costs and DNA material requirements.<br><br>Conclusions:<br><br>Overall, the three long read technologies produced highly contiguous and complete genome assemblies of Macadamia jansenii. At the time of sequencing, the cost associated with each method was significantly different but continuous improvements in technologies have resulted in greater accuracy, increased throughput and reduced costs. We propose updating this comparison regularly with reports on significant iterations of the sequencing technologies.
**Corresponding Author:**	Valentine Murigneux, M.Sc.<br>University of Queensland<br>Brisbane, QLD AUSTRALIA
**Corresponding Author Secondary Information:**	
**Corresponding Author's Institution:**	University of Queensland
**Corresponding Author's Secondary Institution:**	
**First Author:**	Valentine Murigneux
**First Author Secondary Information:**	
**Order of Authors:**	Valentine Murigneux<br>Subash Kumar Rai

Powered by Editorial Manager® and ProduXion Manager® from Aries Systems Corporation

	Agnelo Furtado
	Timothy J.C. Bruxner
	Wei Tian
	Qianyu Ye
	Hanmin Wei
	Bicheng Yang
	Ivon Harliwong
	Ellis Anderson
	Qing Mao
	Radoje Drmanac
	Ou Wang
	Brock A Peters
	Mengyang Xu
	Pei Wu
	Bruce Topp
	Lachlan J.M. Coin
	Robert J. Henry
**Order of Authors Secondary Information:**	
**Response to Reviewers:**	GIGA-D-20-00077<br>Comparison of long read methods for sequencing and assembly of a plant genome<br><br>We would like to thank the editorial staff and the reviewers for their time and valuable comments. The revised manuscript includes corrections, updated figures and additional data to address the comments raised by the reviewer #2. The supplementary table S11 reports the estimated computational costs to generate the assemblies. We believe our manuscript has improved through the corrections and the additional information provided. We have included a point-by-point replies to the reviewer's comments below, highlighted in purple. In the revised manuscript (Murigneux_et_al_revision2.pdf), the modifications are highlighted in yellow.<br><br>Reviewer reports:<br><br>Reviewer #1: I would like to thank the authors for their responses clarifying my interrogation and for their corrections and improvements to the manuscript.<br>I believe the manuscript has much improved through the clarifications and newly added data to complete the analysis.<br>I do not have any other comments on this work, and I would accept it to be published.<br>We thank the reviewer for this positive feedback.<br><br>Reviewer #2: The authors have answered most of the comments from my previous review adequately. My remaining concern is mostly related to the following paragraph:<br>"The cost of generating 1 Gb of sequencing data (including the library preparation) was 193 USD for PacBio Sequel I, 97 USD for ONT PromethION, and 12 USD for BGI stLFR (raw reads subsequently used in assembly). Virtual long reads were generated using the stLFR protocol. This technology benefits from the accuracy and the low cost of a short-read sequencing platform while providing long-range information. It was the cheapest and most accurate approach as it generated an assembly with the fewest single base and indel errors."<br><br>The claim of "the cheapest and most accurate approach" is very strong and it is not supported by the presented results.<br>The reasons for my concerns are the following:

Powered by Editorial Manager® and ProduXion Manager® from Aries Systems Corporation

	1. From the presented data, it is clear that BGI stLFR technology achieves an assembly with fewer single base and indel errors in comparison with an assembly generated from Illumina reads. However, this is just one of the measures and I deem that using just this one is not enough to support the claim that this is the most accurate approach. Furthermore, using the same reasoning, one might conclude that Illumina technology is the cheapest and most accurate.<br><br>We thank the reviewer for this comment. The main text has been reworded:<br>•In the 'stLFR genome assembly' section, the sentence "The stLFR assembly was the most accurate with the lowest number of mismatches and indels identified as compared to the Illumina short-read assembly" has been modified to "When compared to the Illumina short-read assembly, the stLFR assembly contained the lowest number of mismatches and indels".<br>•In the discussion, the sentence "It was the cheapest and most accurate approach as it generated an assembly with the fewest single base and indel errors." has been modified to "It was the cheapest approach and it generated an assembly with the fewest single base and indel errors".<br><br>2. Assessing the quality of de novo reconstructed genome is not straightforward. However, there are standard measures such as contiguity, the calculation of duplication rate, k-mer spectra. BGI assemblies without gap filling with ONT or PacBio reads are highly fragmented. Better than Illumina, but not comparable with other technologies. Once a new genome is assembled, the subsequent essential analysis is annotation. If one looks from this perspective, an important measure of the quality is BUSCO score. Using PacBio data only, most assemblers achieve better BUSCO scores than Supernova with BGI stLFR reads. Using PacBio + Illumina and ONT + Illumina also lead to better BUSCO scores. It is important to emphasize that the assembly produced using Illumina reads only has the worst BUSCO score.<br><br>The following sentence has been added to the discussion to emphasize that the assembly produced using Illumina reads only has the worst BUSCO score: "The three long-read sequencing technologies significantly improved the assembly completeness as compared to the assembly produced using the Illumina reads only (65% of complete BUSCOs)."<br><br>3. Figures and tables in the main document are one of the most important parts of a manuscript because it is easy to memorize them. Figures 1, 2, and 3 are essential for understanding the assembly quality. Yet, results achieved with BGI technology are not presented in Figure 1. Furthermore, it is not clear why authors take only Flye for ONT reads, and Falcon for PacBio reads to comparison regarding BUSCO score and indels and mismatches count. It might be better if they took more assemblers for each of the read sets. Differences for ONT reads are tiny. Flye has slightly better BUSCO results for ONT and ONT + Illumina than Canu and Raven but also has somewhat worse results for indels and mismatches in comparison to Redbean and Raven. The need for more presented tools is even more evident for PacBio reads. Although Flye achieves slightly worse BUSCO score than Falcon, it is by far best tool for PacBio data measuring indels and mismatches, and produced results are very near to results achieved by BGI stLFR technology.<br><br>We agree with the reviewer regarding the suggestion to include all the tools benchmarked in the main figures and tables.<br>•Figure 1 has been modified to include the results of the BGI assembly.<br>•Figures 2 and 3 have been modified to include all the assemblers benchmarked.<br>•The Table 2 has been removed as it was only showing some of the assemblies generated. The text now referred the reader to the supplementary tables containing the results for all the assemblers tested (Table S2 and S4).<br><br>4. It is evident that BGI stLFR technology is cheaper than PacBio and ONT regarding the sequencing cost, but the total cost of the assembly should include the computational cost. The computational cost is often unjustly neglected, although it could be substantial and sometimes even higher than sequencing cost. In my previous review, I emphasized the need for the inclusion of the computational cost in the total cost. A significant number of researchers perform their analysis in the cloud, and they would be interested in having an estimation of this cost. It should include all

Powered by Editorial Manager® and ProduXion Manager® from Aries Systems Corporation

	computationally intensive tasks from basecalling, production of consensus reads, assembly, and polishing. Since authors have already measured the performance (CPU hours and memory), the cost might be easily calculated comparing available machines and clusters with the servers of similar performances in the cloud.<br><br>We agree with the reviewer that the computational cost is an important parameter that should be included in the total cost to generate a genome assembly. We used the Amazon EC2 On-Demand pricing online resources to compute estimated cost for each technology by comparing to the available servers of similar performances in the cloud.<br>•Table S11 provides an estimation of the cost to generate the final polished assembly per technology including the assembly, polishing and gap filling steps. We did not include the ONT basecalling step as this task was performed directly on the PromethION machine. Similarly, the generation of subreads was performed on the PacBio Sequel instrument by the sequencing facility.<br>•Estimated cost to run each assembler (SPAdes, Redbean, Flye, Raven, MaSuRCA) is provided in the Tables S1, S2 and S4.<br><br>Minor comments.<br>In supplementary files:<br>- labels for specific assemblers are non-consistent. It could mislead the reader<br>We thank the reviewer for this comment. The labels for the assemblers in the Figure S1 are now consistent between the panels A and B.<br><br>- x-scales of the figures are different (e.g., Figure 2). Therefore it is difficult to compare results<br>The x-scales in the Figures S1 and S2 are now similar between the different panels.<br><br>The authors have made a great analysis of available technologies, and I argue that there is a need for such a comprehensive analysis for a plant genome. If they provide the required data and, in their conclusion, precisely compare different technologies supported by data, I will support the publication of this manuscript.
**Additional Information:**	
**Question**	**Response**
Are you submitting this manuscript to a special series or article collection?	No
**Experimental design and statistics**<br><br>Full details of the experimental design and statistical methods used should be given in the Methods section, as detailed in our Minimum Standards Reporting Checklist. Information essential to interpreting the data presented should be made available in the figure legends.<br><br>Have you included all the information requested in your manuscript?	Yes
**Resources**<br><br>A description of all resources used, including antibodies, cell lines, animals and software tools, with enough	Yes

information to allow them to be uniquely identified, should be included in the Methods section. Authors are strongly encouraged to cite Research Resource Identifiers (RRIDs) for antibodies, model organisms and tools, where possible.<br><br>Have you included the information requested as detailed in our Minimum Standards Reporting Checklist?	
**Availability of data and materials**<br><br>All datasets and code on which the conclusions of the paper rely must be either included in your submission or deposited in publicly available repositories (where available and ethically appropriate), referencing such data using a unique identifier in the references and in the "Availability of Data and Materials" section of your manuscript.<br><br>Have you have met the above requirement as detailed in our Minimum Standards Reporting Checklist?	Yes

Manuscript

Click here to access/download;Manuscript;Murigneux_et_al_revision2.pdf

OXFORD (GIGA)^n SCIENCE

GigaScience, 2017, 1–11

doi: xx.xxxx/xxxx
Manuscript in Preparation
Paper

PAPER

Comparison of long read methods for sequencing and assembly of a plant genome

Valentine Murigneux[1,3,*], Subash Kumar Rai[1,3], Agnelo Furtado[2], Timothy J.C. Bruxner[3], Wei Tian[4,5], Qianyu Ye[4,5], Hanmin Wei[4,6], Bicheng Yang[4,5], Ivon Harliwong[4,5], Ellis Anderson[6,7], Qing Mao[6,7], Radoje Drmanac[4,6,7], Ou Wang[4], Brock A. Peters[4,6,7], Mengyang Xu[4,8], Pei Wu[4,9], Bruce Topp[2], Lachlan J.M. Coin[1,3,10] and Robert J. Henry[2]

[1]Genome Innovation Hub, The University of Queensland, 306 Carmody Road, St Lucia, Brisbane, QLD 4072 Brisbane, Australia and [2]Queensland Alliance for Agriculture and Food Innovation, The University of Queensland, St Lucia, QLD 4072, Australia and [3]Institute for Molecular Bioscience, The University of Queensland, 306 Carmody Road, St Lucia, QLD 4072 Brisbane, Australia and [4]BGI-Shenzhen, Shenzhen 518083, China and [5]BGI-Australia, 300 Herston Road, Herston QLD 4006, Australia and [6]MGI, BGI-Shenzhen, Shenzhen 518083, China and [7]Advanced Genomics Technology Lab, Complete Genomics Inc., 2904 Orchard Parkway, San Jose, California 95134, USA and [8]BGI-Qingdao, Qingdao, 266555, China and [9]BGI-Tianjin, Tianjin, China and [10]The Peter Doherty Institute for Infection and Immunity, The University of Melbourne, 792 Elizabeth Street, Melbourne, Victoria, 3000, Australia

[*]Correspondence address. Valentine Murigneux, Genome Innovation Hub, University of Queensland, St Lucia, Brisbane, QLD 4072, Australia. E-mail: v.murigneux@uq.edu.au

Abstract

Background: Sequencing technologies have advanced to the point where it is possible to generate high accuracy, haplotype resolved, chromosome scale assemblies. Several long read sequencing technologies are available on the market and a growing number of algorithms have been developed over the last years to assemble the reads generated by those technologies. When starting a new genome project, it is therefore challenging to select the most cost-effective sequencing technology as well as the most appropriate software for assembly and polishing. For this reason, it is important to benchmark different approaches applied to the same sample.
Results: Here, we report a comparison of three long read sequencing technologies applied to the de novo assembly of a plant genome, *Macadamia jansenii*. We have generated sequencing data using Pacific Biosciences (Sequel I), Oxford Nanopore Technologies (PromethION) and BGI (single-tube Long Fragment Read) technologies for the same sample. Several assemblers were benchmarked in the assembly of PacBio and Nanopore reads. Results obtained from combining long read technologies or short read and long read technologies are also presented. The assemblies were compared for contiguity, base accuracy and completeness as well as sequencing costs and DNA material requirements.
Conclusions: Overall, the three long read technologies produced highly contiguous and complete genome assemblies of *Macadamia jansenii*. At the time of sequencing, the cost associated with each method was significantly different but continuous improvements in technologies have resulted in greater accuracy, increased throughput and reduced costs. We propose updating this comparison regularly with reports on significant iterations of the sequencing technologies.

Key words: Assembly; long reads; PacBio; Pacific Biosciences; Sequel; Oxford Nanopore Technologies; PromethION; BGI; single-tube long fragment read; stLFR

Introduction

Advances in DNA sequencing enable the rapid analysis of genomes driving biological discovery. Sequencing of complex genomes, that are very large and have a high content of repetitive sequences or many copies of similar sequences remains challenging. Many plant genomes are complex and the quality of published sequences remains relatively poor. However, improvements in long read sequencing are making it easier to generate high quality sequences for complex genomes.

We now report a comparison of three long read sequencing methods applied to the de novo sequencing of a plant, *Macadamia jansenii*. This is a rare species that is a close relative of the macadamia nut recently domesticated in Hawaii and Australia. In the wild, it grows as a multi-stemmed, evergreen tree reaching 6-9 m height with leaves having entire margins and generally in whorls of three. The nuts are small (11-16 mm diameter) and have a smooth, hard, brown shell which encloses a cream, globulose kernel that is bitter and inedible [1]. The species was discovered as a single population of about 60 plants in the wild in Eastern Australia [2]. This is a flowering plant (angiosperm) in the Proteaceae family that is basal to the large eudicot branch of the flowering plant phylogeny [3]. The genomes of this group are poorly characterised, with most well sequenced plant genomes being either core eudicots or monocots that are plants of economic importance [4]. Knowledge of the genome of this species will support efforts to conserve the endangered species in the wild and capture novel traits such a small plant stature for use in plant breeding. Sequencing of wild crop relatives is urgent as many populations are critical to diversification of crop genetics to ensure food security in response to climate change [5] but are also threatened with extinction due to changes in land use or climate [6].

The macadamia genus contains four species: *Macadamia integrifolia*, *Macadamia tetraphylla*, *Macadamia ternifolia* and *Macadamia jansenii*. Macadamia cultivars are diploid (2n = 28) with k-mer based genome size estimates ranging from 758 Mb for *M. tetraphylla* [7] to 896 Mb for *M. integrifolia* [8]. The first draft genome assembly of the widely grown *Macadamia integrifolia* cultivar HAES 741 was constructed from short-read Illumina sequence data and was highly fragmented (518 Mb, 193,493 scaffolds, N50 = 4,745 bp) [9]. An improved HAES 741 assembly was generated using a combination of long-read PacBio and paired-end Illumina sequence data (745 Mb, 4,094 scaffolds, N50 = 413 kb) [8]. The genome assembly of *Macadamia tetraphylla* was also recently produced using a combination of long-read ONT and short-read Illumina sequence data (751 Mb, 4,335 contigs, N50 = 1.18 Mb) [7].

Long read sequencing provides data that facilitates easier assembly of the genome than is possible with short reads [10, 11, 12]. The length and sequence quality delivered by the available sequencing platforms has continued to improve. The reads produced can be used to assemble contigs or as a scaffold for the assembly of contigs generated with these techniques or from short reads [13]. Currently, Pacific Biosciences and Oxford Nanopore Technologies are the most commonly used technologies to generate long reads. Single-molecule real-time sequencing, developed by Pacific Biosciences can generate reads in the tens of kilobases using the continuous long read sequencing mode thus enabling high-quality de novo genome assembly. Oxford Nanopore Technologies enables direct and real-time sequencing of long DNA or RNA fragments by analysing the electrical current disruption caused by the molecules as they move through a protein nanopore. More recently, BGI has introduced the single tube Long Fragment Read (stLFR) [14] technology as an alternative to the generation of real long reads. stLFR is based on DNA co-barcoding [15, 16], that is adding the same barcode sequence to sub-fragments from the original long DNA molecule. In the stLFR process, the surface of microbeads are used to create millions of miniaturized barcoding reactions in a single tube. Importantly, stLFR enables near single molecule co-barcoding by using a large excess of microbeads and a combinatorial process to make around 3.6 billion unique barcode sequences. For this reason it is expected to enable high-quality and near complete de novo assemblies. Here we compare Sequel I (Pacific Biosciences), PromethION (Oxford Nanopore Technologies) and stLFR (BGI) data for the same DNA sample and evaluate the quality of the assemblies that can be generated directly from these data sets.

Methods

Plant material

Young leaves (40 g) of *Macadamia jansenii* were sourced from a tree with accession number 1005 and located at the Maroochy Research Facility, Department of Agriculture and Fisheries, Nambour 4560, Queensland, Australia. The specimen of *Macadamia jansenii* used in these experiments was a clonally propagated ex-situ tree planted in the arboretum at Maroochy Research Facility. None of the leaves used in these experiments were collected from wild in-situ trees. Young leaves were harvested, placed in on ice in bags and within 3 h snap frozen under liquid nitrogen and stored at -20°C until further processed for tissue pulverisation using either a mortar and pestle or the Mixer Mill as outlined below.

Genomic DNA extraction

Leaf tissue (10 g) was first coarsely ground under liquid Nitrogen using a mortar and pestle. The mortar and pestle with the coarsely ground tissue with residual liquid nitrogen was then placed on dry ice. This step ensured the temperature of the coarsely ground tissue was maintained close to -80°C while allowing the liquid nitrogen to evaporate off completely, an essential requirement for the pulverisation step. The coarsely ground leaf tissue was pulverised into fine powder in 50 ml steel jars using the Mixer Mill MM400 (Retsch, Germany). The pulverised leaf tissue was stored at -20°C until further required for DNA extraction. Genomic DNA (gDNA) was isolated from pulverised leaf tissue according to [17], with some modifications. Using a liquid-nitrogen cooled spatula, frozen pulverised leaf tissue (3 g) was added to 50 ml tubes (Corning or Falcon) containing warm (40°C) nuclear lysis buffer (8 ml) and 5% sarkosyl solution (5 ml). Tubes were incubated at 40°C for 45 min with periodic (every 5 min) gentle mixing by inverting the tubes. RNA was digested by adding RNase solution (10 mg/ml), the contents gently mixed by inverting the tubes followed by incubation at room temperature for 10 min. Two chloroform extractions were undertaken as follows. Chloroform (10 ml) was added to the tubes and gently mixed by inverting the tubes 50 times. The tubes were centrifuged at

Compiled on: September 28, 2020.
Draft manuscript prepared by the author.

3,500×g for 5 min in a swing out bucket rotor. The supernatant was transferred into fresh 50 ml tubes and the chloroform extraction repeated twice. The supernatant was transferred to fresh 50 ml tubes and the DNA precipitated using isopropanol. For every 1 ml of the supernatant, 0.6 ml of Isopropanol was added, the content gently mixed by inverting the tubes 20 to 25 times. The tubes were incubated at room temperature for 15 min and then centrifuged at 3,500×g for 5 min in a swing out bucket rotor. The supernatant was discarded and the DNA pellet was washed off any co-precipitated salts by adding 10 ml of 70% ethanol and incubating the tubes at room temperature for 30 min. The tubes were centrifuged at 3,500×g for 5 min in a swing out bucket rotor, the supernatant discarded and the DNA pellet semi dried to remove any residual 70% ethanol by incubating the tubes for 10 min upside down over filter paper. The DNA was dissolved by adding 100 µl of TE buffer and then adding incremental 50 µl of TE buffer where required. The DNA solution was transferred to 2 ml nuclease-free tubes and then centrifuged at 14,000×g for 45 min in a table top centrifuge. The supernatant was carefully transferred to fresh 2 ml tubes and the quality checked on a spectrophotometer and resolving the DNA on a 0.7% agarose gel. The DNA was then stored at -20°C until used for sequencing.

PacBio gDNA library preparation and sequencing

DNA sequencing libraries were prepared using the Template Prep Kit 1.0-SPv3 (PacBio, 100-991-900) according to the protocol for >30 kb SMRTbell Libraries (PacBio, Part # PN 101-024-600 Version 05). Genomic DNA (15 µg) was not fragmented, and was instead just purified with AMPure PB beads. The purified gDNA (10 µg) was treated with Exonuclease VII, followed by a DNA damage repair reaction, an end-repair reaction, and purification with AMPure PB beads. Adapters were ligated to the purified, blunt-ended DNA fragments in an overnight incubation. The adapter ligated sample was digested with Exonuclease III and Exonuclease VII to remove failed ligation products, followed by purification with AMPure PB beads. The purified sample was size selected using the Blue Pippin with a dye-free, 0.75% agarose cassette and U1 marker (Sage Science, BUF7510) and the 0.75% DF Marker U1 high-pass 30-40 kb vs3 run protocol, with a BPstart cut-off of 35000 bases. After size selection, the samples were purified with AMPure PB beads, followed by another DNA damage repair reaction, and a final purification with AMPure PB beads. The final purified, size-selected library was quantified on the Qubit fluorometer using the Qubit dsDNA HS assay kit (Invitrogen, Q32854) to assess the concentration, and a 0.4% Megabase agarose gel (Bio-Rad, 1613108) to assess the fragment size. Sequencing was performed using the PacBio Sequel I (software/chemistry v6.0.0). The library was prepared for sequencing according to the SMRT Link sample setup calculator, following the standard protocol for Diffusion loading with AMPure PB bead purification, using Sequencing Primer v3, Sequel Binding Kit v3.0 and the Sequel DNA Internal Control v3. The polymerase-bound library was sequenced on 8 SMRT Cells with a 10 h movie time using the Sequel Sequencing Kit 3.0 (PacBio, 101-597-900) and a Sequel SMRT Cell 1M v3 (PacBio, 101-531-000). Library preparation and sequencing was performed at the Institute for Molecular Bioscience Sequencing Facility (University of Queensland).

ONT library preparation and sequencing

The quality of the DNA sample was assessed in NanoDrop, Qubit, and the Agilent 4200 TapeStation system. The DNA sample was sequenced on the Oxford Nanopore Technologies (ONT)-MinION and PromethION. The MinION library was prepared from 1,500 ng input DNA using the ligation sequencing kit (SQK-LSK109, ONT) according to the manufacturer's protocol except the End-repair and end-prep reaction and ligation period were increased to 30 min. Third party reagents NEBNext end repair/dA-tailing Module (E7546), NEBNext FFPE DNA Repair Mix(M6630), and NEB Quick Ligation Module (E6056) were used during library preparation. The adapters-ligated DNA sample was quantified using Qubit® dsDNA HS Assay Kit (Thermofisher). The MinION flowcell R9.4.1 (FLO-MIN106, ONT) was primed according to the manufacturer's guidelines before loading a library mix (75 µl) containing 438 ng of adapters-ligated DNA, 25.5 µl LB (SQK-LSK109, ONT), and 37.5 µl SQB (SQK-LSK109, ONT). The MinION sequencing was performed using MinKNOW (v1.15.4), and a standard 48 h run script. Before preparing the PromethION library, short DNA fragments (<10 kb) were first depleted from DNA sample (9 µg) as described in the manufacturer's instructions for the Short Read Eliminator (SRE) kit (SKU SS-100-101-01, Circulomics Inc). The PromethION library was prepared from 1200 ng SRE-treated DNA using ligation sequencing kit (SQK-LSK109, ONT). All steps in the library preparation were the same as the MinION library preparation except the adapters-ligated DNA was eluted in 25 µl of Elution Buffer. The PromethION flowcell (FLO-PRO002) was primed according to the manufacturer's guidelines before loading a library mix (150 µl) containing 390 ng of adapters-ligated DNA (24 µl), 75 µl of SQB and 51 µl of LB (SQK-LSK109, ONT). Sequencing was performed using MinKNOW (v3.1.23), and a standard 64 h run script. The sequencing run was stopped at 21 h and nuclease flush was performed to recover clogged pores. The Nuclease flushing mix was prepared by mixing 380 µl of Nuclease flush buffer (300 mM KCl, 2 mM CaCl2, 10 mM MgCl2, 15 mM HEPES pH 8) and 20 µl of DNase I (M0303S, NEB). The Nuclease Flushing mix was loaded into the flow cell and incubated for 30 min. The flow cell was then primed as mentioned above and loaded with the fresh library mix (150 µl) containing 390 ng of adapters-ligated DNA and rerun the standard 64 h run script using MinKNOW. Refuelling of the sequencing run was performed at each 24 h by adding 150 µl of diluted SQB (1:1, SQB:nuclease free water) to keep the stable translocation speed of sequencing. ONT fast5 reads were basecalled using Guppy v3.0.3 with the config file dna_r9.4.1_450bps_hac_prom.cfg (PromethION) or dna_r9.4.1_450bps_hac.cfg (MinION) and parameters --qscore_filtering -q 0 --recursive --device "cuda:0 cuda:1 cuda:2 cuda:3".

BGI library preparation and sequencing

stLFR sequencing libraries were prepared using the MGIEasy stLFR Library Prep Kit (MGI, Shenzhen, China) following the manufacturer's protocol. Briefly, genomic DNA samples were serially diluted and then quantified using the Qubit™ dsDNA BR Assay Kit (Invitrogen, Carlsbad, CA) and the Qubit™ dsDNA HS Assay Kit (Invitrogen, Carlsbad, CA) for a more accurate quantification result. Around 1.5 ng of original genomic DNA molecules were used for library preparation. In the first step, transposons composed of a capture sequence and a transposase recognition sequence were inserted at a regular interval along the genomic DNA molecules. Next, these transposon inserted DNA molecules were hybridized with barcode labelled 3 µm diameter magnetic beads containing oligonucleotide sequences with a PCR primer annealing site, an stLFR barcode, and a sequence complementary to the capture sequence on the transposon. After hybridization, the barcode was transferred to the transposon inserted DNA sub-fragments through a ligation step. The excess oligonucleotides and transposons were then digested with exonuclease and the transposase enzyme was de-

natured with sodium dodecyl sulfate. Next, the second adapter was introduced by a previously described 3'-branch ligation using T4 ligase [18]. Finally, PCR amplification was performed using primers annealing to the 5' bead and 3'-branch adapter sequences. The PCR reaction was purified using Agencourt® AMPure XP beads (Beckman Coulter, Brea, CA) and quantified using the Qubit™ dsDNA HS Assay Kit (Invitrogen, Carlsbad, CA). The PCR product fragment sizes were assessed using an Agilent High Sensitivity DNA Kit (Agilent, 5067-4626) on a Agilent 2100 Bioanalyzer. The average fragment size of the prepared stLFR library was 1003 bp. 20 ng of PCR product from the stLFR library was used to prepare DNA Nano Balls (DNBs) using the MGISEQ-2000RS High Throughput stLFR Sequencing Set (MGI, Shenzhen, China) following the manufacturer's protocol. The prepared DNB library was loaded onto two lanes of a MGISEQ-2000RS flow cell (MGI, Shenzhen, China) and then sequenced on a MGISEQ-2000RS (MGI, Shenzhen, China) using the MGISEQ-2000RS stLFR sequencing Set (MGI, Shenzhen, China). Library preparation and sequencing were performed at the BGI Australia Sequencing Facility (CBCRC Level 6, Herston, QLD) and BGI-Shenzhen (Shenzhen, China).

Illumina sequencing

Illumina library was prepared using the Nextera Flex DNA kit. The library was sequenced on an SP flow cell (14%) of the Illumina Nova Seq 6000 sequencing platform (The Ramaciotti Centre, University of New South Wales, Australia) using the paired-end protocol to produce 112 million 150 bp reads in pairs, an estimated 43× genome coverage. The median insert size was 713 bp.

Sequence read preparation

ONT read length and quality was calculated with NanoPlot v1.22 [19]. Long reads from PacBio and ONT were prepared using two or three alternative strategies respectively:

- All: no filtering of reads
- Filtered: ONT long reads were adapter-trimmed using Porechop v0.2.4 (Porechop, RRID:SCR_016967) [20]. ONT and PacBio reads were filtered using Filtlong v0.2.0 [21] by removing 10% of the worst reads and reads shorter than 1 kb.
- Pass (ONT only): only the passed reads were used (average base call quality score above 7).

The PacBio subreads were randomly subsampled down to a 32× genome coverage using Rasusa v0.1.0 [22]. Raw Illumina and BGI short reads were adapter-trimmed using Trimmomatic v0.36 (Trimmomatic, RRID:SCR_011848) [23] (LEADING:3 TRAILING:3 SLIDINGWINDOW:4:15 ILLUMINACLIP:2:30:10 MINLEN:36). PolyG tail trimming was performed on the Illumina reads using fastp v0.20.0 (fastp, RRID:SCR_016962) [24].

Genome size estimation

K-mer counting using the trimmed Illumina and BGI reads was performed using Jellyfish v2.210 (Jellyfish, RRID:SCR_005491) [25] generating k-mer frequency distributions of 21-, 23- and 25-mers. The histograms of the k-mer occurrences were processed by GenomeScope (GenomeScope, RRID:SCR_017014) [26], which estimated a genome haploid size of 653 and 616 Mb with around 71% and 74% of unique content and a heterozygosity level of 0.65% and 0.77% from Illumina and BGI reads respectively.

Assembly of genomes

De novo assembly of ONT and PacBio reads were performed using Redbean v2.5 (WTDBG, RRID:SCR_017225) [27], Flye v2.5 (Flye, RRID:SCR_017016) [28], Canu v1.8 (ONT) or v1.9 (PacBio) (Canu, RRID:SCR_015880) [29], Raven v1.1.6 [30] with default parameters. For Redbean, Flye and Canu, the estimated genome size was set to 780 Mb [31]. For ONT data, four rounds of error correction were performed using Racon v1.4.9 (Racon, RRID:SCR_017642) [32] with recommended parameters (-m 8 -x -6 -g -8 -w 500) based on minimap2 v2.17-r943-dirty [33] overlaps, followed by one round of Medaka v0.8.1 [34] using the r941_prom_high model to create the consensus sequence. The resulting sequence was polished with Pilon v1.23 (Pilon, RRID:SCR_014731) [35] using the Illumina reads mapped with BWA-MEM v0.7.13 (BWA, RRID:SCR_010910) [36] and with the settings to fix bases (--fix bases). Polishing of the Medaka consensus sequence with Illumina reads was also performed by NextPolish v1.1.0 [37] with default settings (BWA for the mapping step). Hybrid assembly was generated with MaSuRCA v3.3.3 (MaSuRCA, RRID:SCR_010691) [38] using the Illumina and the ONT or PacBio reads and using Flye v2.5 to perform the final assembly of corrected mega-reads (parameter FLYE_ASSEMBLY=1). Diploid de novo genome assembly of PacBio reads was performed with FALCON v1.3.0 (FALCON, RRID:SCR_016089) [39] using a genome size of 780 Mb, a length cutoff of 40,740 bp and a seed read coverage cutoff of 30. A total of 19 Gb of preassembled reads was generated (24× coverage). After assembly and haplotype separation by FALCON-Unzip v1.2.0 [39], polishing was performed as part of the FALCON-Unzip workflow. PacBio reads were mapped to the primary FALCON-Unzip assembly using minimap2 v2.17-r954-dirty [33]. A read coverage histogram was generated from this alignment using Purge Haplotigs v.1.1.0 [40] to obtain the read depth cutoff values (-l 17 -m 52 -h 190) required to identify redundant contigs. Illumina reads were assembled using SPAdes v3.13.1 (SPAdes, RRID:SCR_000131) [41].

Two lanes of stLFR reads for the same sample were demultiplexed using a sub-function of SuperPlus v1.0 [42] and combined for the downstream analysis. Adapter sequences were removed from read data using Cutadapt v2.4 (cutadapt, RRID:SCR_011841) [43] with the recommended parameters (--no-indels -O 10 --discard-trimmed -j 42). Read sequences were then converted to 10X Genomics format by BGI's in-house software, which contains three steps: 1) Change the format of reads' head from MGI to Illumina. 2) Change the quality number of "N" base from 33 (ASIC II code = !) to 35 (ASIC II code = #) to meet the 10X Genomics' quality system. 3) Merge two or more barcodes into one barcode randomly due to the limitation of barcode types for 10x Genomics. To meet the memory requirement of the assembler, the barcodes with less than 10 reads were removed from the dataset. De novo assembly was performed by Supernova v2.1.1 (Supernova assembler, RRID:SCR_016756) [44] using the suggested parameters (--maxreads=2100000000 --accept-extreme-coverage --nopreflight). TGS-GapCloser v1.0.0 (TGS-GapCloser, RRID:SCR_017633) [45, 46] was used to fill the gaps between contigs within same scaffolds, and this process was performed under the use of error-corrected ONT or PacBio data by Canu. The number of gaps within scaffolds was computed using the formula: number of contigs - number of scaffolds.

The technical specifications of the computing clusters used in this study are provided in Table S10. An estimation of computational costs based on Amazon EC2 on-demand pricing is provided in Table S11.

Table 1. Sequencing data

Dataset	ONT	PacBio	BGI	Illumina
Number of raw reads	3,129,385	3,170,206	738,145,698	112,508,072
Number of trimmed reads	-	-	611,835,983	109,046,265
Reads used in assembly	3,129,385	3,170,206	372,797,279	109,046,265
Number of bases	24,915,207,810	65,228,232,554	74,559,455,800	31,961,393,885
Read length N50	27,842	35,866	2×100	2×150
Mean read length	7,962	20,575	2×100	2×150
Genome coverage	32	84	96	41
Cost (USD)*	3,270	12,560	1,120	721
Sequencing date	March/April 2019	June 2019	May/June 2019	April 2019
DNA amount (ng)	1,200-1,500	15,000	10	500

*Australian dollars costs were converted to US dollars at an exchange rate of 0.685 USD/AUD. The ONT cost includes library preparation (400 USD) and sequencing on one PromethION flow cell (2,050 USD) and one MinION flow cell (820 USD). The PacBio cost includes library preparation (1,187 USD) and sequencing on 8 SMRT cells (11,373 USD). The stLFR cost is estimated based on the number of raw reads subsequently used in assembly (~90 Gb) and includes library preparation (400 USD) and sequencing (8 USD per Gb). Genome coverage estimates were computed based on the number of reads used in assembly and an estimated genome size of 780 Mb.

Assembly evaluation

Assembly statistics were computed using QUAST v5.0.2 (QUAST, RRID:SCR_001228) [47] with a minimum contig length of 10 kb and the parameters --fragmented --large. The publicly available reference genome of *Macadamia integrifolia* v2 (Genbank accession: GCA_900631585.1) [8] was used as the reference genome for QUAST. To estimate the base accuracy, QUAST was used to compute the number of mismatches and indels as compared to the Illumina short-read assembly generated by SPAdes. The Illumina short read assembly was generated using more accurate short reads as compared to long reads therefore it contained fewer base errors. Consequently the number of mismatches and indels identified in the long-read assemblies as compared to the short-read assembly will reflect their base error rates. We noted that this would only enable comparison to X% of the genome since the Illumina only assembly is relatively incomplete. Furthermore the Illumina assembly would be expected to have errors and those errors would result in calling errors in other assemblies even when they are actually correct. To evaluate the completeness of the genome, the assemblies were subjected to the Benchmarking Universal Single-Copy Orthologs v3.0.2 (BUSCO, RRID:SCR_015008) [48] with the eudicotyledons_odb10 database (2121 genes). The K-mer Analysis Toolkit v2.4.2 (KAT, RRID:SCR_016741) [49] comp and kat_distanalysis commands were used to estimate k-mer assembly completeness by reference to the Illumina or stLFR short reads.

Results

Illumina genome assembly

Illumina sequencing generated 112.5 million 150 bp paired-end reads, which correspond to approximately 41× coverage of the genome. After adapter and polyG tail trimming, short reads were assembled using the SPAdes software. The resulting assembly consisted of 1,631,183 contigs totaling 864 Mb in length and contained 15,583 contigs larger than 10 kb with a total length of 338 Mb (Table S1). The assembly was highly fragmented with a contig N50 of 23.9 kb. Genome completeness assessment using BUSCO revealed that the assembly contained 65% of complete BUSCOs (including 58% of single-copy genes), 18% of fragmented BUSCOs and 17% of missing BUSCOs.

ONT genome assembly

For the ONT sequencing, we combined the results of one PromethION and one MinION flow cell, generating a total of 24.9 Gb of data with a read length N50 of 27.8 kb (Table 1). The PromethION flow cell and the MinION flow cell generated 23.2 Gb and 1.7 Gb of data respectively, with a read length N50 of 28.5 kb and 16.6 kb and a median read quality of 6.3 and 8.9. ONT reads were assembled using four different long-read assemblers (Redbean, Flye, Canu, Raven) and three different read subsets representing different genome coverage (21×, 28× and 32×). The statistics for each assembly are shown in Table S2 and Fig. S1. Canu and Flye generated the largest and most contiguous assemblies while Redbean produced the smallest and less contiguous assembly (~750 Mb, contig N50 ~700 kb) followed by Raven (~770 Mb, contig N50 ~1 Mb). Flye consistently produced assemblies of around 812 Mb with a contig N50 of approximately 1.5 Mb whereas Canu and Redbean assembly contiguity increased as the read coverage increased. In particular, the Canu contig N50 significantly increased from 706 kb (21×) to 1.43 Mb (32×). For 28x and 32x genome coverage, Raven assemblies were similar in size (Raven is the only tool that does not require an estimated genome size as a mandatory input parameter). Raven was the only tool ran on a GPU-accelerated server and it was the fastest assembler, followed by Redbean and Flye. Canu was approximately five times and ten times slower than Flye and Redbean respectively.

We subsequently polished the Redbean, Flye, Canu and Raven draft assemblies using the ONT long reads followed by the Illumina short reads. Long-read polishing was performed using the Racon and Medaka tools. Two softwares to fix base errors using short reads were compared: the widely used tool Pilon and the recently developed algorithm NextPolish. Those polishing steps greatly improved the genome completeness as indicated by the percentage of complete BUSCOs which increased from 53% (Redbean), 70% (Canu) or 79% (Flye, Raven) to 85% (Redbean) or 89% (Flye, Raven, Canu) after long-read polishing and 92% (Redbean) or 95% (Flye, Raven, Canu) after long-read and short-read polishing (Table S3). As an estimation of the base accuracy, we computed the number of mismatches and indels as compared to the Illumina short-read assembly generated by SPAdes (Fig. S2 and Table S7). The Canu assembly was less accurate than the other assemblies (NextPolish: 582 vs 485-503 mismatches per 100 kb, 68 vs 42-49 indels per 100 kb; Pilon: 670 vs 529-593 mismatches per 100 kb, 108 vs 76-85 indels per 100 kb) and contained a higher percentage of duplicated genes (16-17% vs 12-14%).

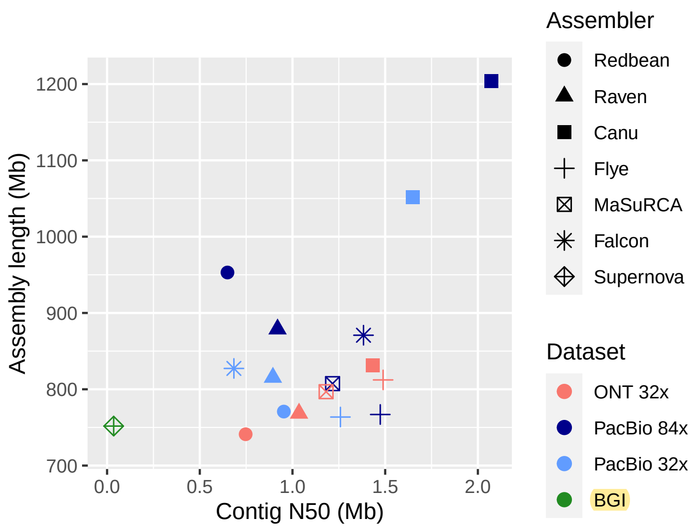

Figure 1. ONT, PacBio and BGI genome assembly statistics. The total assembly length is plotted against the contig N50 for each assembler and sequencing dataset.

The base accuracy metrics suggest that NextPolish performed slightly better than Pilon. In particular, the number of indels was greatly reduced after polishing with NextPolish as compared to Pilon (Flye: 48 vs 83 indels per 100 kbp, Canu: 68 vs 108, Raven: 49 vs 85, Redbean: 42 vs 76, Table S7). Pilon and NextPolish resulted in similar genome completeness when applied to the Canu and Raven assemblies. The genome completeness was slightly better after two iterations of NextPolish than after two iterations of Pilon for the Flye (95.4% vs 95.2%) and Redbean assemblies (91.9% vs 91.6%). A second iteration of Pilon resulted in a slight decrease in the number of missing genes and a higher accuracy for all four assemblers whereas a second iteration of NextPolish did not improve the genome completeness and accuracy (mismatches) for the Canu and Raven assemblies. Therefore, depending on the assembler and the polisher used, the number of recommended polishing iterations might be different.

Assembly completeness was also estimated by comparing the k-mer spectrum of the polished assemblies to the k-mer spectrum of the Illumina short-reads (Table S8 and Fig. S4). The k-mer analysis suggested that Flye produced the most complete polished assembly (99%) followed by Canu (97.9%) and Raven (97.4%) and finally Redbean (92.3%). The trends were similar when the k-mer analysis was performed using the stLFR short-reads.

As an alternative method to long-read-only assembly followed by polishing with short reads, an hybrid assembly was generated using MaSuRCA. The ONT + Illumina assembly showed a similar size (797 Mb), contiguity (contig N50 = 1.18 Mb), completeness (94.8% complete BUSCOs including 15.5% duplicated BUSCOs) and a slighlty lower accuracy (530 mismatches per 100 kb, 53 indels per 100 kb) as the Flye and Raven assemblies with subsequent polishing with Illumina reads (Fig. 1, 2, 3 and S2, Table S2 and S3). Short-read polishing or long-read followed by short-read polishing did not significantly improve the genome completeness of the MaSuRCA assembly (Table S3), which is expected as the super-reads constructed by this tool are based on the Illumina reads.

PacBio genome assembly

With eight single-molecular real-time cells in the PacBio Sequel platform, we generated 3,170,206 subreads with a read length N50 of 35.9 kb and representing a total of 65.2 Gb (Table 1). The data correspond to ~84× coverage of the estimated 780 Mb genome size. The assembly of the PacBio data was conducted using the same tools used for the ONT data: the four long reads assemblers: Redbean, Flye, Canu and Raven and the hybrid assembler MaSuRCA (Table S4). The PacBio assemblies showed a similar contiguity as the ONT assemblies (except Canu) and were larger in size (except Flye) (Fig. 1). Before polishing, their genome completeness was higher than the ONT assemblies indicating a higher accuracy of PacBio reads (Fig. S3). The Redbean assembly was the most fragmented (contig N50 = 649 kb) and the least complete (89% complete BUSCOs). The Flye assembly was highly contiguous (contig N50 = 1.47 Mb) and the smallest in size (767 Mb). The Raven assembly (879 Mb) consisted of the least number of contigs (n = 1,730) with a contig N50 of 919 kb. The Canu assembly was the largest (1.2 Gb) but it contained a high fraction of duplication as reported by QUAST (1.64) and confirmed by the percentage of duplicated BUSCOs (53%) and the k-mer spectra (Fig. S4). Therefore, the Canu assembly likely contains uncollapsed haplotypes corresponding to artefactually duplicated regions, as reported recently [50]. Aligning the PacBio assemblies to the *Macadamia integrifolia* assembly identified a higher number of misassemblies in the Canu asssembly (n = 38,800) as compared to the other assemblies (n = 21,000-27,000). The PacBio + Illumina hybrid assembly (807 Mb, contig N50 = 1.22 Mb) contained 94.9% of complete BUSCOs including 16% of duplicated genes (Fig. 3).

In order to generate a phased diploid assembly, PacBio assembly was next performed using the FALCON assembler, followed by haplotype resolution and polishing using FALCON-Unzip. The resulting primary assembly consisted of 1,333 contigs totaling 871 Mb in length, with half of the assembly in contigs of 1.38 Mb or longer (Fig. 1). FALCON-Unzip also generated 2,488 alternate haplotigs spanning 495 Mb (i.e. 57% of the genome was haplotype-resolved), with a contig N50 of 333 kb. BUSCO analysis on primary contigs showed around 26% of duplicated genes suggesting the presence of homologous primary contigs (Fig. 3). The Purge Haplotigs pipeline identified 569 primary contigs representing 112 Mb

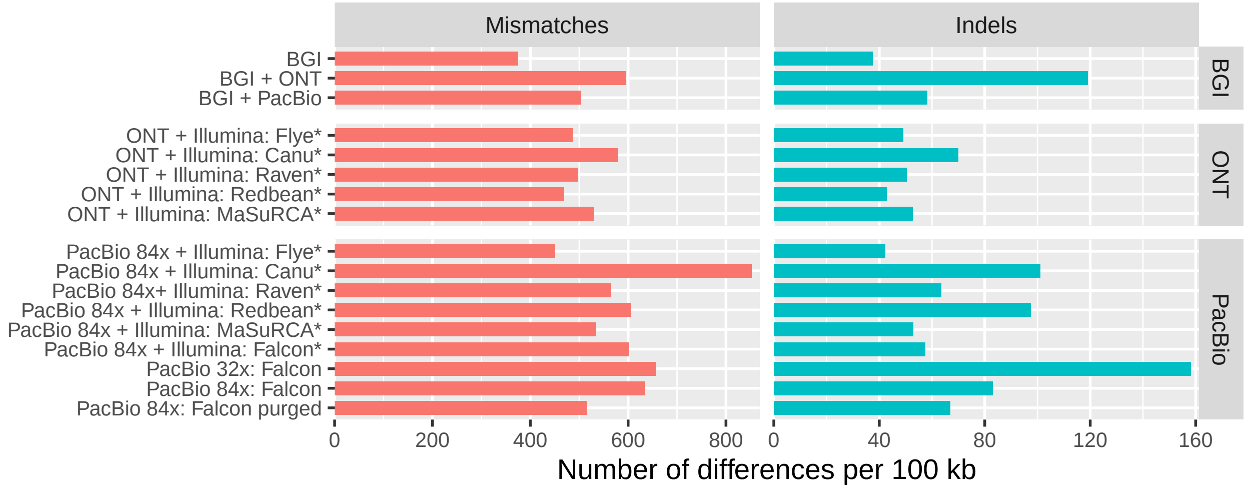

Figure 2. Number of mismatches and indels identified in the long-read assemblies as compared to the Illumina short-read assembly generated by SPAdes. The BGI + ONT and BGI + PacBio assemblies were polished with the BGI stLFR reads using one iteration of NextPolish. The ONT + Illumina assemblies (except MaSuRCA) were polished with the ONT long-reads using Racon and Medaka followed by the Illumina short-reads using one iteration of NextPolish. The PacBio + Illumina assemblies (except MaSuRCA) were polished with the Illumina short-reads using one iteration of NextPolish. (*Assembly polished using Illumina reads)

as likely alternate haplotypes (Table S5). These contigs were transferred to the haplotigs set. The curated primary haploid assembly consisted of 762 contigs totaling 758 Mb with a contig N50 of 1.59 Mb and contained less duplicated genes (16%) with minimal impact on genome completeness (95% complete BUSCOs).

We subsequently polished the PacBio assemblies using the Illumina short reads. As expected, a reduced number of mismatches and indels was identified in the assemblies as compared to the Illumina assembly (Fig S2 and Table S7). Polishing decreases the number of missing BUSCOs but increased the number of duplicated BUSCOs for the Redbean, Flye and Raven assemblies (Table S6). Long-read followed by short-read polishing resulted in an increased percentage of single-copy BUSCOs and a reduced percentage of duplicated BUSCOs for the Canu assembly and, to a lesser extent, the Falcon assembly. Interestingly, the long-read polishing step did not improve the completeness of the Redbean, Flye and Raven assemblies and similar or slightly better results were obtained after short-read polishing alone. Therefore, the recommended polishing strategy for PacBio assemblies might depend on the assembler used.

Using a quality filtered subset of the subreads (equivalent to ~67× genome coverage) led to a similar (Flye and Raven) or slightly higher (Redbean) assembly contiguity without impacting on the genome completeness (only Redbean, Raven and Flye were tested due to the high computational requirements of Canu and Falcon) (Fig. S1 and Table S4). Finally, in order to compare PacBio and ONT technologies, we randomly subsampled the PacBio subreads down to a coverage equivalent to the ONT data (32×). The resulting Flye assembly showed a similar size of 764 Mb, a lower contiguity (contig N50 = 1.26 Mb) and a similar genome completeness (94.7% complete BUSCOs) as the 84× coverage assembly (Fig. 1, S3 and Table S4). The other four assemblers resulted in a reduced genome size and a slightly lower genome completeness. The decrease in coverage did not impact on the Raven assembly contiguity (contig N50 = 894 kb). The Falcon assembly was the most affected by the coverage drop with a decrease in the contig N50 from 1.38 Mb to 684 kb. Conversely, the Redbean assembly contiguity increased from 649 kb to 953 kb. The percentage of duplicated BUSCOs decreased for all the assemblies but remained high for the Canu (33%) and Falcon (20%) assemblies.

stLFR genome assembly

stLFR generated 738 million 100 bp paired-end reads. To meet the requirements of the assembler, the barcodes with less than 10 reads were removed which resulted in 373 million reads representing 74.6 Gb of data and corresponding to approximately 96× coverage of the genome (Table 1). stLFR reads were assembled using Supernova2 into an assembly of 40,789 scaffolds totaling 880 Mb in length (Table S9). 5,065 scaffolds were larger than 10 kb with a total length of 752 Mb and a N50 of 3.54 Mb for scaffold and 35.6 kb for contig (Table 2). When compared to the Illumina short-read assembly, the stLFR assembly contained the lowest number of mismatches and indels (Fig. 2). Conserved BUSCO gene analysis revealed that the stLFR assembly contained 88.3% of complete genes from the eudicotyledons dataset (Fig. 3).

Inclusion of ONT or PacBio data to fill the gaps within scaffolds led to a 29-fold or 45-fold increase in the contig N50 length from 35.6 kb to 1.05 Mb or 1.60 Mb and a 22-fold or 55-fold decrease in the number of gaps within scaffolds larger than 10 kb from 14,889 to 690 or 271 (Table 2). The scaffold N50 slightly dropped by 0.02 Mb or 0.04 Mb due to the adjustment of the estimated gaps. For both gap-filled assemblies, the total assembly length increased correspondingly to around 895 Mb and 770 Mb for scaffolds larger than 10 kb. The largest contig size increased from 518 kb to 9.7 Mb (ONT) and 23.8 Mb (PacBio). In addition, the genome completeness was improved in the gap-filled assemblies, with BUSCO detecting 4.8% (ONT) and 5.8% (PacBio) more complete genes. The number of complete duplicated BUSCOs was slightly lower in the ONT filled assembly (12%) than in the PacBio filled assembly (14%). Finally, the estimated k-mer assembly completeness increased in the gap-filled assemblies from 95.8% to 96.7% (ONT) and 97.4% (PacBio) (Table S8). Further polishing of gap-filled assemblies using the stLFR reads resulted in a slight increase in the genome completeness to 93.2% (ONT) and 93.7% (PacBio)

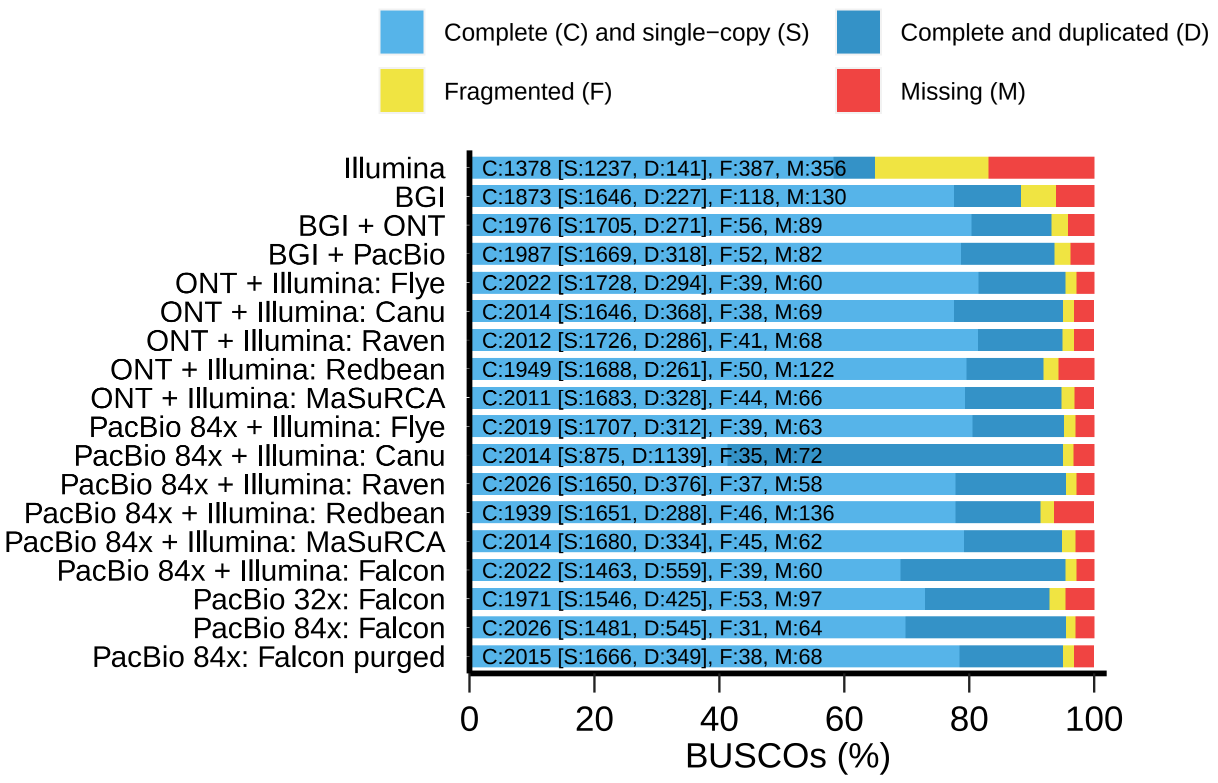

Figure 3. BUSCO analysis of assemblies using the eudicotyledons dataset (2121 genes). The x-axis depicts the percentage of complete and single-copy, complete and duplicated, fragmented and missing BUSCOs and the y-axis indicates the assembly assessed. The BGI + ONT and BGI + PacBio assemblies were polished with the BGI stLFR reads using one iteration of NextPolish. The ONT + Illumina assemblies (except MaSuRCA) were polished with the ONT long-reads using Racon and Medaka followed by the Illumina short-reads using one iteration of NextPolish. The PacBio + Illumina assemblies (except MaSuRCA) were polished with the Illumina short-reads using one iteration of NextPolish.

of complete BUSCO genes (Table S9 and Fig. S3) and a decrease in the number of indels (Table S8 and Fig. S2).

Discussion

We report a comparison of three long-read sequencing datasets generated from the same plant DNA sample. *M. jansenii* was selected for this study because of its significance in conservation and breeding. All four species of *Macadamia* are listed as threatened under Australian legislation but *M. jansenii* is particularly vulnerable given it has been recorded at only one location. *M. jansenii* has not been domesticated and its small and bitter nuts are obstacles that restrict simple introgression in breeding. However, the characteristic small tree size, being 50% smaller than commercial cultivars, is of interest for use in high-density orchard design and it is being trialled as a rootstock for this purpose [51]. It is the most northern *Macadamia* species and may be a source of genes for adaptation to warmer climates [52]. Hybrids of *M. integrifolia* and *M. jansenii* have been produced.

The three long-read sequencing technologies significantly improved the assembly completeness as compared to the assembly produced using the Illumina reads only (65% of complete BUSCOs). The cost of generating 1 Gb of sequencing data (including the library preparation) was 193 USD for PacBio Sequel I, 97 USD for ONT PromethION and 12 USD for BGI stLFR (raw reads subsequently used in assembly). Virtual long reads were generated using the stLFR protocol. This technology benefits from the accuracy and the low cost of a short-read sequencing platform while providing long range information. stLFR was the cheapest approach and it generated an assembly with the fewest single base and indel errors. Furthermore, the assembly generated by Supernova was phased. That said, the stLFR assembly was more fragmented than the other long-read technologies. We also demonstrated that stLFR could be used as a complementary technology to ONT. Indeed, the inclusion of Nanopore reads significantly increased the stlFR assembly contiguity with a N50 reaching 1 Mb and improved the genome completeness. Interestingly, the gap-filling step only used 1.7% of the ONT reads, suggesting that a real-time selective sequencing approach could be used to select specific molecules that would be informative for filling the gaps [53].

When all the reads were incorporated, the assemblies generated using the PacBio and ONT data were comparable in terms of assembly contiguity (contig N50 of around 1.5 Mb) and genome completeness (95% of complete BUSCOs). However, when we utilised the same amount of data for each platform (32× coverage), the contiguity of the PacBio assembly produced by Falcon was halved and became only half the size of the ones from the ONT Flye or Canu assemblies. The Flye and Raven assemblers proved to be more robust to the PacBio coverage drop as the assembly contig N50 only dropped from 1.47 Mb to 1.26 Mb (Flye) and from 919 kb to 894 kb (Raven). Additionally, we found that polishing the ONT assembly with the Illumina short reads was required to reach a similar genome completeness to

Table 2. Gap filling for stLFR assembly using error-corrected ONT or PacBio reads

	Supernova	After Gap Filling ONT	Improvement ONT	After Gap Filling PacBio	Improvement PacBio
Number of input long-reads	–	1,056,095	–	674,796	–
Useable reads for filling	–	1.74%	–	2.95%	–
Number of scaffolds	5,065	5,332	5.3% ↑	5,446	7.5% ↑
Scaffold N50	3,540,919	3,523,921	0.5% ↓	3,504,721	1.0% ↓
Scaffold length	751,745,340	766,968,089	2.0% ↑	768,468,395	2.2% ↑
Largest scaffold size	30,143,475	31,148,326	3.3% ↑	31,237,530	3.6% ↑
Number of contigs	19,954	6,022	70% ↓	5,717	71% ↓
Contig N50	35,605	1,046,570	2839% ↑	1,598,608	4390% ↑
Contig length	594,029,544	742,770,175	25% ↑	758,126,937	28% ↑
Largest contig size	517,998	9,683,794	1769% ↑	23,824,472	4499% ↑
Number of gaps within scaffolds	14,889	690	95% ↓	271	98% ↓
Number of Ns per 100 kb	16,934	3,042	82% ↓	1,290	92% ↓
Complete BUSCOs	1,873 (88.3%)	1,963 (92.5%)	4.8% ↑	1,983 (93.5%)	5.8% ↑
Complete single-copy BUSCOs	1,646 (77.6%)	1,710 (80.6%)	3% ↑	1,679 (79.2%)	1.6% ↑
Complete duplicated BUSCOs	227 (10.7%)	253 (11.9%)	1.2% ↑	304 (14.3%)	3.6% ↑

QUAST analysis was performed using a minimum contig size of 10 kb and the parameters --fragmented --large --split-scaffolds.

that of the PacBio assembly. For both ONT and PacBio data, the highest contiguity was obtained with a long-read polished assembly as compared to an hybrid assembly incorporating both the short and long reads.

Since the sequence data was generated, the PacBio SMRT platform has transitioned from the Sequel I to the Sequel II instrument, with a 8-fold increase in the data yield. The latest platform produces high-fidelity reads that are more accurate than the continuous long reads assembled in this study. Consequently the cost to generate a similar PacBio assembly on the Sequel II system will be dramatically reduced and the assembly quality is likely to improve while requiring less computational resources.

The DNA material requirements to prepare the sequencing library is another important parameter to consider when choosing a sequencing technology. For ONT sequencing, it is recommended to obtain at least 1-2 µg of high molecular weight DNA. The stLFR library construction requires at least 10 ng of high molecular weight DNA. PacBio SMRT sequencing has a high genomic DNA input requirements of 5-20 µg of high molecular weight DNA for standard library protocol depending on the genome size but the PacBio low DNA input protocol has reduced this requirement to as low as 100 ng per 1 Gb genome size [54]. Furthermore, PacBio recently released an amplification-based ultra-low DNA input protocol starting with 5 ng of high molecular weight DNA.

The computational requirements and associated cost should be considered and will largely depend on the genome size of the species of interest. There were important differences in the assembly run time and memory usage depending on the tool used. For instance, short-read polishing using NextPolish used less memory than Pilon, while providing similar results. GPU accelerated computing greatly reduced the computing time for some tools such as Racon, Medaka or Raven. There are also challenges associated with the rapid evolution of technologies and softwares. For example we observed a significant improvement in the ONT assembly contiguity depending on the basecaller or assembler version used. The newest releases of assemblers such as Canu v2.1, Flye v2.8 or Raven v1.1.10 will likely generate improved assemblies.

The three long-read technologies produced highly contiguous and complete genome assemblies. Next, long-range scaffolding approaches such as chromosome conformation capture (Hi-C, Chicago) or physical maps technologies (optical map, restriction map) are required to order and orient the assembled contigs into chromosome-length scaffolds [55].

Availability of supporting data and materials

BGI, PacBio, ONT and Illumina sequencing data generated in this study have been deposited in the Sequence Read Archive under BioProject PRJNA609013 and BioSample SAMN14217788. Accession numbers are as follows: BGI (SRR11191908), PacBio (SRR11191909), ONT PromethION (SRR11191910), ONT MinION (SRR11191911) and Illumina (SRR11191912).

Additional Files

Table S1: Illumina genome assembly statistics using SPAdes assembler
Table S2: ONT genome assembly statistics using Redbean, Flye, Canu, Raven and Masurca assemblers
Table S3: BUSCO genome completeness assessment of ONT long-read assemblies (Redbean, Flye, Canu, Raven) and hybrid assembly (MaSuRCA)
Table S4: PacBio genome assembly statistics using Redbean, Flye, Falcon, Canu, Raven and MaSuRCA assemblers
Table S5: PacBio genome assembly statistics and genome completeness assessment before and after Purge Haplotigs
Table S6: BUSCO genome completeness assessment of PacBio long-read assemblies (Redbean, Flye, Falcon, Canu, Raven) and hybrid assembly (MaSuRCA)
Table S7: QUAST assembly statistics using the Illumina short-read assembly as the reference genome
Table S8: K-mer completeness of ONT, PacBio and stLFR assemblies
Table S9: BGI stLFR genome assembly statistics using Supernova assembler and TGS-GapCloser gap-closing software
Table S10: Technical specifications of computing clusters
Table S11: Estimation of computational costs based on Amazon EC2 on-demand pricing as at 19/9/2020

Figure S1: Genome assembly statistics. The total assembly length is plotted against the contig N50 for each assembler and sequencing coverage. (A) ONT assemblies, (B) PacBio assemblies.
Figure S2: Number of mismatches and indels identified in the long-read assemblies as compared to the Illumina short-read assembly generated by SPAdes. (A) ONT assemblies before and after Illumina short-read polishing using one iteration of NextPolish (Flye, Canu, Raven, Redbean) and MaSuRCA hybrid assembly, (B) PacBio assemblies before and after Illumina short-read polishing using one iteration of NextPolish

(Falcon, Flye, Canu, Raven, Redbean) and MaSuRCA hybrid assembly, (C) BGI stLFR assemblies before and after gap-filling using ONT or PacBio data and after polishing with stLFR reads using one iteration of NextPolish.
Figure S3: BUSCO genome completeness assessment. (A) ONT assemblies before and after Illumina short-read polishing using one iteration of NextPolish (Flye, Canu, Raven, Redbean) and MaSuRCA hybrid assembly (B) PacBio assemblies using 32x or 84x sequencing coverage, (C) BGI stLFR assemblies before and after gap-filling using ONT or PacBio data and after polishing using stLFR reads and one iteration of NextPolish.
Figure S4: K-mer spectra plots from the k-mer Analysis Toolkit comparing the K-mers found in Illumina reads to the K-mers found in ONT, PacBio, stLFR and Illumina assemblies.

List of abbreviations

AUD: Australian dollars; bp: base pairs; BGI: Beijing Genomics Institute; BUSCO: Benchmarking Universal Single-Copy Orthologs; BWA: Burrows-Wheeler Aligner; g: gram; Gb: gigabase pairs; kb: kilobase pairs; Mb: megabase pairs; mg: milligram; µl: microlitre; ml: millilitre; mm: millimeter; ng: nanogram; ONT: Oxford Nanopore Technologies; PacBio: Pacific Biosciences; QUAST: QUality ASsessment Tool; SMRT: single-molecule real-time; SPAdes: St. Petersburg genome assembler; stLFR: Single Tube Long Fragment Reads; TB: Terabyte; USD: United States Dollar.

Competing Interests

Employees of BGI, MGI, and Complete Genomics have stock holdings in BGI.

Funding

This work was funded by the Genome Innovation Hub, Office of Research Infrastructure, The University of Queensland. This work was supported in part by the Shenzhen Peacock Plan (NO.KQTD20150330171505310).

Author's Contributions

A.F. prepared the sample. B.T. supervised plant collection. S.K.R. performed ONT library preparation and sequencing. T.J.C.B. performed PacBio library preparation and sequencing. V.M. performed ONT and PacBio assemblies and assembly evaluation. Q.Y. and H.W. performed stLFR library preparation and sequencing. I.H supervised and reviewed stLFR library preparation and sequencing. W.T. performed stLFR assembly, gap filling and statistics for stLFR. E.A., Q.M., R.D., O.W., and B.A.P. designed stLFR experiments and performed stLFR analyses. V.M. wrote the manuscript with input from all authors. R.J.H. and L.J.M.C. designed and supervised the project.

Acknowledgements

We acknowledge Doug Stetner and Thom Cuddihy for help with the Falcon software, Nicholas Rhodes and Chenxi Zhou for help with the MaSuRCA software, Tania Duarte for running the DNA sample in tapestation, Mobashwer Alam for provision of the Macadamia tissue samples.

References

1. Gross C, Weston P. *Macadamia jansenii* (Proteaceae), a new species from central Queensland. Australian Systematic Botany 1992;5(6):725–728.
2. The four macadamias;. Accessed February 14, 2020. http://www.wildmacadamias.org.au/the-four-macadamias.
3. Chase MW. Relationships between the families of flowering plants. In: Henry RJ, (ed.), Plant Diversity and Evolution: Genotypic and Phenotypic Variation in Higher Plants. Wallingford, Oxfordshire, UK ; Cambridge, MA: CABI Pub; 2005.
4. Brozynska M, Furtado A, Henry RJ. Genomics of crop wild relatives: expanding the gene pool for crop improvement. Plant Biotechnology Journal 2016 Apr;14(4):1070–1085.
5. Abberton M, Batley J, Bentley A, Bryant J, Cai H, Cockram J, et al. Global agricultural intensification during climate change: a role for genomics. Plant Biotechnology Journal 2016 Apr;14(4):1095–1098.
6. Henry RJ. Innovations in plant genetics adapting agriculture to climate change. Current Opinion in Plant Biology 2019 Dec;13:1–6.
7. Niu YF, Li GH, Ni SB, He XY, Zheng C, Liu ZY, et al. Genome assembly and annotation of *Macadamia tetraphylla*. bioRxiv 2020 Mar;.
8. Nock CJ, Baten A, Mauleon R, Langdon KS, Topp B, Hardner C, et al. Chromosome-Scale Assembly and Annotation of the Macadamia Genome (*Macadamia integrifolia* HAES 741). G3: Genes|Genomes|Genetics 2020 Aug;p. g3.401326.2020.
9. Nock CJ, Baten A, Barkla BJ, Furtado A, Henry RJ, King GJ. Genome and transcriptome sequencing characterises the gene space of Macadamia integrifolia (Proteaceae). BMC Genomics 2016 Dec;17(1):937.
10. Paajanen P, Kettleborough G, López-Girona E, Giolai M, Heavens D, Baker D, et al. A critical comparison of technologies for a plant genome sequencing project. GigaScience 2019;8(3).
11. Belser C, Istace B, Denis E, Dubarry M, Baurens FC, Falentin C, et al. Chromosome-scale assemblies of plant genomes using nanopore long reads and optical maps. Nature Plants 2018 Nov;4(11):879–887.
12. Logsdon GA, Vollger MR, Eichler EE. Long-read human genome sequencing and its applications. Nature Reviews Genetics 2020 Jun;.
13. Jung H, Winefield C, Bombarely A, Prentis P, Waterhouse P. Tools and Strategies for Long-Read Sequencing and De Novo Assembly of Plant Genomes. Trends in Plant Science 2019 Aug;24(8):700–724.
14. Wang O, Chin R, Cheng X, Wu MKY, Mao Q, Tang J, et al. Efficient and unique cobarcoding of second-generation sequencing reads from long DNA molecules enabling cost-effective and accurate sequencing, haplotyping, and de novo assembly. Genome Research 2019 May;29(5):798–808.
15. Drmanac R, Nucleic Acid Analysis by Random Mixtures of Non-Overlapping Fragments. Patent WO 2006/138284; 2006.
16. Peters BA, Liu J, Drmanac R. Co-barcoded sequence reads from long DNA fragments: a cost-effective solution for "perfect genome" sequencing. Frontiers in Genetics 2014;5:466.
17. Furtado A. DNA extraction from vegetative tissue for next-generation sequencing. Methods in Molecular Biology (Clifton, NJ) 2014;1099:1–5.
18. Wang L, Xi Y, Zhang W, Wang W, Shen H, Wang X, et al. 3' Branch ligation: a novel method to ligate non-complementary DNA to recessed or internal 3'OH ends in

DNA or RNA. DNA research: an international journal for rapid publication of reports on genes and genomes 2019 Feb;26(1):45–53.
19. De Coster W, D'Hert S, Schultz DT, Cruts M, Van Broeckhoven C. NanoPack: visualizing and processing long-read sequencing data. Bioinformatics 2018 Aug;34(15):2666–2669.
20. Wick R, Porechop: adapter trimmer for Oxford Nanopore reads;. https://github.com/rrwick/Porechop, accessed May 23, 2019.
21. Wick R, Filtlong: quality filtering tool for long reads;. https://github.com/rrwick/Filtlong, accessed May 23, 2019.
22. Hall MB, Rasusa: Randomly subsample sequencing reads to a specified coverage. Zenodo; 2019. https://doi.org/10.5281/zenodo.3546168.
23. Bolger AM, Lohse M, Usadel B. Trimmomatic: a flexible trimmer for Illumina sequence data. Bioinformatics (Oxford, England) 2014 Aug;30(15):2114–2120.
24. Chen S, Zhou Y, Chen Y, Gu J. fastp: an ultra-fast all-in-one FASTQ preprocessor. Bioinformatics (Oxford, England) 2018;34(17):i884–i890.
25. Marçais G, Kingsford C. A fast, lock-free approach for efficient parallel counting of occurrences of k-mers. Bioinformatics 2011 Mar;27(6):764–770.
26. Vurture GW, Sedlazeck FJ, Nattestad M, Underwood CJ, Fang H, Gurtowski J, et al. GenomeScope: fast reference-free genome profiling from short reads. Bioinformatics 2017 Jul;33(14):2202–2204.
27. Ruan J, Li H. Fast and accurate long-read assembly with wtdbg2. Nature Methods 2020 Feb;17(2):155–158.
28. Kolmogorov M, Yuan J, Lin Y, Pevzner PA. Assembly of long, error-prone reads using repeat graphs. Nature Biotechnology 2019;37(5):540–546.
29. Koren S, Walenz BP, Berlin K, Miller JR, Bergman NH, Phillippy AM. Canu: scalable and accurate long-read assembly via adaptive *k* -mer weighting and repeat separation. Genome Research 2017 May;27(5):722–736.
30. Vaser R, Šikić M. Raven: a de novo genome assembler for long reads 2020 Aug;.
31. Chagné D. Whole genome sequencing of fruit tree species. In: Advances in Botanical Research, vol. 74. Elsevier; 2015.
32. Vaser R, Sović I, Nagarajan N, Šikić M. Fast and accurate de novo genome assembly from long uncorrected reads. Genome Research 2017 May;27(5):737–746.
33. Li H. Minimap2: pairwise alignment for nucleotide sequences. Bioinformatics 2018 Sep;34(18):3094–3100.
34. medaka: Sequence correction provided by ONT Research;. https://github.com/nanoporetech/medaka, accessed September 5, 2019.
35. Walker BJ, Abeel T, Shea T, Priest M, Abouelliel A, Sakthikumar S, et al. Pilon: An Integrated Tool for Comprehensive Microbial Variant Detection and Genome Assembly Improvement. PLoS ONE 2014 Nov;9(11):e112963.
36. Li H. Aligning sequence reads, clone sequences and assembly contigs with BWA-MEM. arXiv:13033997 [q-bio] 2013 May;ArXiv: 1303.3997.
37. Hu J, Fan J, Sun Z, Liu S. NextPolish: a fast and efficient genome polishing tool for long read assembly. Bioinformatics (Oxford, England) 2019 Nov;.
38. Zimin AV, Marçais G, Puiu D, Roberts M, Salzberg SL, Yorke JA. The MaSuRCA genome assembler. Bioinformatics (Oxford, England) 2013 Nov;29(21):2669–2677.
39. Chin CS, Peluso P, Sedlazeck FJ, Nattestad M, Concepcion GT, Clum A, et al. Phased diploid genome assembly with single-molecule real-time sequencing. Nature Methods 2016 Dec;13(12):1050–1054.
40. Roach MJ, Schmidt SA, Borneman AR. Purge Haplotigs: allelic contig reassignment for third-gen diploid genome assemblies. BMC bioinformatics 2018 Nov;19(1):460.
41. Bankevich A, Nurk S, Antipov D, Gurevich AA, Dvorkin M, Kulikov AS, et al. SPAdes: a new genome assembly algorithm and its applications to single-cell sequencing. Journal of Computational Biology: A Journal of Computational Molecular Cell Biology 2012 May;19(5):455–477.
42. Superplus split_barcode;. Accessed August 19, 2019. https://github.com/MGI-tech-bioinformatics/SuperPlus/blob/master/split_barcode/split_barcode_PEXXX_42_unsort_reads.pl.
43. Martin M. Cutadapt removes adapter sequences from high-throughput sequencing reads. EMBnetjournal 2011 May;17(1):10.
44. Weisenfeld NI, Kumar V, Shah P, Church DM, Jaffe DB. Direct determination of diploid genome sequences. Genome Research 2017 May;27(5):757–767.
45. Xu M, Guo L, Gu S, Wang O, Zhang R, Peters BA, et al. TGS-GapCloser: A fast and accurate gap closer for large genomes with low coverage of error-prone long reads. GigaScience 2020 Sep;9(9):giaa094.
46. Lidong, Xu M, BGI-Qingdao/TGSGapFiller. Zenodo; 2020. https://doi.org/10.5281/zenodo.3755272.
47. Gurevich A, Saveliev V, Vyahhi N, Tesler G. QUAST: quality assessment tool for genome assemblies. Bioinformatics 2013 Apr;29(8):1072–1075.
48. Simão FA, Waterhouse RM, Ioannidis P, Kriventseva EV, Zdobnov EM. BUSCO: assessing genome assembly and annotation completeness with single-copy orthologs. Bioinformatics 2015 Oct;31(19):3210–3212.
49. Mapleson D, Garcia Accinelli G, Kettleborough G, Wright J, Clavijo BJ. KAT: a K-mer analysis toolkit to quality control NGS datasets and genome assemblies. Bioinformatics 2016 Oct;33(4).
50. Guiglielmoni N, Derzelle A, van Doninck K, Flot JF. Overcoming uncollapsed haplotypes in long-read assemblies of non-model organisms. bioRxiv; 2020.
51. Alam MM, Wilkie J, Topp BL. Early growth and graft success in macadamia seedling and cutting rootstocks. Acta Horticulturae 2018 Jun;(1205):637–644.
52. Topp BL, Nock CJ, Hardner CM, Alam M, O'Connor KM. Macadamia (Macadamia spp.) Breeding. In: Al-Khayri JM, Jain SM, Johnson DV, editors. Advances in Plant Breeding Strategies: Nut and Beverage Crops Cham: Springer International Publishing; 2019.p. 221–251.
53. Loose M, Malla S, Stout M. Real-time selective sequencing using nanopore technology. Nature Methods 2016 Sep;13(9):751–754.
54. Kingan S, Heaton H, Cudini J, Lambert C, Baybayan P, Galvin B, et al. A High-Quality De novo Genome Assembly from a Single Mosquito Using PacBio Sequencing. Genes 2019 Jan;10(1):62.
55. Ghurye J, Pop M. Modern technologies and algorithms for scaffolding assembled genomes. PLoS computational biology 2019;15(6):e1006994.

Figure 1

Click here to access/download;Figure;Fig1_N5

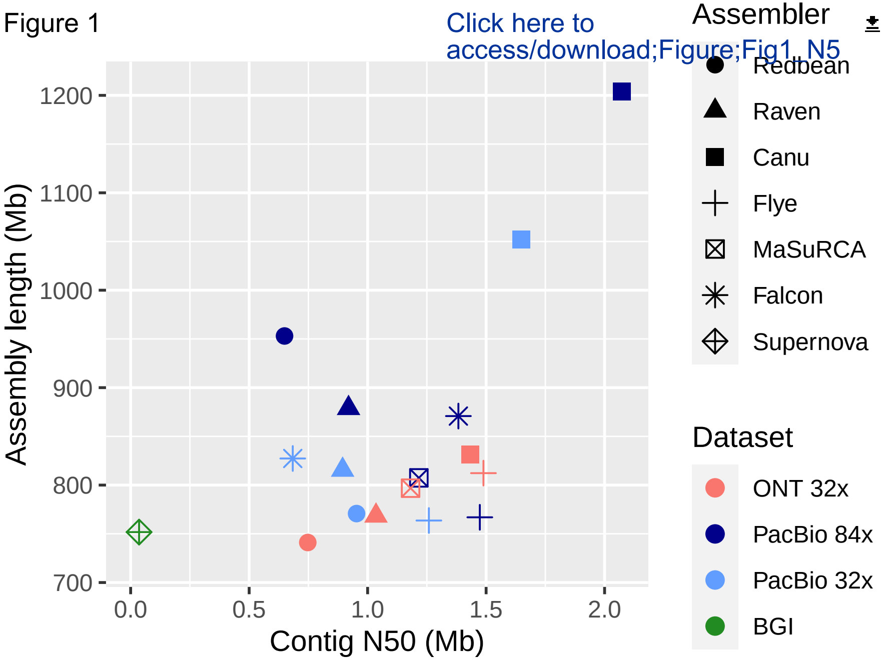

Figure 2

Click here to access/download;Figure;Fig2_accuracy.pdf

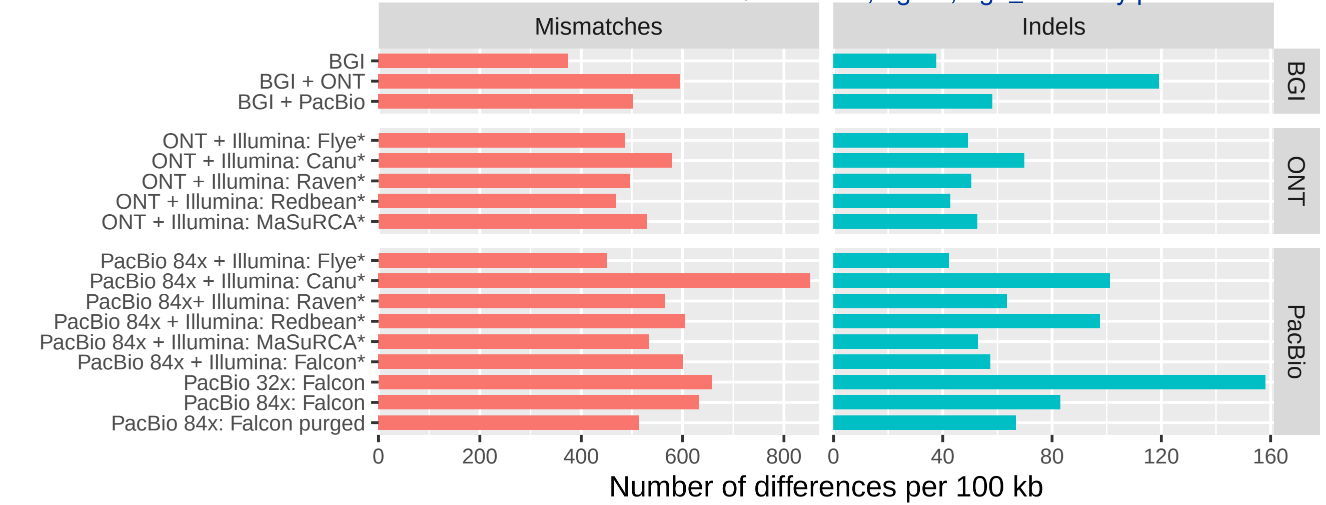

Figure 3

Click here to access/download;Figure;Fig3_BUSCO.pdf

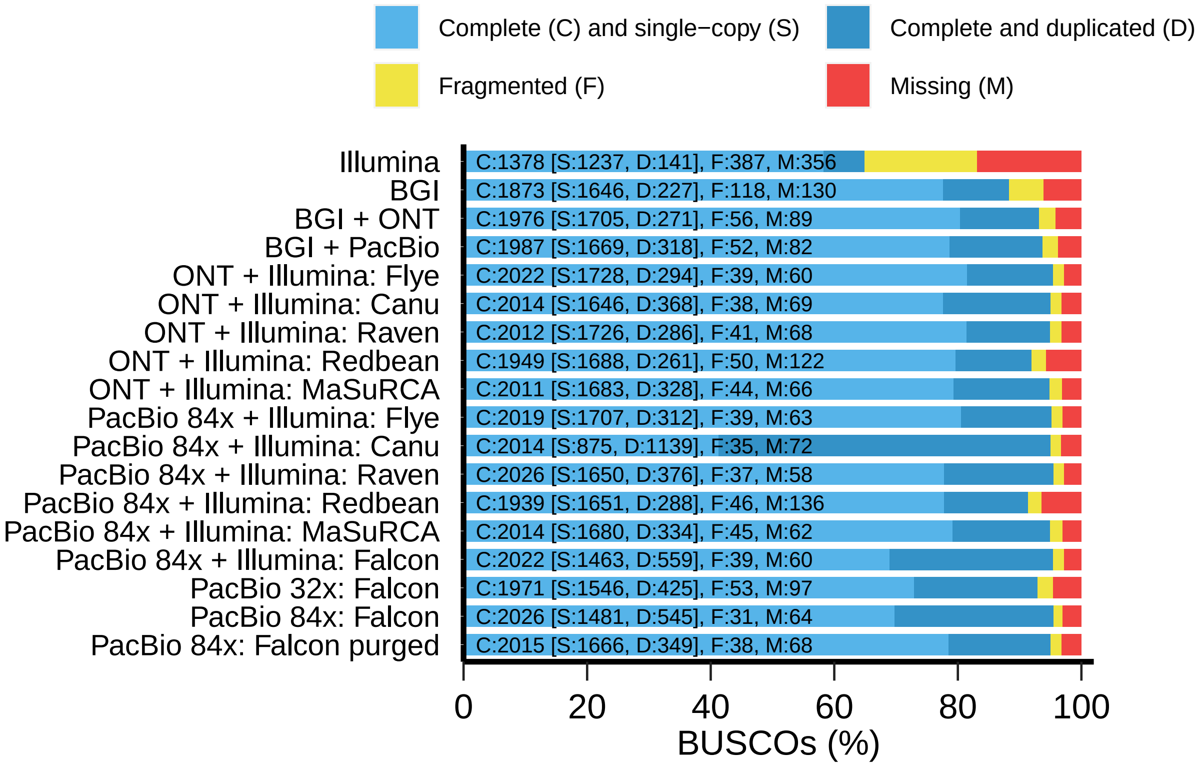

Supplementary Figures

Click here to access/download
Supplementary Material
Murigneux_et_al_SupFigures_revision2.pdf

Supplementary Tables

Click here to access/download
Supplementary Material
Murigneux_et_al_SupTable_revision2.xlsx

Cover letter
Click here to access/download;Personal Cover;Cover_Letter_resubmission.pdf

Author’s cover letter for resubmission

Hongling Zhou
GigaScience

Brisbane, 28th September 2020

Dear Hongling Zhou,

Please find attached our revised manuscript entitled "Comparison of long read methods for sequencing and assembly of a plant genome" (GIGA-D-20-00077).

We would like to thank the editorial staff and the reviewers for their time and valuable comments. The revised manuscript includes corrections, updated figures and additional data to address the comments raised by the reviewer #2. The supplementary table S11 reports the estimated computational costs to generate the assemblies.

We hope that the revised manuscript will be suitable for publication as a research article in GigaScience. We believe that our benchmarking of long-read sequencing technologies and assembly algorithms will be of interest to the community.

The manuscript has been approved by all listed authors. All the sequencing data generated in this study have been deposited in the Sequence Read Archive database under BioProject PRJNA609013.

We look forward to hearing from you.

Yours faithfully,

Valentine Murigneux

Bioinformatician
Genome Innovation Hub
The University of Queensland
Brisbane QLD 4072 Australia

v.murigneux@uq.edu.au
