# Supplementary material for: Comparison of long-read methods for sequencing and assembly of a plant genome
Source: Gigascience. 2020 Dec 21;9(12):giaa146. doi: 10.1093/gigascience/giaa146 (PMC7751402; doi:10.1093/gigascience/giaa146)
Supplement: giaa146_Supplemental_Files [file giaa146_supplemental_files.zip › Murigneux_et_al_SupFigures_revision2.pdf]

A

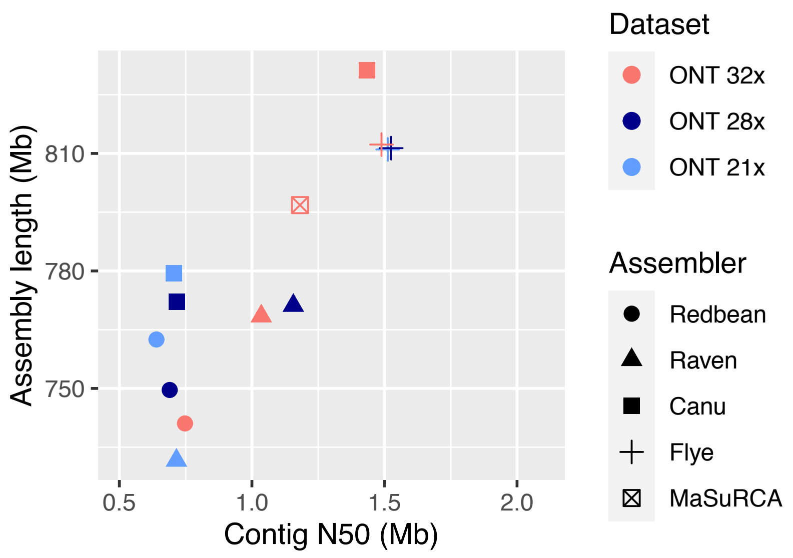

B

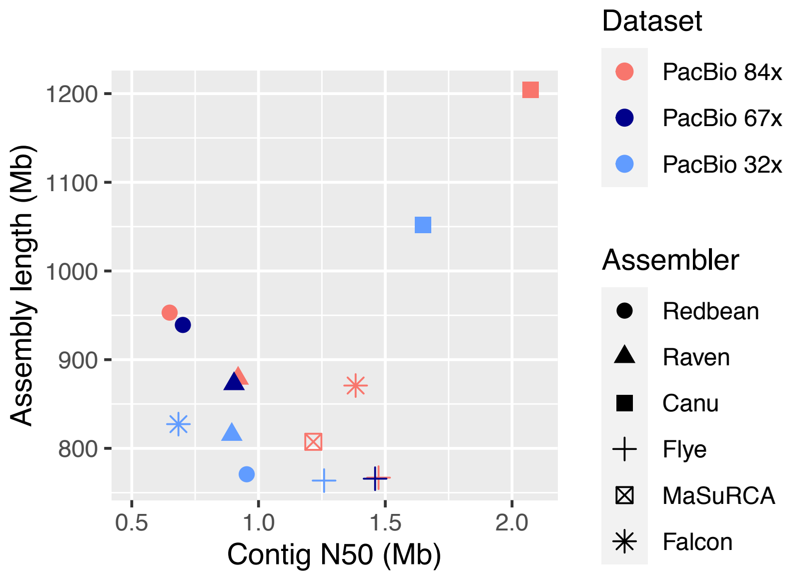

Figure S1: Genome assembly statistics
The total assembly length is plotted against the contig N50 for each assembler and sequencing coverage. (A) ONT assemblies, (B) PacBio assemblies.

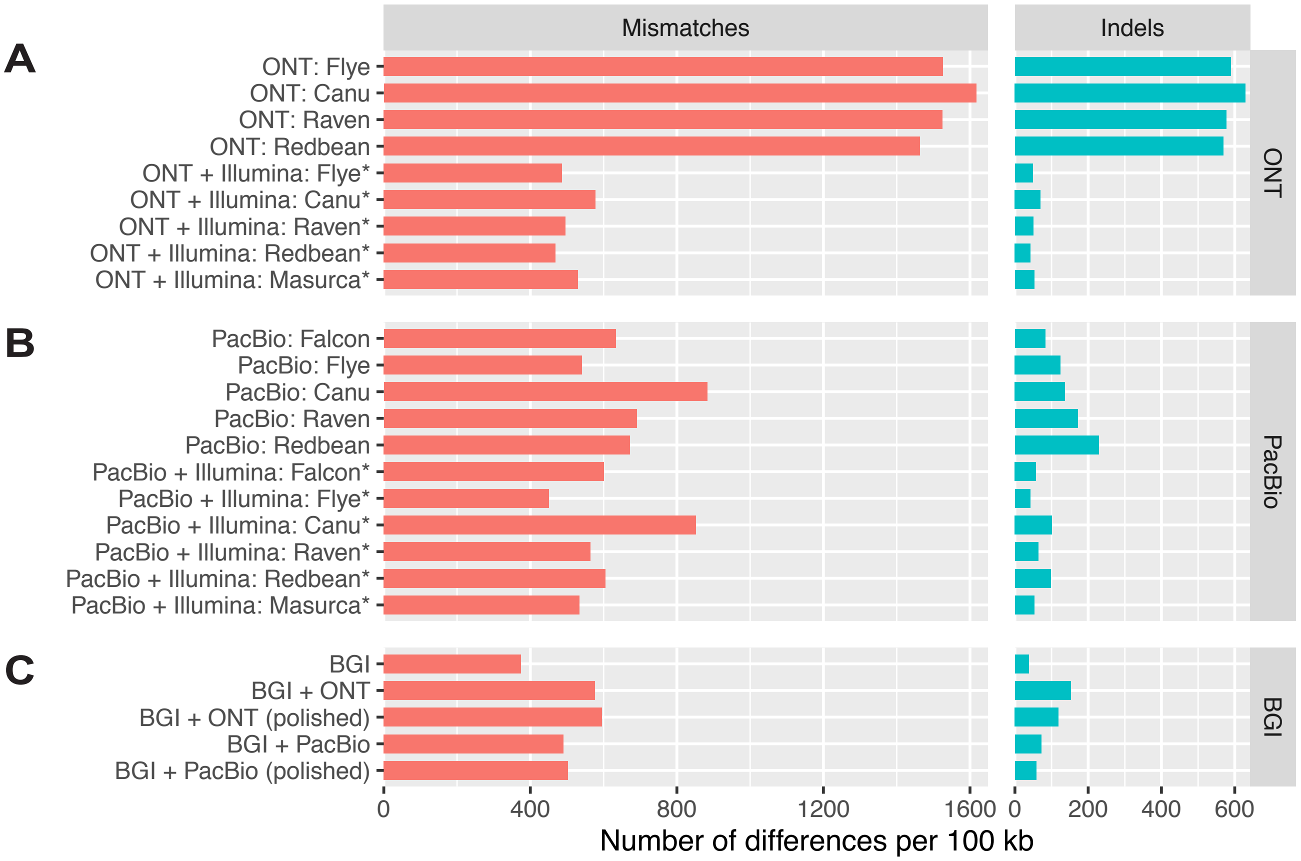

Figure S2: Number of mismatches and indels identified in the long-read assemblies as compared to the Illumina short-read assembly generated by SPAdes.
(A) ONT assemblies before and after Illumina short-read polishing using one iteration of NextPolish (Flye, Canu, Raven, Redbean) and MaSuRCA hybrid assembly, (B) PacBio assemblies before and after Illumina short-read polishing using one iteration of NextPolish (Falcon, Flye, Canu, Raven, Redbean) and MaSuRCA hybrid assembly, (C) BGI stLFR assemblies before and after gap-filling using ONT or PacBio data and after polishing with stLFR reads using one iteration of NextPolish.

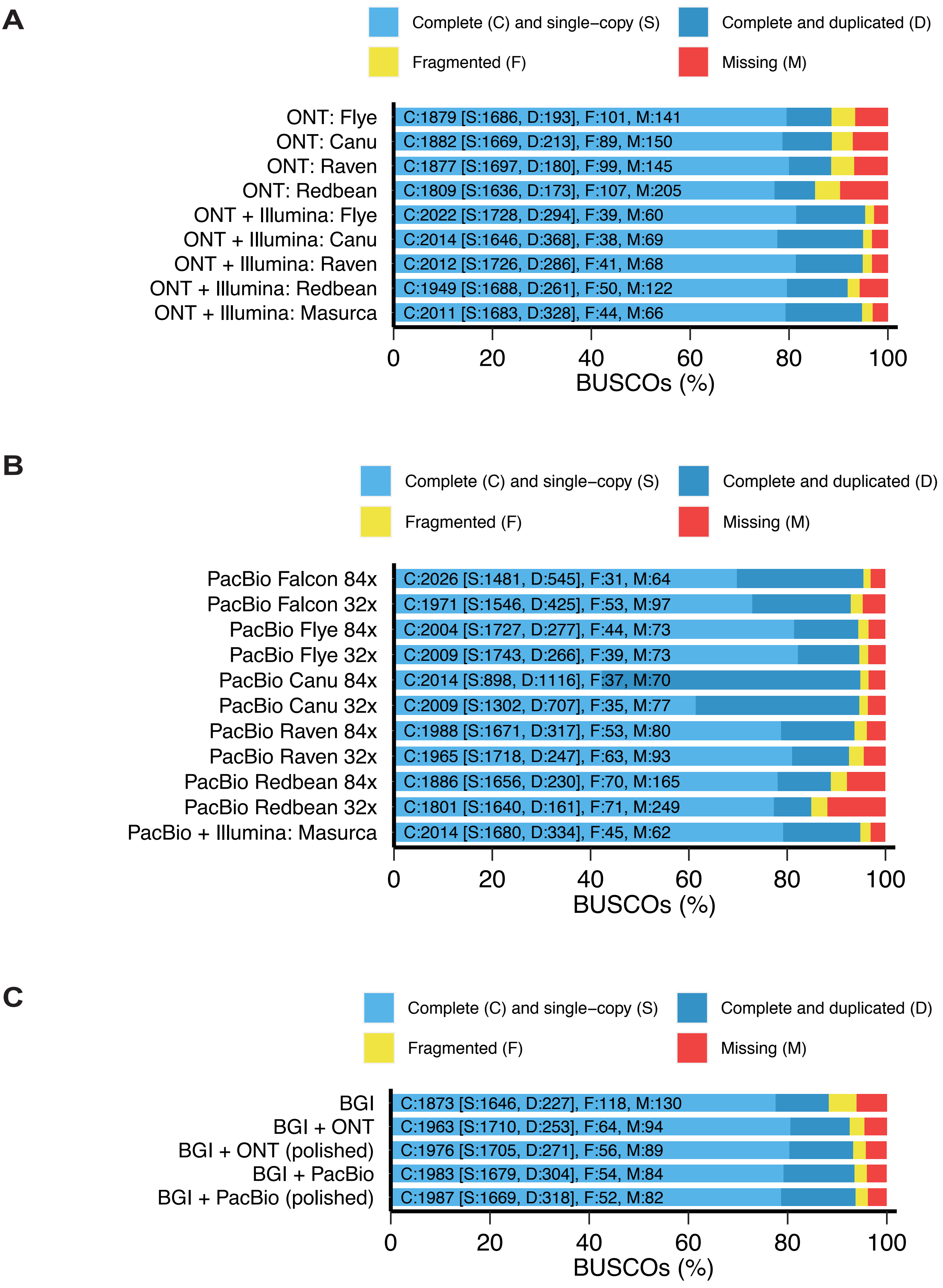

Figure S3: BUSCO genome completeness assessment
(A) ONT assemblies before and after Illumina short-read polishing using one iteration of NextPolish (Flye, Canu, Raven, Redbean) and MaSuRCA hybrid assembly, (B) PacBio assemblies using 32x or 84x sequencing coverage, (C) BGI stLFR assemblies before and after gap-filling using ONT or PacBio data and after polishing using stLFR reads and one iteration of NextPolish.

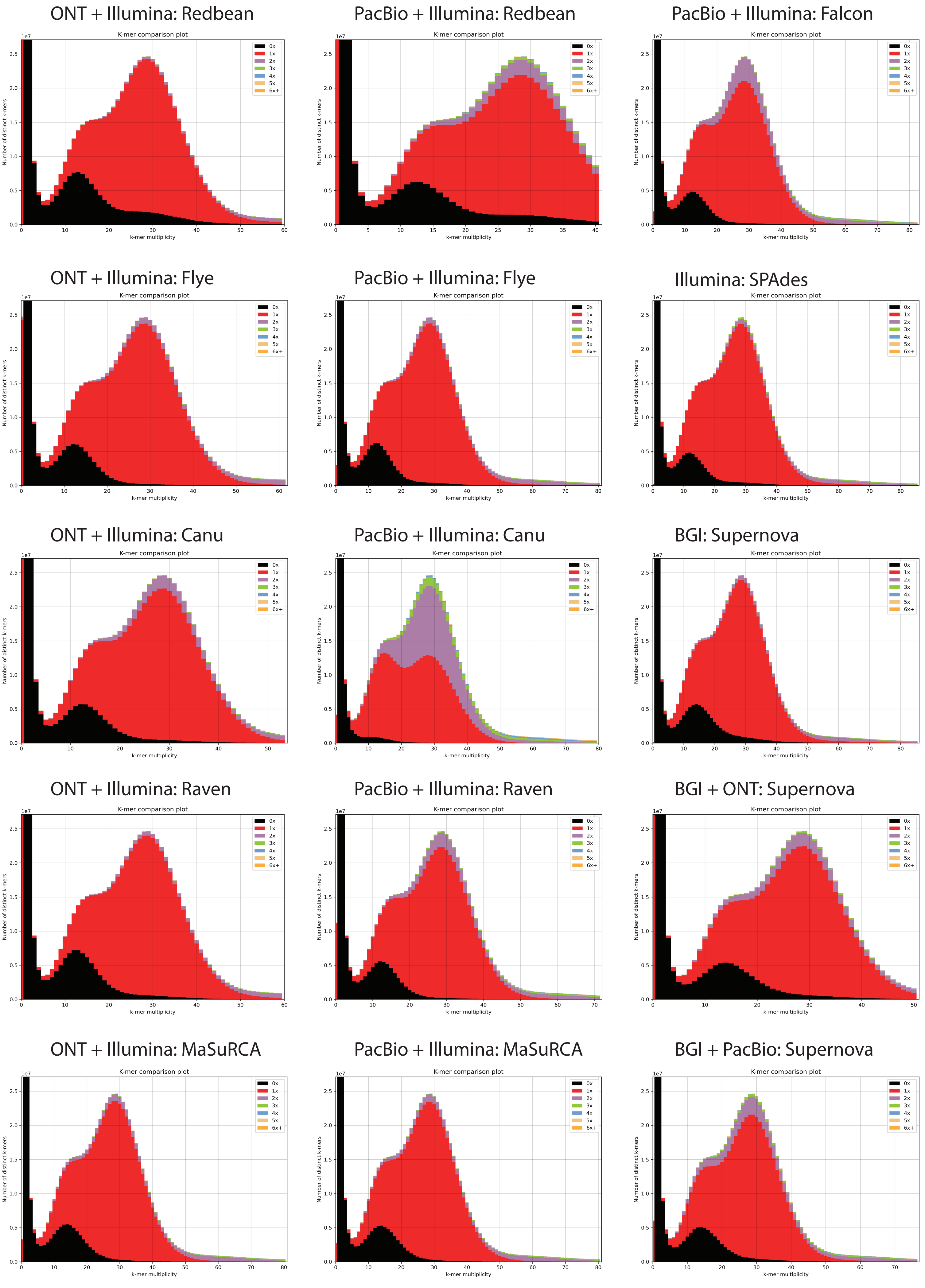

Figure S4: K-mer spectra plots from the k-mer Analysis Toolkit comparing the K-mers found in Illumina reads to the K-mers found in ONT, PacBio, stLFR and Illumina assemblies.
