# Supplementary material for: Comparison of long-read methods for sequencing and assembly of a plant genome
Source: Gigascience. 2020 Dec 21;9(12):giaa146. doi: 10.1093/gigascience/giaa146 (PMC7751402; doi:10.1093/gigascience/giaa146)
Supplement: giaa146_Response_to_Reviewer_Comments_Original_Submission [file giaa146_response_to_reviewer_comments_original_submission.pdf]

Author's Response To Reviewer Comments

Close

GIGA-D-20-00077
Comparison of long read methods for sequencing and assembly of a plant genome

We would like to thank the editorial staff and the reviewers for their time and valuable comments. We performed additional analyses to address the comments and clarified specific points raised by the reviewers. We believe our manuscript has improved through the clarifications and the additional data. We have included a point-by-point replies to the reviewer's comments below, highlighted in purple. The modifications in the revised manuscript have been highlighted in yellow.

Reviewer #1: Introduction part:
- It would be nice to put the genome size and to indicate the reference genome that is already sequenced and assembled for Macadamia, just to put a context for the people who are not familiar with Macadamia.
We thank the reviewer for the suggestion. We have added a paragraph in the introduction to provide some information about the already sequenced and assembled Macadamia genomes.
The macadamia genus contains four species: Macadamia integrifolia, Macadamia tetraphylla, Macadamia ternifolia and Macadamia jansenii. Macadamia cultivars are diploid (2n = 28) with k-mer based genome size estimates ranging from 758 Mb for M. tetraphylla [7] to 896 Mb for M. integrifolia [8]. The first draft genome assembly of the widely grown Macadamia integrifolia cultivar HAES 741 was constructed from short-read Illumina sequence data and was highly fragmented (518Mb, 193,493 scaffolds, N50 = 4,745 bp) [9]. An improved HAES 741 assembly was generated using a combination of long-read PacBio and paired-end Illumina sequence data (745Mb, 4,094 scaffolds, N50 = 413 kb) [8]. The genome assembly of Macadamia tetraphylla was also recently produced using a combination of long-read ONT and short-read Illumina sequence data (751 Mb, 4,335 contigs, N50 = 1.18 Mb) [7].

Methods part:
- ONT library preparation and sequencing part:
- What was the reason to used both MinION and PromethION and not only PromethION?
The MinION run was performed to check the compatibility of the DNA sample with Nanopore sequencing as well as the quality of the library preparation and to estimate the sequence throughput in order to get enough genome coverage from the PromethION run.

- For what reason didn't you use the same version of MinKNOW to assemble the MinION (MinKNOW (v1.15.4)) and PromethION (MinKNOW (v3.1.23)) data?
The MinKNOW software has not been used to assemble the data, it is the software used to acquire the primary data (fast5 reads) from the sequencing device. The MinKNOW software version is machine specific therefore MinION and PromethION have their own version although the software has the same name and does the same job during the sequencing run. We used the same Guppy version 3.0.3 to basecall the raw signal data from both MinION and PromethION runs.

- Assembly of genomes part:
- Is there a reason for doing 4 iterations of Racon? And not 3 or 5?
We thank the reviewer for raising this point. We used Racon to polish the assembly as a first step before using the Medaka software. Therefore we followed the recommendations from the Medaka GitHub page to run 4 iterations of Racon before running Medaka: 'Medaka has been trained to correct draft sequences processed through racon, specifically racon run four times iteratively with: racon -m 8 -x -6 -g -8 -w 500 ...' (https://github.com/nanoporetech/medaka#origin-of-the-draft-sequence). For 4 out of 5 assemblers tested on the ONT data, the percentage of complete BUSCO genes is slightly higher after 4 iterations of Racon as compared to 1 iteration (Table S3).

- Maybe you should precise that Racon is used as an error-correction module and Medaka to create the consensus sequence.
We have amended the text :

For ONT data, four rounds of error correction were performed using Racon v1.4.9 (Racon,RRID:SCR_017642) [30] with recommended parameters (-m 8-x -6 -g -8 -w 500) based on minimap2 v2.17-r943-dirty [31] overlaps, followed by one round of Medaka v0.8.1 [32] using the r941_prom_high model to create the consensus sequence.

- "Hybrid assembly was generated with MaSuRCA v3.3.3 (MaSuRCA, RRID:SCR_010691) [32] using the Illumina and the ONT or PacBio reads and using Flye v2.5 to perform the final assembly of corrected mega-reads" this sentence is not very clear to me. Does it mean that you have first used ONT/PacBio data + Illumina on MaSuRCA software to generate what they call "super-reads" and then from this data you used Flye to get the final assemblies?
Yes, the MaSURCA v3.3.3 software includes a parameter (FLYE_ASSEMBLY=0) to choose which assembler to use for the final assembly of corrected mega-reads (CABOG or Flye). The authors recommend to use Flye as it is 'a lot faster than CABOG, and quality is the same or better' (https://github.com/alekseyzimin/masurca#configuration). We have amended the text to clarify this part:
Hybrid assembly was generated with MaSuRCA v3.3.3 (MaSuRCA, RRID:SCR_010691) using the Illumina and the ONT or PacBio reads and using Flye v2.5 to perform the final assembly of corrected mega-reads (parameter FLYE_ASSEMBLY=1).

- as I understood stLFR is similar to 10x genomics, why not compare this technology data too?
We thank the reviewer for the question. stLFR is similar in principle to the 10x Genomics technology but the Chromium Genome Sequencing products from the company have been discontinued as of June 30, 2020.

- Assembly comparison part:
- "We compared the assemblies with the published reference genome of Macadamia integrifolia v2 (Genbank accession: GCA_900631585.1)." First, I think it is important to add the reference paper. Secondly, I cannot see where did you compare your assemblies with the one published? For me, you compared all your assemblies between each other, but I cannot find any other assembly.
- We changed the title of this methods section to "Assembly evaluation" instead of "Assembly comparison" to better reflect its content (QUAST and BUSCO assembly metrics, accuracy estimation).
- The sentence mentioned referred to the QUAST analysis only. Some of the QUAST metrics reported in Table 2, Table S1, S2 and S4 requires a reference genome (contig NG50 values, misassemblies).
- We have amended the text to include the reference paper of Macadamia integrifolia v2 (Nock et al., bioRxiv, 2020) and clarify when the reference genome was used:
The publicly available reference genome of Macadamia integrifolia v2 (Genbank accession: GC_900631585.1) [8] was used as the reference genome for QUAST.

- when you said "Illumina assembly" do you refer to the Macadamia integrifolia assembly? If so, please clarify it in the rest of the paper, and add the data for this reference genome in your figures.
We apologised for the confusion. By Illumina assembly, we referred to the SPAdes assembly generated using the Illumina short reads. We have added a paragraph entitled "Illumina genome assembly" to present the results of the Illumina short read assembly. We also amended the text in the Methods section:
To estimate the base accuracy, QUAST was used to compute the number of mismatches and indels as compared to the Illumina short-read assembly generated by SPAdes.

Results part:
- ONT genome assembly part:
- Is there any interested to combine MinION and PromethION data? Are there any advantages to combining it?
We combined the MinION data (1.7Gb, ~2x coverage) and the PromethION data (23.2 Gb, ~30x coverage) in order to get more genome coverage for the final assembly.

- "The genome completeness was slightly better after two iterations of NextPolish (95.5%) than after two iterations of Pilon (95.2%) (Sup Table 1)." Here I would precise that it is the case for the Flye assembly, but surprisingly (at least for me?) after two iterations of NextPolish on the Canu assembly, the results were a little less good as with one iteration. So, depending on the assembler you use, the number of iteration needed might be different.
We thank the reviewer for this comment. We agree that it is a bit surprising even though the difference in the percentage of complete genes is very small (95% vs 94.8%). We have amended the text to reflect this observation:

The genome completeness was slightly better after two iterations of NextPolish than after two iterations of Pilon for the Flye (95.5% vs 95.2%) and Redbean assemblies (91.9% vs 91.6%) (Table S3). Pilon and NextPolish gave similar completeness results when applied to the Canu and Raven assemblies. A second iteration of Pilon resulted in a slight decrease in the number of missing genes and a higher accuracy for all four assemblers whereas a second iteration of NextPolish did not improve the genome completeness and accuracy (mismatches) for the Canu and Raven assemblies. Therefore, depending on the assembler and the polisher used, the number of recommended iterations might be different.

- "As an estimation of the base accuracy, we computed the number of mismatches and indels as compared to the Illumina assembly." Here I am not sure which assembly you refer to when you use the "Illumina assembly" term. Do you refer to the Macadamia integrifolia assembly or to the MaSuRCA hybrid assembly? If you refer to the last one, I would suggest using the word hybrid assembly instead of Illumina assembly, it might be confusing.
We apologised for the confusion here. By Illumina assembly, we referred to the SPAdes assembly generated using the Illumina short reads. We have added a paragraph entitled "Illumina genome assembly" to present the results of the Illumina short read assembly. We also amended the text in the Results section:
As an estimation of the base accuracy, we computed the number of mismatches and indels as compared to the Illumina short-read assembly generated by SPAdes.

- Why not using the Pilon and NextPolish step on the ONT+Illumina (MaSuRCA) assembly since they are tools dedicated to long and short reads polishing?
The super reads constructed by MaSuRCA (which are finally used to build the assembly) are based on the Illumina reads. Therefore it is unlikely that Illumina short-read polishing will significantly improve the assembly. To confirm this, we performed short read polishing (using Pilon or NextPolish) on the MaSuRCA assembly. BUSCO results (Table S3) confirmed that the polishing step did not improve the genome completeness: 94.8% complete BUSCOs (MaSuRCA only) as compared to 94.9% (MaSuRCA + Pilon) and 94.8% (MaSuRCA + NextPolish). We also performed long read polishing (using Racon and Medaka) followed by short read polishing on the MaSuRCA assembly. Again, BUSCO results showed that the polishing steps did not significantly improve the genome completeness: 94.8% complete BUSCOs (MaSuRCA only) as compared to 94.5% (MaSuRCA + Racon + Medaka + Pilon) and 95.0% (MaSuRCA + Racon + Medaka + NextPolish). The percentage of duplicated BUSCOs is slightly higher in the unpolished assembly (15.5%) as compared to the long-read and short-read polished assemblies (13.8%, Pilon) and (14.3%, NextPolish). We have added those results in the text of the manuscript:
Short-read polishing or long-read followed by short-read polishing did not significantly improve the genome completeness of the MaSuRCA assembly (Table S3), which is expected as the super-reads constructed by this tool are based on the Illumina reads.

- PacBio genome assembly part:
- Why did you use FALCON as the assembler for PacBio but not for ONT? If I am correct, it is not uniquely build to work on PacBio data but is ok for all long-reads technologies.
FALCON has been designed to take into account the specific characteristics of the PacBio data type. The ONT data contains more errors than the PacBio data. Therefore we think that it is not necessary to run FALCON on the ONT data. Furthermore, we have applied five 'generic' assemblers to both the ONT and PacBio data: Flye, Redbean, Canu, Raven, MaSuRCA (Raven assembly was also performed on the PacBio data and results are included in the revised manuscript).

- "Two subsets of reads corresponding to 4 SMRT cells and equivalent to a 43× and 39× coverage were assembled using Flye." why choosing Flye for this analysis? I'm also wondering if this part is necessary since afterward, you do the ONT equivalent coverage which is more interesting for the comparison of the technologies.
We thank the reviewer for this comment. We chose Flye for this analysis as it was one of the fastest assemblers. We agree with the reviewer that this part is not necessary since the conclusions from this analysis are similar to the ones obtained from the ONT equivalent coverage analysis. We have removed this paragraph from the text and the corresponding columns in the Table S4.

- Comment on the structure: for this paragraph, I would prefer to have first the result with the same assemblers as with the ONT data, and then an explanation of why you choose to perform also a test with FALCON and then the FALCON results.
We thank the reviewer for the suggestion and we have modified the structure of the paragraph.

- stLFR genome assembly part:

- Supernova might have been used on PacBio data as well, why not?
Supernova is a software package for de novo assembly from Chromium Linked-Reads that are made from a Chromium prepared library (https://support.10xgenomics.com/de-novo-assembly/software/overview/latest/welcome). It is a specialised software taking short read sequencing data as input and expecting fastq files containing barcoded reads. Therefore Supernova is not suitable to assemble long-read data such as PacBio data. We applied Supernova to stLFR data because stLFR generates barcoded short read data similar to the 10x Genomics linked-reads data.

- why not trying to complement PacBio data with stLFR as you did with ONT? Are there any incompatibilities?
We thank the reviewer for the suggestion. There are no incompatibilities to complement the stLFR assembly with PacBio data. We have now performed the analysis using the same gap-filling software TGS-GapCloser and we compared the results with those obtained with the ONT data in the Table 3 and Table S9. The text in the stLFR genome assembly results section has been modified to include those results as well.

Discussion part:
- "The amount of sequencing data produced by each platform corresponds to approximately 84× (PacBio Sequel), 32× (ONT) and 96× (BGI stLFR) coverage of the macadamia genome" I would have put this information into the Results part, but it's only my preference.
We have removed this sentence from the discussion as this information is already present in the respective ONT, stLFR and PacBio genome assembly sections in the Results part.

- "For both ONT and PacBio data, the highest assembly contiguity was obtained with a long-read only assembler as compared to an hybrid assembler incorporating both the short and long reads." I would suggest using the term "long-read polished" instead of "long-read only" since the assembly with the best contiguity integrates the Illumina data for the polishing.
We thank the reviewer for this comment and we have modified the sentence accordingly.

Tables and figures:
- Table 2:
- For this figure, if I understood properly you have chosen the best assembly of each technology. If I am right, then please precise it in the title of the figure.
We amended the legend of the figure to include an explanation of the criteria chosen to select the assembly presented in this figure for each technology.
One assembly per technology was selected to be included in this table. For ONT, the Flye assembly was the most contiguous and for PacBio, the Falcon assembly was highly contiguous and the most complete assembly.

-Figure 1:
- If I understood properly and here when you write "Base accuracy of assemblies as compared to Illumina assembly" you refer to the Macadamia integrifolia assembly, then I would add the Macadamia integrifolia assembly in this figure, and maybe put a dotted line at the limit of it for each category (InDels and mismatches) so it is easier for the reader to compare with it.
We apologised for the confusion here again. By Illumina assembly, we referred to the SPAdes assembly generated using the Illumina short reads. We have modified the title of this figure: " Number of mismatches and indels identified in the long-read assemblies as compared to the Illumina short-read assembly generated by SPAdes". This figure represents the number of indels and mismatches identified in each long-read assembly as compared to the short-read assembly. We think that we should not include M. integrifolia in this figure in order to assess the base accuracy. M. jansenii and M. integrifolia are different species therefore we expect to see polymorphisms between them and those polymorphisms will be mixed with the base errors.

- Figure 2:
- Here I would put all the assemblies you had in Figure 1
We have updated the Figure 2 (now Figure 3) to include all the assemblies presented in Figure 1 (now Figure 2) with the exception of the Illumina assembly because it is used as a reference genome in the analysis presented in Figure 2 (now Figure 3).

Reviewer #2: In their paper Murigneux et. al. made a comparison of three long-read sequencing technologies applied to the de novo assembly of a plant genome, Macadamia jansenii. They generated sequencing data using Pacific Biosciences (Sequel I), Oxford Nanopore Technologies (PromethION), and

BGI (single-tube Long Fragment Read) technologies. Sequenced data are assembled using a bunch of state of the art long-read assemblers and hybrid Masurca assembler.
Although paper is easy to follow, and this kind of analysis is more than welcomed I have several major and minor concerns.

Major concerns

1) The authors use 780 Mbps as the estimated size of the genome. Yet, this is not supported by data. In chapter "Genome size estimation", they present the genome size estimation using K-mer counting, but these sizes are 650 Mbps or less
The genome size of Macadamia jansenii is unknown. There are four different Macadamia species and only two of them have been sequenced and assembled so far:
- M. integrifolia (Nock et al, bioRxiv, 2020): assembly size = 745 Mb , k-mer estimate = 896 Mb
- M. tetraphylla (Niu et al, bioRxiv, 2020): assembly size = 751 Mb , k-mer estimate = 758 Mb, flow cytometry estimate = 740 Mb
We used 780 Mb as the estimated genome size for M. jansenii because this value has been reported previously as the estimated genome size of M. integrifolia (Chagné
D, Advances in botanical research, 2015). We agree that the k-mer estimate could have been chosen for the genome size. It is interesting to see that Raven (the only assembler who does not require a genome size estimation as an input parameter) produced assemblies of around 770 -880 Mb.

2) Since the real size of the genome is unknown, It would be worthwhile if authors provide analyses such as those enabled by KAT (Mapleson et al., 2017), which compares the k-mer spectrum of the assembly to the k-mer spectrum of reads (preferably Illumina). For control of the misassembled contigs, authors also might align larger contigs obtained using different tools to compare similarity among them (e.g., using tools such as Gepard or similar).
-We thank the reviewer for the suggestion. We used KAT to compare the k-mer spectrum of the reads to the k-mer spectrum of the Illumina and stLFR reads. The results are included in the revised manuscript (Table S8 and Fig S4) and incorporated in the text.

3) The authors compare assemblies with "Illumina assembly", but it is not clear what that means and why they consider this as a valid comparison.
- We apologised for the confusion here. By Illumina assembly, we referred to the SPAdes assembly generated using the Illumina short reads. We have added a paragraph entitled "Illumina genome assembly" to present the results of the Illumina short read assembly. We also amended the text in the Methods and Results sections:
To estimate the base accuracy, QUAST was used to compute the number of mismatches and indels as compared to the Illumina short-read assembly generated by SPAdes.
- The Illumina sequencing library was prepared using the same DNA sample as the one used to prepare the libraries for the three long-read sequencing technologies. As we dont have a reference genome for the Macadamia jansenii species, we believe that comparing the long-read assemblies to the short-read assembly can be a means to assess the base accuracy of the long-read assemblies. The following paragraph has been added to the methods section to explain why we consider this as a valid comparison and mention about its associated limitations: "The Illumina short read assembly was generated using more accurate short reads as compared to long reads therefore it contained fewer base errors. Consequently the number of mismatches and indels identified in the long-read assemblies as compared to the short-read assembly will reflect their base error rates. We noted that this would only enable comparison to X% of the genome since the Illumina only assembly is relatively incomplete. Furthermore the Illumina assembly would be expected to have errors and those errors would result in calling errors in other assemblies even when they are actually correct".

4) Although they started ONT data analysis with four tools, they perform further analysis on just two tools (Flye and Canu). In addition, for PacBio data, they use three tools (Redbean, Fly and Canu). It is not clear why the authors chose these tools. Canu and Fly have larger N50, larger total length, and the longest contigs. However, this does not take into account possible misassembles. Assemblers might have problems with uncollapsed haplotypes, which can result in assemblies larger than expected. In their recent manuscript, Guiglielmoni et al (https://doi.org/10.1101/2020.03.16.993428) showed that Canu is prone to uncollapsed haplotypes. Also, in this manuscript is presented that using PacBio data Canu produces much longer assemblies than other tools (1.2 Gbps). Therefore, the longer total size of a assembly cannot guaranty a better genome. Furthermore, on ONT data Raven has the second-best initial Busco score (before polishing), and its assembled genome consists of the
least number of contigs. Therefore, I deem that the full analysis needs to performed using all tools for

both Nanopore and Pacbio data.
We thank the reviewer for this comment and mentioning the Guiglielmoni et al paper.
- The revised manuscript now includes the Raven assembly results for the PacBio data (Table S4). We updated the version of Raven from v0.0.0 to v1.1.6 for the ONT data and modified the text in the Results section accordingly.
- We performed long-read and short-read polishing on the Canu, Flye, Redbean and Raven assemblies for both the ONT and PacBio data and updated the corresponding supplementary tables 2, 3, 4 and 6. We have added the supplementary figure S2 to report the BUSCO completeness results for all the assemblers.
- We agree with the reviewer that a longer assembly size or larger N50 cannot guarantee a better genome assembly. The Canu assembly likely contains uncollapsed haplotypes as suggested by the high level of duplication estimated from BUSCO and QUAST as well as the k-mer estimated assembly completeness . We include a citation of the Guiglielmoni et al manuscript to inform the reader about the similar observation from this study. We also noted that the PacBio Canu assembly likely contains a higher number of misassemblies as compared to the other assemblies (Table S4, QUAST analysis as compared to the reference genome of M. integrifolia). We have amended the text in the PacBio genome assembly section.
The Canu assembly was the largest (1.2 Gb) but contained a higher fraction of duplication as reported by QUAST (1.64) and confirmed by the percentage of duplicated BUSCOs (53%) and the k-mer spectra (Fig S4). Therefore, the Canu assembly likely contains uncollapsed haplotypes corresponding to artefactually duplicated regions, as reported recently \citep{guiglielmoni_2020}. Aligning the PacBio assemblies to the Macadamia integrifolia assembly identified a higher number of misassemblies in the Canu asssembly (n = 38,800) as compared to the other assemblies (n = 21,000-27,000).
- We replaced the verb "improve" by the verb "increase" in the sentences below to remove the idea that a higher contiguity value implies a better assembly.
It is worth noting that Flye consistently produced assemblies of around 812 Mb with a contig N50 of approximately 1.5 Mb whereas Canu, Redbean and Raven assembly contiguity increased as the read coverage increased. In particular, the Canu contig N50 significantly increased from 706 kb (21×) to 1.43 Mb (32×).

5) It would be of interest to a broad community if authors add the computational costs in total cost per genome for each sequencing technolgy. They might compare their machines with AWS or other cloud specified configurations. Besides, it is not clear which types of machines they used. Information from supplementary materials such as GPU, large memory, HPC is not descriptive enough.
We have added the name of the computing cluster used for each assembly and the technical specifications of the different computing clusters in the Table S10.

Minor comments:

1) The authors use the published reference genome of Macadamia integrifolia v2 for comparison. It would be interesting if they can provide us with information about sequencing read technology used for this assembly.
We thank the reviewer for the suggestion. We have added a paragraph in the introduction to provide some information about the already sequenced and assembled Macadamia genomes.
The macadamia genus contains four species: Macadamia integrifolia, Macadamia tetraphylla, Macadamia ternifolia and Macadamia jansenii. Macadamia cultivars are diploid (2n = 28) with k-mer based genome size estimates ranging from 758 Mb for M. tetraphylla [7] to 896 Mb for M. integrifolia [8]. The first draft genome assembly of the widely grown Macadamia integrifolia cultivar HAES 741 was constructed from short-read Illumina sequence data and was highly fragmented (518Mb, 193,493 scaffolds, N50 = 4,745 bp) [9]. An improved HAES 741 assembly was generated using a combination of long-read PacBio and paired-end Illumina sequence data (745Mb, 4,094 scaffolds, N50 = 413 kb) [8]. The genome assembly of Macadamia tetraphylla was also recently produced using a combination of long-read ONT and short-read Illumina sequence data (751 Mb, 4,335 contigs, N50 = 1.18 Mb) [7].

2) The authors mentioned that the newer generation of PacBio sequencing technology (Sequel II) which provides higher accuracy and lower costs. It would also be worth to mention the newer generations of assembly tools such as Canu 2.0, Raven v1.1.5 or Flye Version 2.7.1
We thank the reviewer for this comment and we have modified the text accordingly.

It is worth considering Racon for polishing with Illumina reads too. Yet, this is not a requirement, because authors already use state of the art tools.

Close
