# Supplementary material for: Comparison of long-read methods for sequencing and assembly of a plant genome
Source: Gigascience. 2020 Dec 21;9(12):giaa146. doi: 10.1093/gigascience/giaa146 (PMC7751402; doi:10.1093/gigascience/giaa146)
Supplement: giaa146_Response_to_Reviewer_Comments_Revision_1 [file giaa146_response_to_reviewer_comments_revision_1.pdf]

Author's Response To Reviewer Comments

Close

GIGA-D-20-00077
Comparison of long read methods for sequencing and assembly of a plant genome

We would like to thank the editorial staff and the reviewers for their time and valuable comments. The revised manuscript includes corrections, updated figures and additional data to address the comments raised by the reviewer #2. The supplementary table S11 reports the estimated computational costs to generate the assemblies. We believe our manuscript has improved through the corrections and the additional information provided. We have included a point-by-point replies to the reviewer's comments below, highlighted in purple. In the revised manuscript (Murigneux_et_al_revision2.pdf), the modifications are highlighted in yellow.

Reviewer reports:

Reviewer #1: I would like to thank the authors for their responses clarifying my interrogation and for their corrections and improvements to the manuscript.
I believe the manuscript has much improved through the clarifications and newly added data to complete the analysis.
I do not have any other comments on this work, and I would accept it to be published.
We thank the reviewer for this positive feedback.

Reviewer #2: The authors have answered most of the comments from my previous review adequately.
My remaining concern is mostly related to the following paragraph:
"The cost of generating 1 Gb of sequencing data (including the library preparation) was 193 USD for PacBio Sequel I, 97 USD for ONT PromethION, and 12 USD for BGI stLFR (raw reads subsequently used in assembly). Virtual long reads were generated using the stLFR protocol. This technology benefits from the accuracy and the low cost of a short-read sequencing platform while providing long-range information. It was the cheapest and most accurate approach as it generated an assembly with the fewest single base and indel errors."

The claim of "the cheapest and most accurate approach" is very strong and it is not supported by the presented results.
The reasons for my concerns are the following:

1. From the presented data, it is clear that BGI stLFR technology achieves an assembly with fewer single base and indel errors in comparison with an assembly generated from Illumina reads. However, this is just one of the measures and I deem that using just this one is not enough to support the claim that this is the most accurate approach. Furthermore, using the same reasoning, one might conclude that Illumina technology is the cheapest and most accurate.

We thank the reviewer for this comment. The main text has been reworded:
• In the 'stLFR genome assembly' section, the sentence "The stLFR assembly was the most accurate with the lowest number of mismatches and indels identified as compared to the Illumina short-read assembly" has been modified to "When compared to the Illumina short-read assembly, the stLFR assembly contained the lowest number of mismatches and indels".
• In the discussion, the sentence "It was the cheapest and most accurate approach as it generated an assembly with the fewest single base and indel errors." has been modified to "It was the cheapest approach and it generated an assembly with the fewest single base and indel errors".

2. Assessing the quality of de novo reconstructed genome is not straightforward. However, there are standard measures such as contiguity, the calculation of duplication rate, k-mer spectra. BGI assemblies without gap filling with ONT or PacBio reads are highly fragmented. Better than Illumina, but not comparable with other technologies. Once a new genome is assembled, the subsequent essential analysis is annotation. If one looks from this perspective, an important measure of the quality is BUSCO

score. Using PacBio data only, most assemblers achieve better BUSCO scores than Supernova with BGI stLFR reads. Using PacBio + Illumina and ONT + Illumina also lead to better BUSCO scores. It is important to emphasize that the assembly produced using Illumina reads only has the worst BUSCO score.

The following sentence has been added to the discussion to emphasize that the assembly produced using Illumina reads only has the worst BUSCO score: "The three long-read sequencing technologies significantly improved the assembly completeness as compared to the assembly produced using the Illumina reads only (65% of complete BUSCOs)."

3. Figures and tables in the main document are one of the most important parts of a manuscript because it is easy to memorize them. Figures 1, 2, and 3 are essential for understanding the assembly quality. Yet, results achieved with BGI technology are not presented in Figure 1. Furthermore, it is not clear why authors take only Flye for ONT reads, and Falcon for PacBio reads to comparison regarding BUSCO score and indels and mismatches count. It might be better if they took more assemblers for each of the read sets. Differences for ONT reads are tiny. Flye has slightly better BUSCO results for ONT and ONT + Illumina than Canu and Raven but also has somewhat worse results for indels and mismatches in comparison to Redbean and Raven. The need for more presented tools is even more evident for PacBio reads. Although Flye achieves slightly worse BUSCO score than Falcon, it is by far best tool for PacBio data measuring indels and mismatches, and produced results are very near to results achieved by BGI stLFR technology.

We agree with the reviewer regarding the suggestion to include all the tools benchmarked in the main figures and tables.
• Figure 1 has been modified to include the results of the BGI assembly.
• Figures 2 and 3 have been modified to include all the assemblers benchmarked.
• The Table 2 has been removed as it was only showing some of the assemblies generated. The text now referred the reader to the supplementary tables containing the results for all the assemblers tested (Table S2 and S4).

4. It is evident that BGI stLFR technology is cheaper than PacBio and ONT regarding the sequencing cost, but the total cost of the assembly should include the computational cost. The computational cost is often unjustly neglected, although it could be substantial and sometimes even higher than sequencing cost. In my previous review, I emphasized the need for the inclusion of the computational cost in the total cost. A significant number of researchers perform their analysis in the cloud, and they would be interested in having an estimation of this cost. It should include all computationally intensive tasks from basecalling, production of consensus reads, assembly, and polishing. Since authors have already measured the performance (CPU hours and memory), the cost might be easily calculated comparing available machines and clusters with the servers of similar performances in the cloud.

We agree with the reviewer that the computational cost is an important parameter that should be included in the total cost to generate a genome assembly. We used the Amazon EC2 On-Demand pricing online resources to compute estimated cost for each technology by comparing to the available servers of similar performances in the cloud.
• Table S11 provides an estimation of the cost to generate the final polished assembly per technology including the assembly, polishing and gap filling steps. We did not include the ONT basecalling step as this task was performed directly on the PromethION machine. Similarly, the generation of subreads was performed on the PacBio Sequel instrument by the sequencing facility.
• Estimated cost to run each assembler (SPAdes, Redbean, Flye, Raven, MaSuRCA) is provided in the Tables S1, S2 and S4.

Minor comments.
In supplementary files:
- labels for specific assemblers are non-consistent. It could mislead the reader
We thank the reviewer for this comment. The labels for the assemblers in the Figure S1 are now consistent between the panels A and B.

- x-scales of the figures are different (e.g., Figure 2). Therefore it is difficult to compare results
The x-scales in the Figures S1 and S2 are now similar between the different panels.

The authors have made a great analysis of available technologies, and I argue that there is a need for such a comprehensive analysis for a plant genome. If they provide the required data and, in their

conclusion, precisely compare different technologies supported by data, I will support the publication of this manuscript.

Close
