# Supplementary material for: Comparison of long-read methods for sequencing and assembly of a plant genome
Source: Gigascience. 2020 Dec 21;9(12):giaa146. doi: 10.1093/gigascience/giaa146 (PMC7751402; doi:10.1093/gigascience/giaa146)
Supplement: giaa146_Response_to_Reviewer_Comments_Revision_2 [file giaa146_response_to_reviewer_comments_revision_2.pdf]

Author's Response To Reviewer Comments

Close

Dear Hongling,

We included the changes to the manuscript text following the comments in the PDF version you sent by email. Please find attached the LaTex version of the manuscript and the bibliography .bib file.

Kind regards,
Valentine Murigneux

Close
