# Supplementary material for: Comparison of long-read methods for sequencing and assembly of a plant genome
Source: Gigascience. 2020 Dec 21;9(12):giaa146. doi: 10.1093/gigascience/giaa146 (PMC7751402; doi:10.1093/gigascience/giaa146)
Supplement: giaa146_Reviewer_1_Report_Original_Submission — CÃ©cile Monat, Ph.D. -- 4/20/2020 Reviewed [file giaa146_reviewer_1_report_original_submission.pdf]

Reviewer Report

Title: Comparison of long read methods for sequencing and assembly of a plant genome

Version: Original Submission **Date:** 4/20/2020

Reviewer name: CÃ©cile Monat, Ph.D.

Reviewer Comments to Author:

Introduction part:
- It would be nice to put the genome size and to indicate the reference genome that is already sequenced and assembled for Macadamia, just to put a context for the people who are not familiar with Macadamia.
Methods part:
- ONT library preparation and sequencing part:
    - What was the reason to used both MinION and PromethION and not only PromethION?
    - For what reason didn't you use the same version of MinKNOW to assemble the MinION (MinKNOW (v1.15.4)) and PromethION (MinKNOW (v3.1.23)) data?
- Assembly of genomes part:
    - Is there a reason for doing 4 iterations of Racon? And not 3 or 5?
    - Maybe you should precise that Racon is used as an error-correction module and Medaka to create the consensus sequence.
    - "Hybrid assembly was generated with MaSuRCA v3.3.3 (MaSuRCA, RRID:SCR_010691) [32] using the Illumina and the ONT or PacBio reads and using Flye v2.5 to perform the final assembly of corrected mega-reads" this sentence is not very clear to me. Does it mean that you have first used ONT/PacBio data + Illumina on MaSuRCA software to generate what they call "super-reads" and then from this data you used Flye to get the final assemblies?
    - as I understood stLFR is similar to 10x genomics, why not compare this technology data too?
- Assembly comparison part:
    - "We compared the assemblies with the published reference genome of Macadamia integrifolia v2 (Genbank accession: GCA_900631585.1)." First, I think it is important to add the reference paper. Secondly, I cannot see where did you compare your assemblies with the one published? For me, you compared all your assemblies between each other, but I cannot find any other assembly.
    - when you said "Illumina assembly" do you refer to the Macadamia integrifolia assembly? If so, please clarify it in the rest of the paper, and add the data for this reference genome in your figures.
Results part:
- ONT genome assembly part:
    - Is there any interested to combine MinION and PromethION data? Are there any advantages to combining it?
    - "The genome completeness was slightly better after two iterations of NextPolish (95.5%) than after two iterations of Pilon (95.2%) (Sup Table 1)." Here I would precise that it is the case for the Flye assembly, but surprisingly (at least for me?) after two iterations of NextPolish on the Canu assembly, the

results were a little less good as with one iteration. So, depending on the assembler you use, the number of iteration needed might be different.

- "As an estimation of the base accuracy, we computed the number of mismatches and indels as compared to the Illumina assembly." Here I am not sure which assembly you refer to when you use the "Illumina assembly" term. Do you refer to the Macadamia integrifolia assembly or to the MaSuRCA hybrid assembly? If you refer to the last one, I would suggest using the word hybrid assembly instead of Illumina assembly, it might be confusing.
- Why not using the Pilon and NextPolish step on the ONT+Illumina (MaSuRCA) assembly since they are tools dedicated to long and short reads polishing?

- PacBio genome assembly part:
    - Why did you use FALCON as the assembler for PacBio but not for ONT? If I am correct, it is not uniquely build to work on PacBio data but is ok for all long-reads technologies.
    - "Two subsets of reads corresponding to 4 SMRT cells and equivalent to a 43Ã— and 39Ã— coverage were assembled using Flye." why choosing Flye for this analysis? I'm also wondering if this part is necessary since afterward, you do the ONT equivalent coverage which is more interesting for the comparison of the technologies.
    - Comment on the structure: for this paragraph, I would prefer to have first the result with the same assemblers as with the ONT data, and then an explanation of why you choose to perform also a test with FALCON and then the FALCON results.
- stLFR genome assembly part:
    - Supernova might have been used on PacBio data as well, why not?
    - why not trying to complement PacBio data with stLFR as you did with ONT? Are there any incompatibilities?

Discussion part:

- "The amount of sequencing data produced by each platform corresponds to approximately 84Ã— (PacBio Sequel), 32Ã— (ONT) and 96Ã— (BGI stLFR) coverage of the macadamia genome" I would have put this information into the Results part, but it's only my preference.
- "For both ONT and PacBio data, the highest assembly contiguity was obtained with a long-read only assembler as compared to an hybrid assembler incorporating both the short and long reads." I would suggest using the term "long-read polished" instead of "long-read only" since the assembly with the best contiguity integrates the Illumina data for the polishing.

Tables and figures:

- Table 2:
    - For this figure, if I understood properly you have chosen the best assembly of each technology. If I am right, then please precise it in the title of the figure.
- Figure 1:
    - If I understood properly and here when you write "Base accuracy of assemblies as compared to Illumina assembly" you refer to the Macadamia integrifolia assembly, then I would add the Macadamia integrifolia assembly in this figure, and maybe put a dotted line at the limit of it for each category (InDels and mismatches) so it is easier for the reader to compare with it.
- Figure 2:
    - Here I would put all the assemblies you had in Figure 1

Methods

Are the methods appropriate to the aims of the study, are they well described, and are necessary controls included? Choose an item.

Conclusions

Are the conclusions adequately supported by the data shown? Choose an item.

Reporting Standards

Does the manuscript adhere to the journal's guidelines on minimum standards of reporting? Choose an item.

Choose an item.

Statistics

Are you able to assess all statistics in the manuscript, including the appropriateness of statistical tests used? Choose an item.

Quality of Written English

Please indicate the quality of language in the manuscript: Choose an item.

Declaration of Competing Interests

Please complete a declaration of competing interests, considering the following questions:

- Have you in the past five years received reimbursements, fees, funding, or salary from an organisation that may in any way gain or lose financially from the publication of this manuscript, either now or in the future?
- Do you hold any stocks or shares in an organisation that may in any way gain or lose financially from the publication of this manuscript, either now or in the future?
- Do you hold or are you currently applying for any patents relating to the content of the manuscript?
- Have you received reimbursements, fees, funding, or salary from an organization that holds or has applied for patents relating to the content of the manuscript?
- Do you have any other financial competing interests?
- Do you have any non-financial competing interests in relation to this paper?

If you can answer no to all of the above, write 'I declare that I have no competing interests' below. If your reply is yes to any, please give details below.

I declare that I have no competing interests

I agree to the open peer review policy of the journal. I understand that my name will be included on my report to the authors and, if the manuscript is accepted for publication, my named report including any attachments I upload will be posted on the website along with the authors' responses. I agree for my report to be made available under an Open Access Creative Commons CC-BY license (http://creativecommons.org/licenses/by/4.0/). I understand that any comments which I do not wish to be included in my named report can be included as confidential comments to the editors, which will not be published.

Choose an item.

To further support our reviewers, we have joined with Publons, where you can gain additional credit to further highlight your hard work (see: https://publons.com/journal/530/gigascience). On publication of this paper, your review will be automatically added to Publons, you can then choose whether or not to claim your Publons credit. I understand this statement.

Yes Choose an item.
