# Supplementary material for: Comparison of long-read methods for sequencing and assembly of a plant genome
Source: Gigascience. 2020 Dec 21;9(12):giaa146. doi: 10.1093/gigascience/giaa146 (PMC7751402; doi:10.1093/gigascience/giaa146)
Supplement: giaa146_Reviewer_2_Report_Original_Submission — Mile Å ikiÄ‡ -- 5/2/2020 Reviewed [file giaa146_reviewer_2_report_original_submission.pdf]

Reviewer Report

Title: Comparison of long read methods for sequencing and assembly of a plant genome

Version: Original Submission **Date:** 5/2/2020

Reviewer name: Mile Å ikiÄ‡

Reviewer Comments to Author:

In their paper Murigneux et. al. made a comparison of three long-read sequencing technologies applied to the de novo assembly of a plant genome, Macadamia jansenii. They generated sequencing data using Pacific Biosciences (Sequel I), Oxford Nanopore Technologies (PromethION), and BGI (single-tube Long Fragment Read) technologies. Sequenced data are assembled using a bunch of state of the art long-read assemblers and hybrid Masurca assembler.
Although paper is easy to follow, and this kind of analysis is more than welcomed I have several major and minor concerns.
Major concerns
1) The authors use 780 Mbps as the estimated size of the genome. Yet, this is not supported by data. In chapter "Genome size estimation", they present the genome size estimation using K-mer counting, but these sizes are 650 Mbps or less
2) Since the real size of the genome is unknown, It would be worthwhile if authors provide analyses such as those enabled by KAT (Mapleson et al., 2017), which compares the k-mer spectrum of the assembly to the k-mer spectrum of reads (preferably Illumina). For control of the misassembled contigs, authors also might align larger contigs obtained using different tools to compare similarity among them (e.g., using tools such as Gepard or similar).
3) The authors compare assemblies with "Illumina assembly", but it is not clear what that means and why they consider this as a valid comparison.
4) Although they started ONT data analysis with four tools, they perform further analysis on just two tools (Flye and Canu). In addition, for PacBio data, they use three tools (Redbean, Fly and Canu). It is not clear why the authors chose these tools. Canu and Fly have larger N50, larger total length, and the longest contigs. However, this does not take into account possible misassembles. Assemblers might have problems with uncollapsed haplotypes, which can result in assemblies larger than expected. In their recent manuscript, Guiglielmoni et al (https://doi.org/10.1101/2020.03.16.993428) showed that Canu is prone to uncollapsed haplotypes. Also, in this manuscript is presented that using PacBio data Canu produces much longer assemblies than other tools (1.2 Gbps). Therefore, the longer total size of a assembly cannot guaranty a better genome. Furthermore, on ONT data Raven has the second-best initial Busco score (before polishing), and its assembled genome consists of the least number of contigs. Therefore, I deem that the full analysis needs to performed using all tools for both Nanopore and Pacbio data.
5) It would be of interest to a broad community if authors add the computational costs in total cost per genome for each sequencing technolgy. They might compare their machines with AWS or other cloud specified configurations. Besides, it is not clear which types of machines they used. Information from

supplementary materials such as GPU, large memory, HPC is not descriptive enough.
Minor comments:
1) The authors use the published reference genome of Macadamia integrifolia v2 for comparison. It would be interesting if they can provide us with information about sequencing read technology used for this assembly.
2) The authors mentioned that the newer generation of PacBio sequencing technology (Sequel II) which provides higher accuracy and lower costs. It would also be worth to mention the newer generations of assembly tools such as Canu 2.0, Raven v1.1.5 or Flye Version 2.7.1
It is worth considering Racon for polishing with Illumina reads too. Yet, this is not a requirement, because authors already use state of the art tools.

Methods

Are the methods appropriate to the aims of the study, are they well described, and are necessary controls included? Choose an item.

Conclusions

Are the conclusions adequately supported by the data shown? Choose an item.

Reporting Standards

Does the manuscript adhere to the journal's guidelines on minimum standards of reporting? Choose an item.

Choose an item.

Statistics

Are you able to assess all statistics in the manuscript, including the appropriateness of statistical tests used? Choose an item.

Quality of Written English

Please indicate the quality of language in the manuscript: Choose an item.

Declaration of Competing Interests

Please complete a declaration of competing interests, considering the following questions:

- Have you in the past five years received reimbursements, fees, funding, or salary from an organisation that may in any way gain or lose financially from the publication of this manuscript, either now or in the future?

- Do you hold any stocks or shares in an organisation that may in any way gain or lose financially from the publication of this manuscript, either now or in the future?
- Do you hold or are you currently applying for any patents relating to the content of the manuscript?
- Have you received reimbursements, fees, funding, or salary from an organization that holds or has applied for patents relating to the content of the manuscript?
- Do you have any other financial competing interests?
- Do you have any non-financial competing interests in relation to this paper?

If you can answer no to all of the above, write 'I declare that I have no competing interests' below. If your reply is yes to any, please give details below.

I declare that I have no competing interests

I agree to the open peer review policy of the journal. I understand that my name will be included on my report to the authors and, if the manuscript is accepted for publication, my named report including any attachments I upload will be posted on the website along with the authors' responses. I agree for my report to be made available under an Open Access Creative Commons CC-BY license (http://creativecommons.org/licenses/by/4.0/). I understand that any comments which I do not wish to be included in my named report can be included as confidential comments to the editors, which will not be published.

Choose an item.

To further support our reviewers, we have joined with Publons, where you can gain additional credit to further highlight your hard work (see: https://publons.com/journal/530/gigascience). On publication of this paper, your review will be automatically added to Publons, you can then choose whether or not to claim your Publons credit. I understand this statement.

Yes Choose an item.
