# Supplementary material for: Comparison of long-read methods for sequencing and assembly of a plant genome
Source: Gigascience. 2020 Dec 21;9(12):giaa146. doi: 10.1093/gigascience/giaa146 (PMC7751402; doi:10.1093/gigascience/giaa146)
Supplement: giaa146_Reviewer_2_Report_Revision_1 — Mile Å ikiÄ‡ -- 7/26/2020 Reviewed [file giaa146_reviewer_2_report_revision_1.pdf]

Reviewer Report

Title: Comparison of long read methods for sequencing and assembly of a plant genome

Version: Revision 1 **Date:** 7/26/2020

Reviewer name: Mile Å ikiÄ‡

Reviewer Comments to Author:

The authors have answered most of the comments from my previous review adequately. My remaining concern is mostly related to the following paragraph:
"The cost of generating 1 Gb of sequencing data (including the library preparation) was 193 USD for PacBio Sequel I, 97 USD for ONT PromethION, and 12 USD for BGI stLFR (raw reads subsequently used in assembly). Virtual long reads were generated using the stLFR protocol. This technology benefits from the accuracy and the low cost of a short-read sequencing platform while providing long-range information. It was the cheapest and most accurate approach as it generated an assembly with the fewest single base and indel errors."
The claim of "the cheapest and most accurate approach" is very strong and it is not supported by the presented results.
The reasons for my concerns are the following:

1. From the presented data, it is clear that BGI stLFR technology achieves an assembly with fewer single base and indel errors in comparison with an assembly generated from Illumina reads. However, this is just one of the measures and I deem that using just this one is not enough to support the claim that this is the most accurate approach. Furthermore, using the same reasoning, one might conclude that Illumina technology is the cheapest and most accurate.
2. Assessing the quality of de novo reconstructed genome is not straightforward. However, there are standard measures such as contiguity, the calculation of duplication rate, k-mer spectra. BGI assemblies without gap filling with ONT or PacBio reads are highly fragmented. Better than Illumina, but not comparable with other technologies. Once a new genome is assembled, the subsequent essential analysis is annotation. If one looks from this perspective, an important measure of the quality is BUSCO score. Using PacBio data only, most assemblers achieve better BUSCO scores than Supernova with BGI stLFR reads. Using PacBio + Illumina and ONT + Illumina also lead to better BUSCO scores. It is important to emphasize that the assembly produced using Illumina reads only has the worst BUSCO score.
3. Figures and tables in the main document are one of the most important parts of a manuscript because it is easy to memorize them. Figures 1, 2, and 3 are essential for understanding the assembly quality. Yet, results achieved with BGI technology are not presented in Figure 1. Furthermore, it is not clear why authors take only Flye for ONT reads, and Falcon for PacBio reads to comparison regarding BUSCO score and indels and mismatches count. It might be better if they took more assemblers for each of the read sets. Differences for ONT reads are tiny. Flye has slightly better BUSCO results for ONT and ONT + Illumina than Canu and Raven but also has somewhat worse results for indels and mismatches in comparison to Redbean and Raven. The need for more presented tools is even more evident for PacBio

reads. Although Flye achieves slightly worse BUSCO score than Falcon, it is by far best tool for PacBio data measuring indels and mismatches, and produced results are very near to results achieved by BGI stLFR technology.

4. It is evident that BGI stLFR technology is cheaper than PacBio and ONT regarding the sequencing cost, but the total cost of the assembly should include the computational cost. The computational cost is often unjustly neglected, although it could be substantial and sometimes even higher than sequencing cost. In my previous review, I emphasized the need for the inclusion of the computational cost in the total cost. A significant number of researchers perform their analysis in the cloud, and they would be interested in having an estimation of this cost. It should include all computationally intensive tasks from basecalling, production of consensus reads, assembly, and polishing. Since authors have already measured the performance (CPU hours and memory), the cost might be easily calculated comparing available machines and clusters with the servers of similar performances in the cloud.

Minor comments.

In supplementary files:

- labels for specific assemblers are non-consistent. It could mislead the reader
- x-scales of the figures are different (e.g., Figure 2). Therefore it is difficult to compare results.

The authors have made a great analysis of available technologies, and I argue that there is a need for such a comprehensive analysis for a plant genome. If they provide the required data and, in their conclusion, precisely compare different technologies supported by data, I will support the publication of this manuscript.

Methods

Are the methods appropriate to the aims of the study, are they well described, and are necessary controls included? Choose an item.

Conclusions

Are the conclusions adequately supported by the data shown? Choose an item.

Reporting Standards

Does the manuscript adhere to the journal's guidelines on minimum standards of reporting? Choose an item.

Choose an item.

Statistics

Are you able to assess all statistics in the manuscript, including the appropriateness of statistical tests used? Choose an item.

Quality of Written English

Please indicate the quality of language in the manuscript: Choose an item.

Declaration of Competing Interests

Please complete a declaration of competing interests, considering the following questions:

- Have you in the past five years received reimbursements, fees, funding, or salary from an organisation that may in any way gain or lose financially from the publication of this manuscript, either now or in the future?
- Do you hold any stocks or shares in an organisation that may in any way gain or lose financially from the publication of this manuscript, either now or in the future?
- Do you hold or are you currently applying for any patents relating to the content of the manuscript?
- Have you received reimbursements, fees, funding, or salary from an organization that holds or has applied for patents relating to the content of the manuscript?
- Do you have any other financial competing interests?
- Do you have any non-financial competing interests in relation to this paper?

If you can answer no to all of the above, write 'I declare that I have no competing interests' below. If your reply is yes to any, please give details below.

I declare that I have no competing interests

I agree to the open peer review policy of the journal. I understand that my name will be included on my report to the authors and, if the manuscript is accepted for publication, my named report including any attachments I upload will be posted on the website along with the authors' responses. I agree for my report to be made available under an Open Access Creative Commons CC-BY license (http://creativecommons.org/licenses/by/4.0/). I understand that any comments which I do not wish to be included in my named report can be included as confidential comments to the editors, which will not be published.

Choose an item.

To further support our reviewers, we have joined with Publons, where you can gain additional credit to further highlight your hard work (see: https://publons.com/journal/530/gigascience). On publication of this paper, your review will be automatically added to Publons, you can then choose whether or not to claim your Publons credit. I understand this statement.

Yes Choose an item.
